# Supplementary figures and images for: Identification of ERAD-dependent degrons for the endoplasmic reticulum lumen
Source: eLife. 2024 Nov 12;12:RP89606. doi: 10.7554/eLife.89606 (PMC11556787; doi:10.7554/eLife.89606)

Figure 1. Identification of endoplasmic reticulum localized degrons.

B

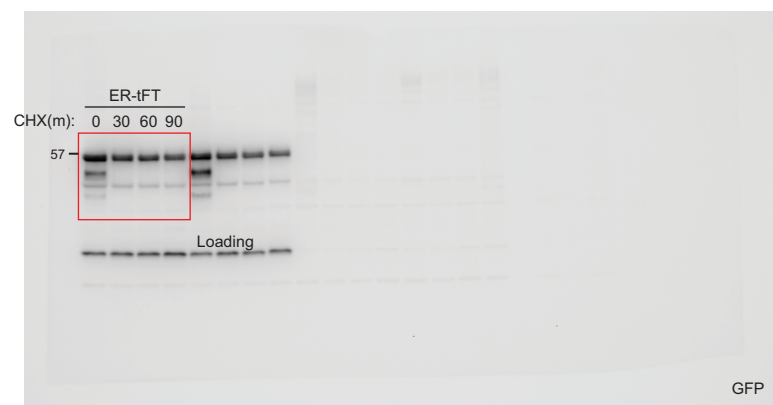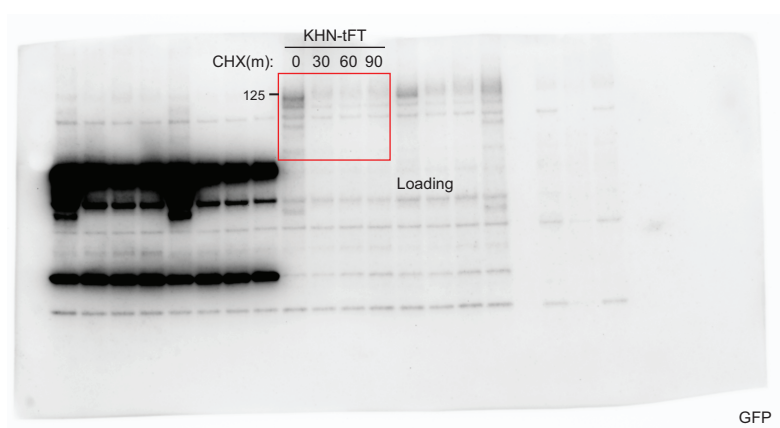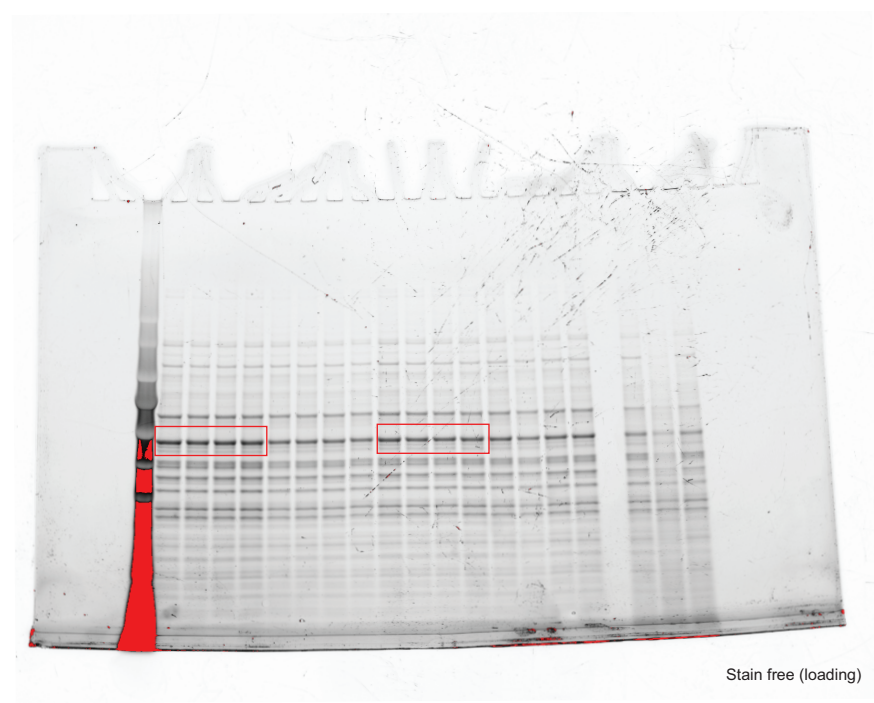

Supplement: Figure 1—source data 1. [file elife-89606-fig1-data1.zip › Figure 1-source data 1.pdf]

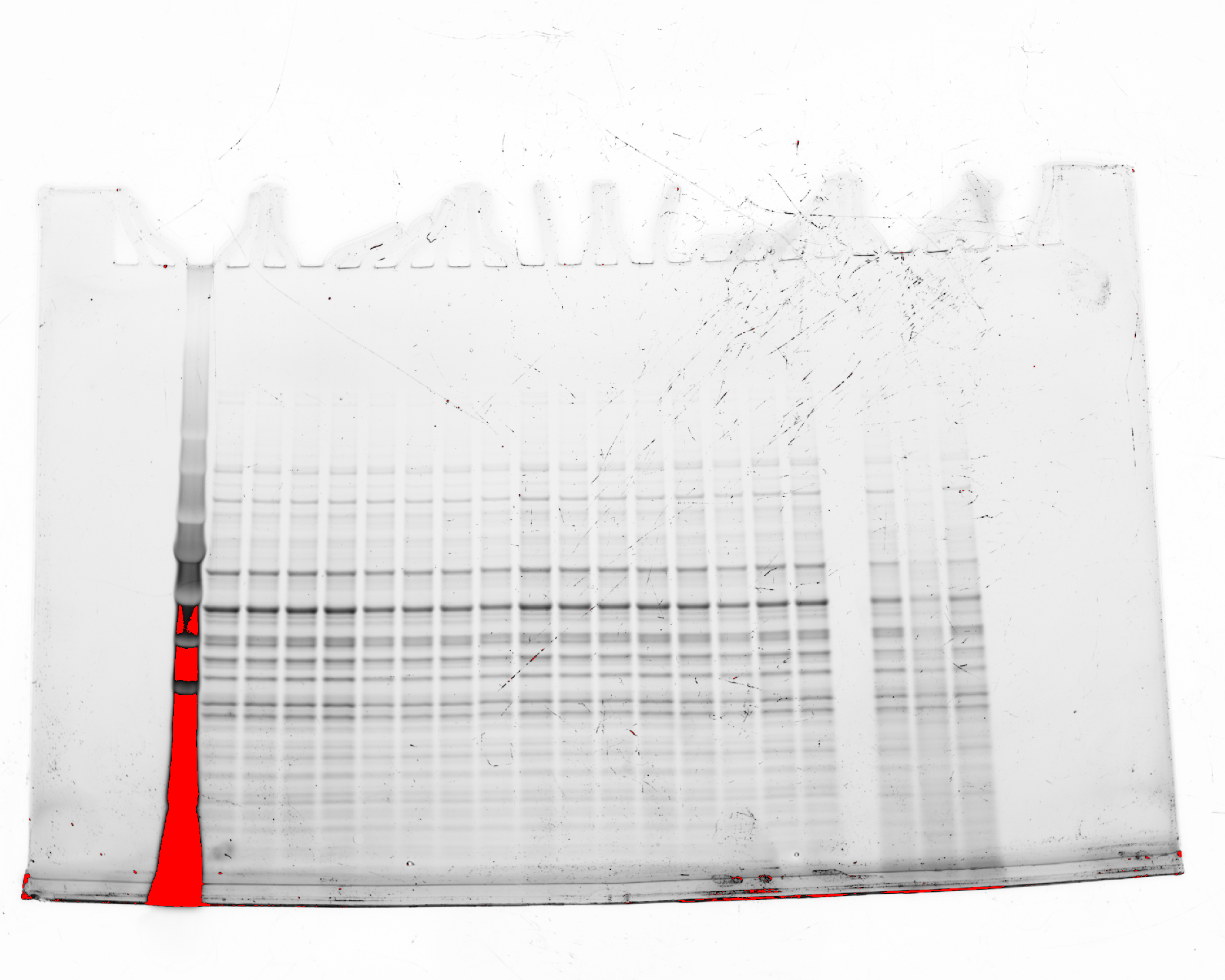

Supplement: Figure 1—source data 2. [file elife-89606-fig1-data2.zip › Figure 1-source data 2/BaldridgeLab 2019-02-19 13h10m18s Stain Free Gel 30.000s(Stain Free Gel).tif]

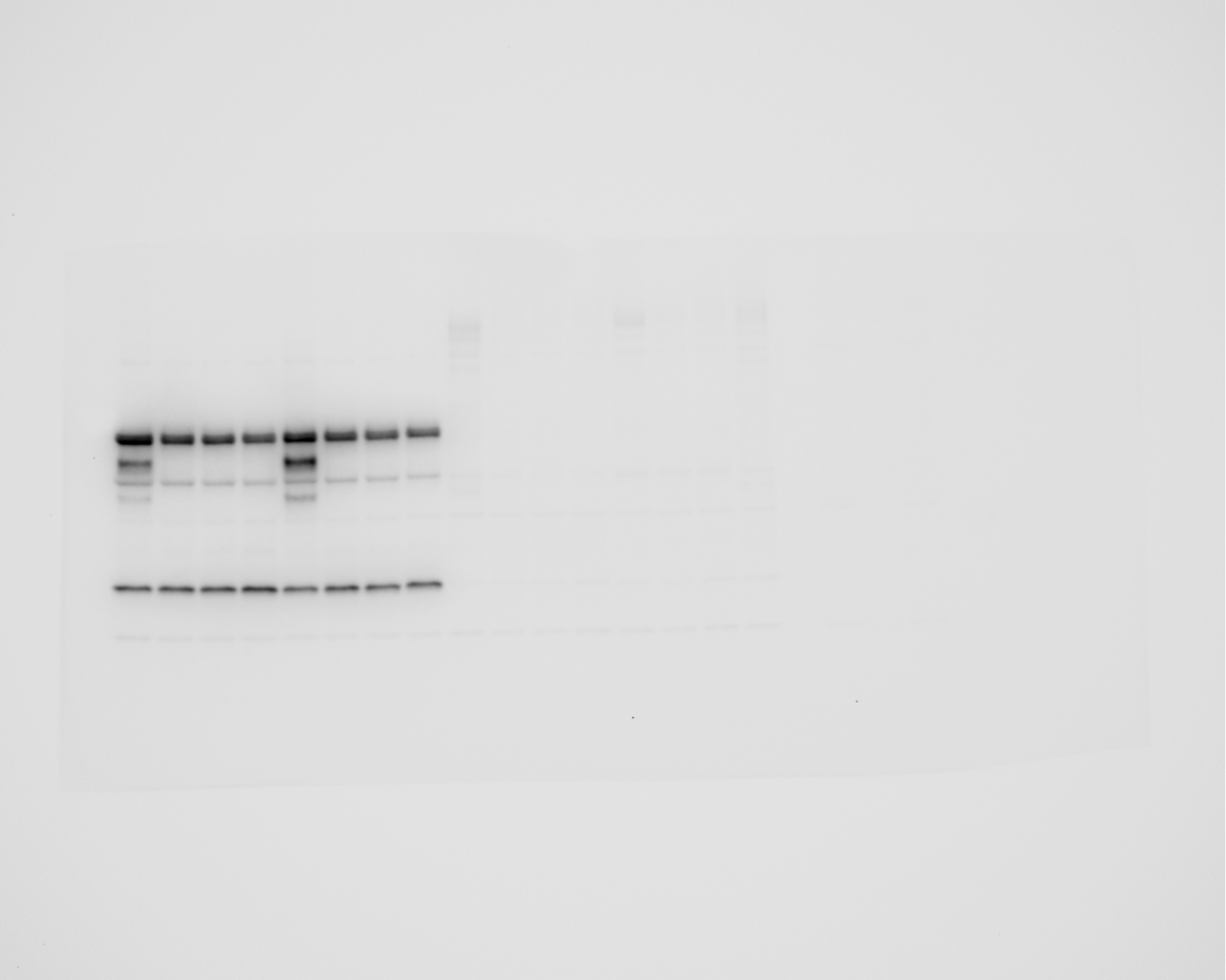

Supplement: Figure 1—source data 2. [file elife-89606-fig1-data2.zip › Figure 1-source data 2/BaldridgeLab 2019-02-19 17h38m56s Chemiluminescence 72.400s(Chemiluminescence).tif]

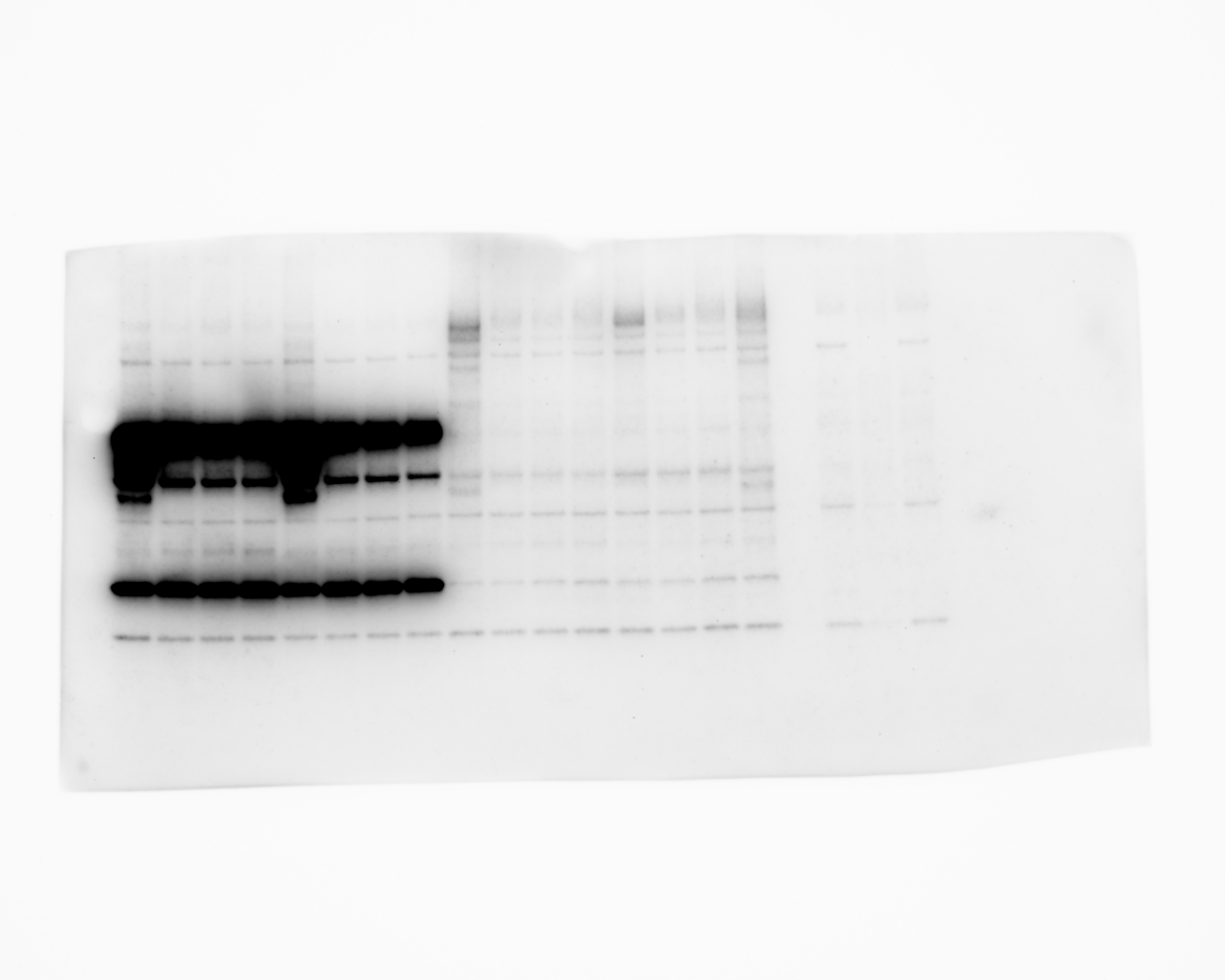

Supplement: Figure 1—source data 2. [file elife-89606-fig1-data2.zip › Figure 1-source data 2/BaldridgeLab 2019-02-19 17h55m20s Chemiluminescence 600.000s(Chemiluminescence).tif]

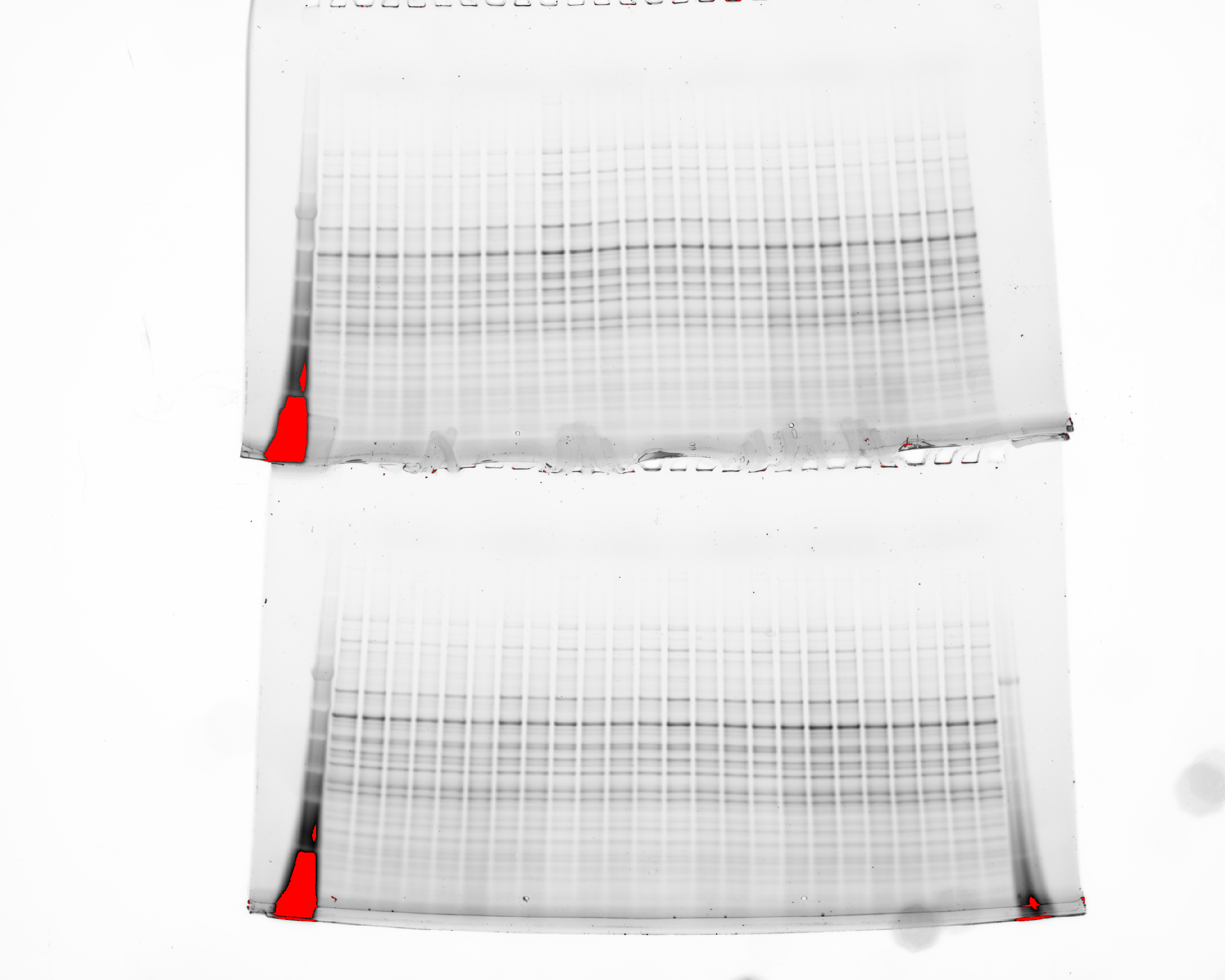

Supplement: Figure 2—source data 2. [file elife-89606-fig2-data2.zip › Figure 2-source data 2/BaldridgeLab 2021-10-21 12h04m07s Stain Free Gel 20.000s(Stain Free Gel).tif]

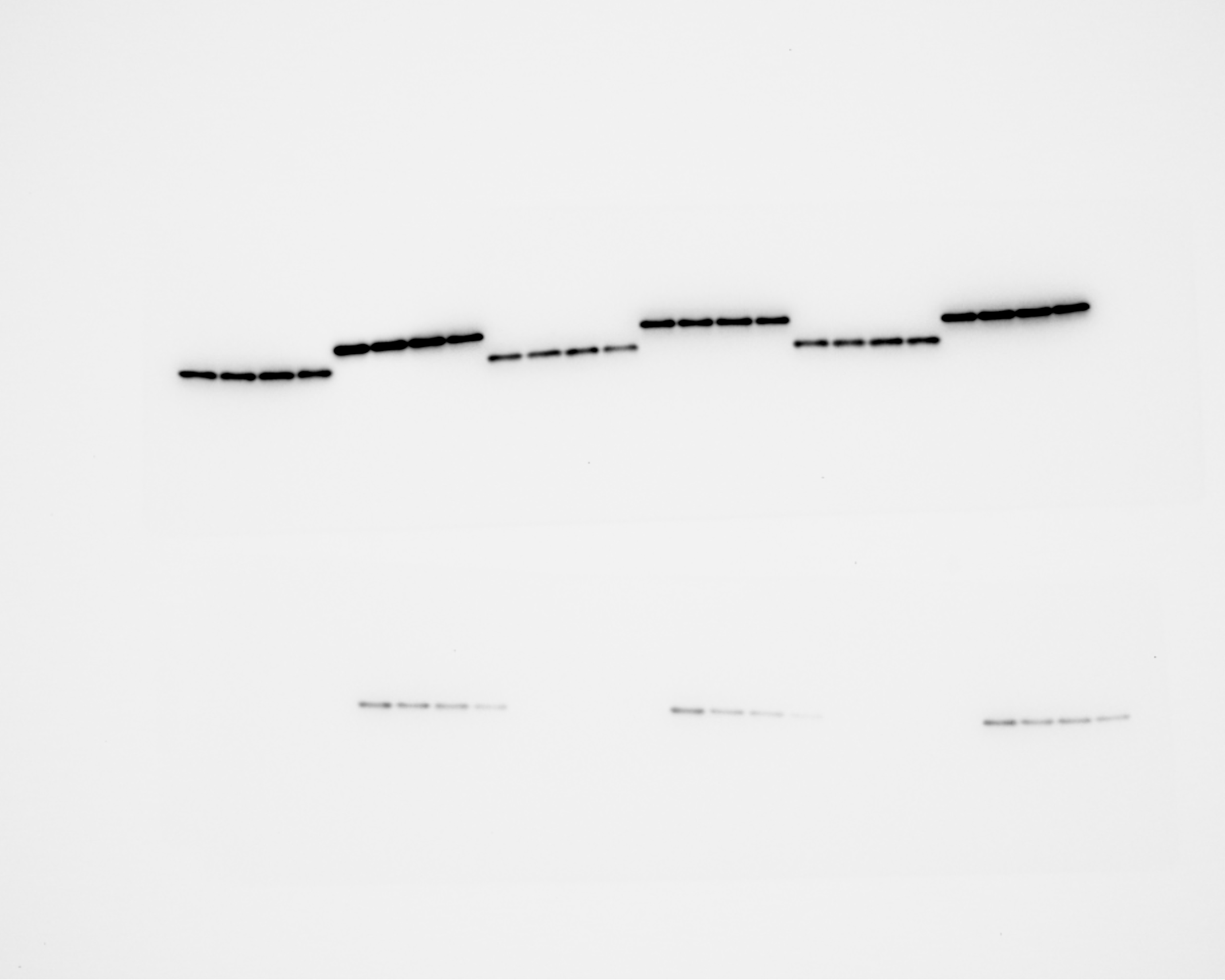

Supplement: Figure 2—source data 2. [file elife-89606-fig2-data2.zip › Figure 2-source data 2/BaldridgeLab 2021-10-21 17h35m41s Chemiluminescence 20.000s(Chemiluminescence).tif]

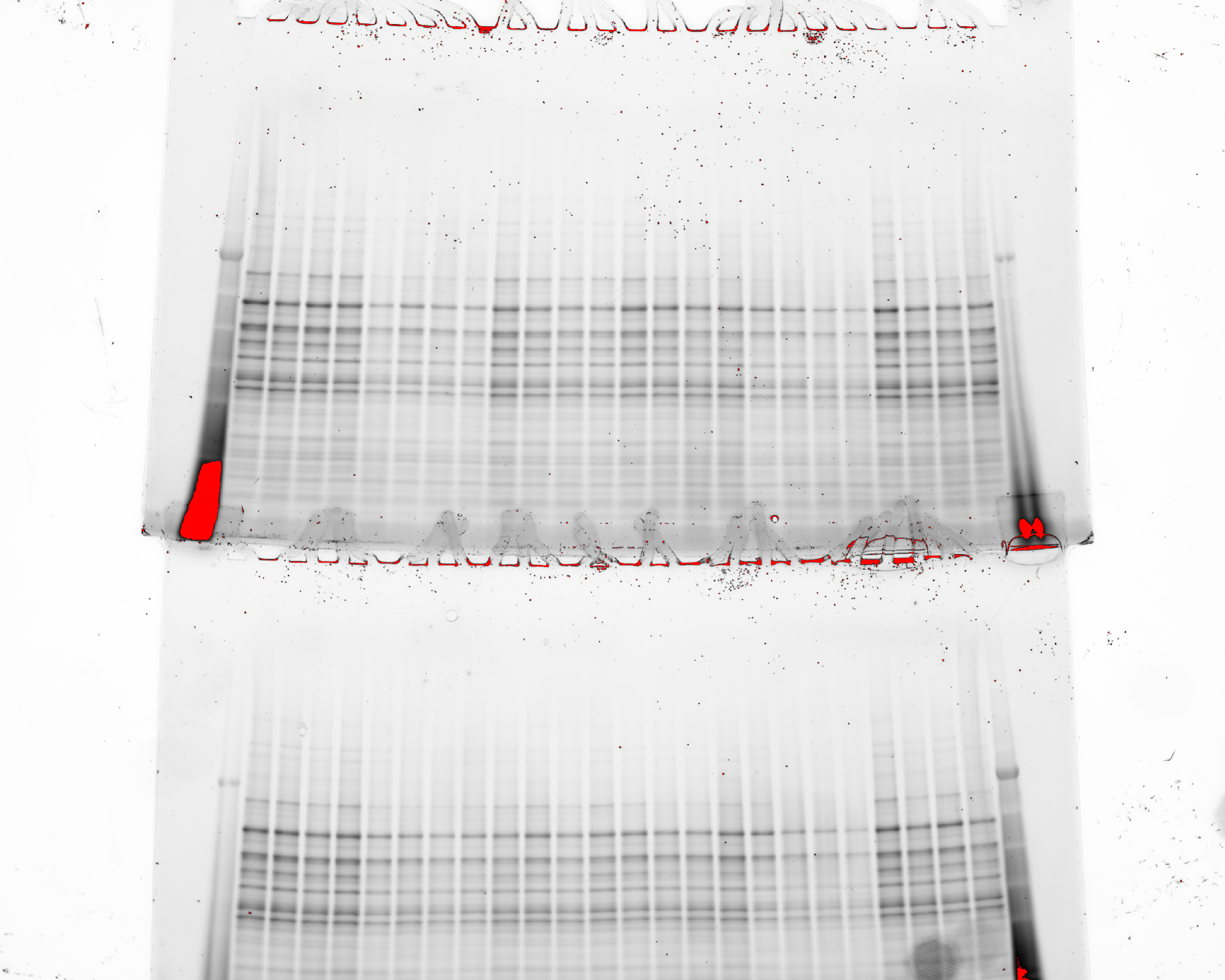

Supplement: Figure 2—source data 2. [file elife-89606-fig2-data2.zip › Figure 2-source data 2/BaldridgeLab 2021-12-16 12h10m45s Stain Free Gel 30.000s(Stain Free Gel).tif]

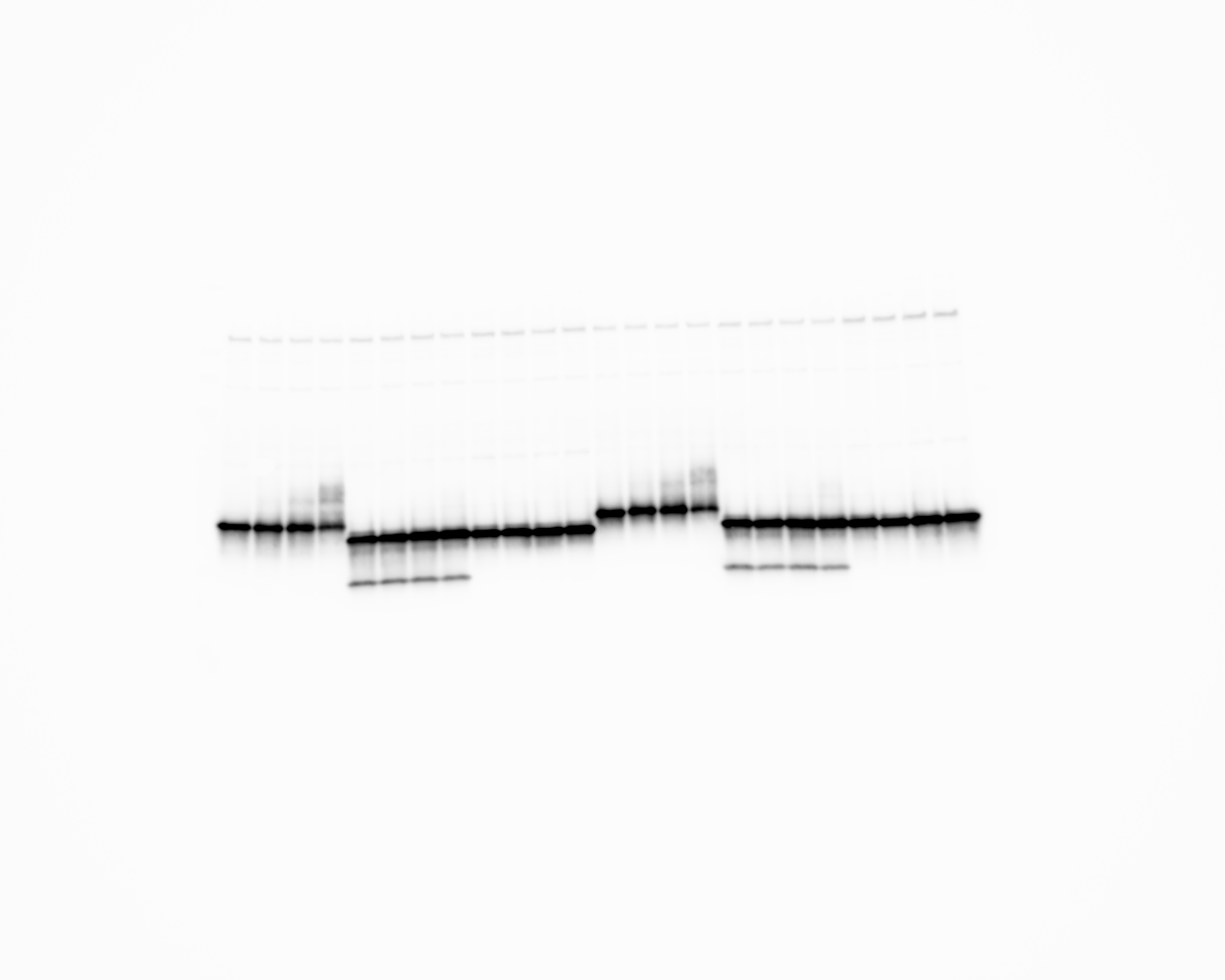

Supplement: Figure 2—source data 2. [file elife-89606-fig2-data2.zip › Figure 2-source data 2/BaldridgeLab 2021-12-16 14h28m45s Chemiluminescence 5.000s(Chemiluminescence).tif]

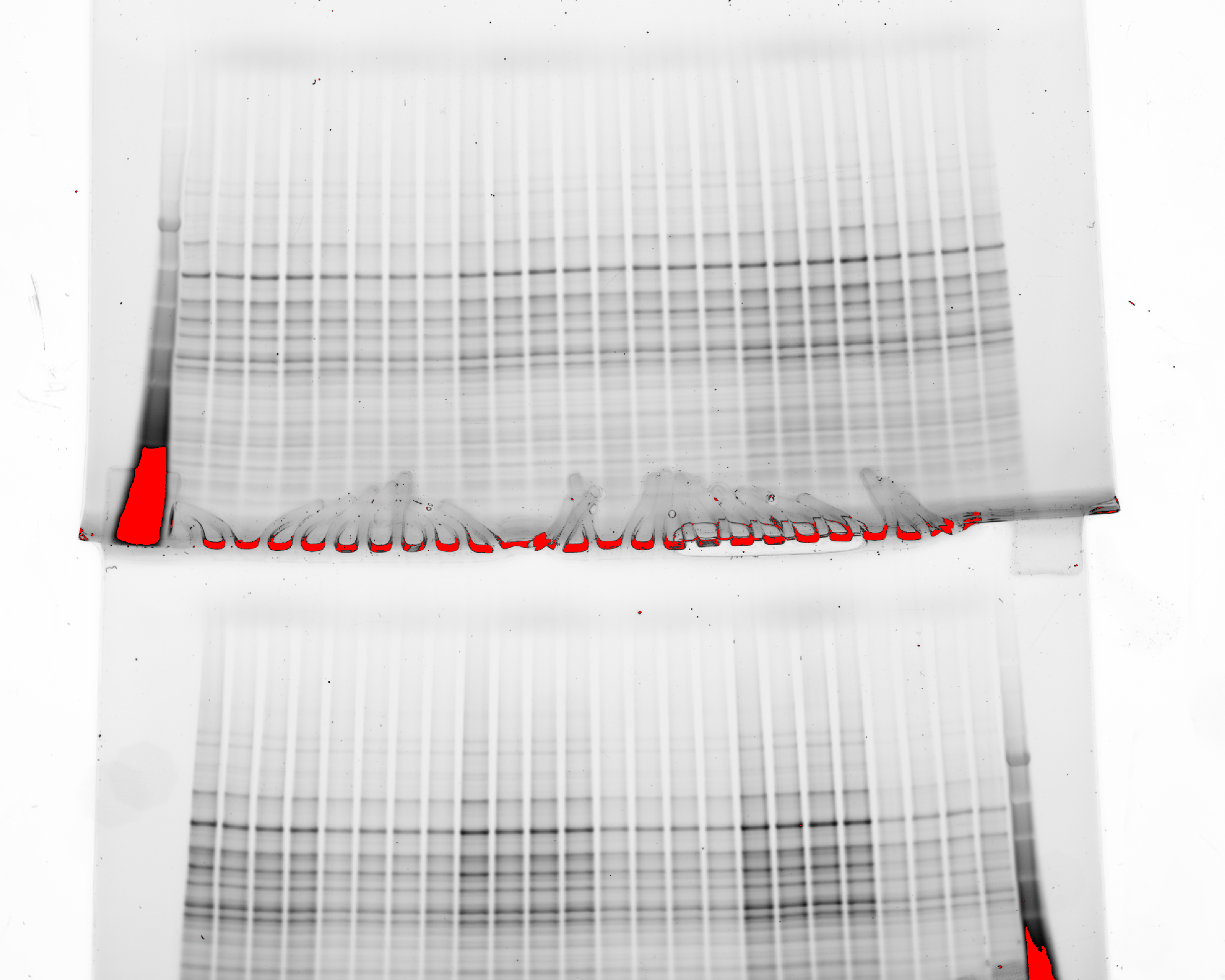

Supplement: Figure 2—source data 2. [file elife-89606-fig2-data2.zip › Figure 2-source data 2/BaldridgeLab 2021-12-20 11h21m44s Stain Free Gel 30.000s(Stain Free Gel).tif]

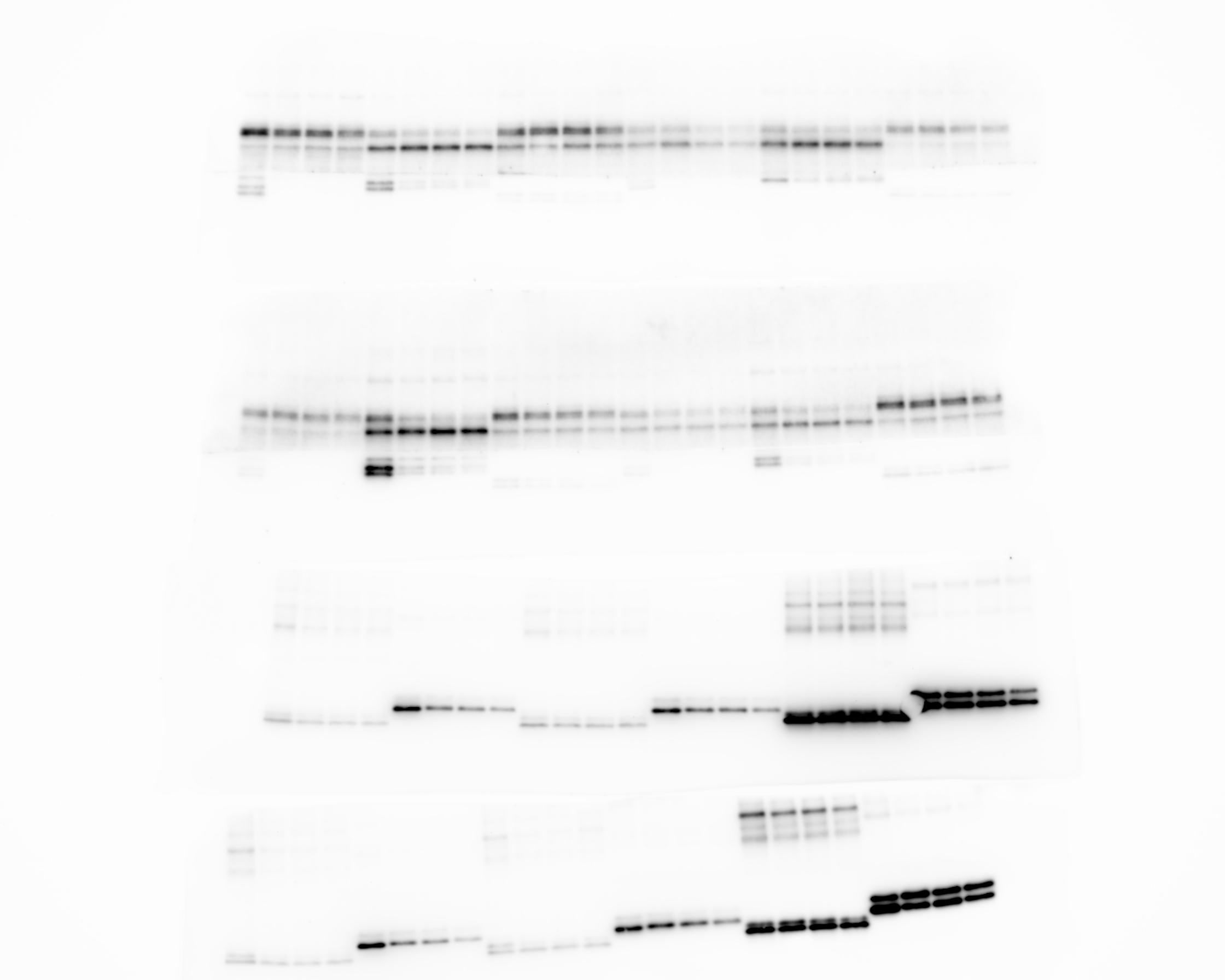

Supplement: Figure 2—source data 2. [file elife-89606-fig2-data2.zip › Figure 2-source data 2/BaldridgeLab 2021-12-20 19h42m04s Chemiluminescence 300.000s(Chemiluminescence).tif]

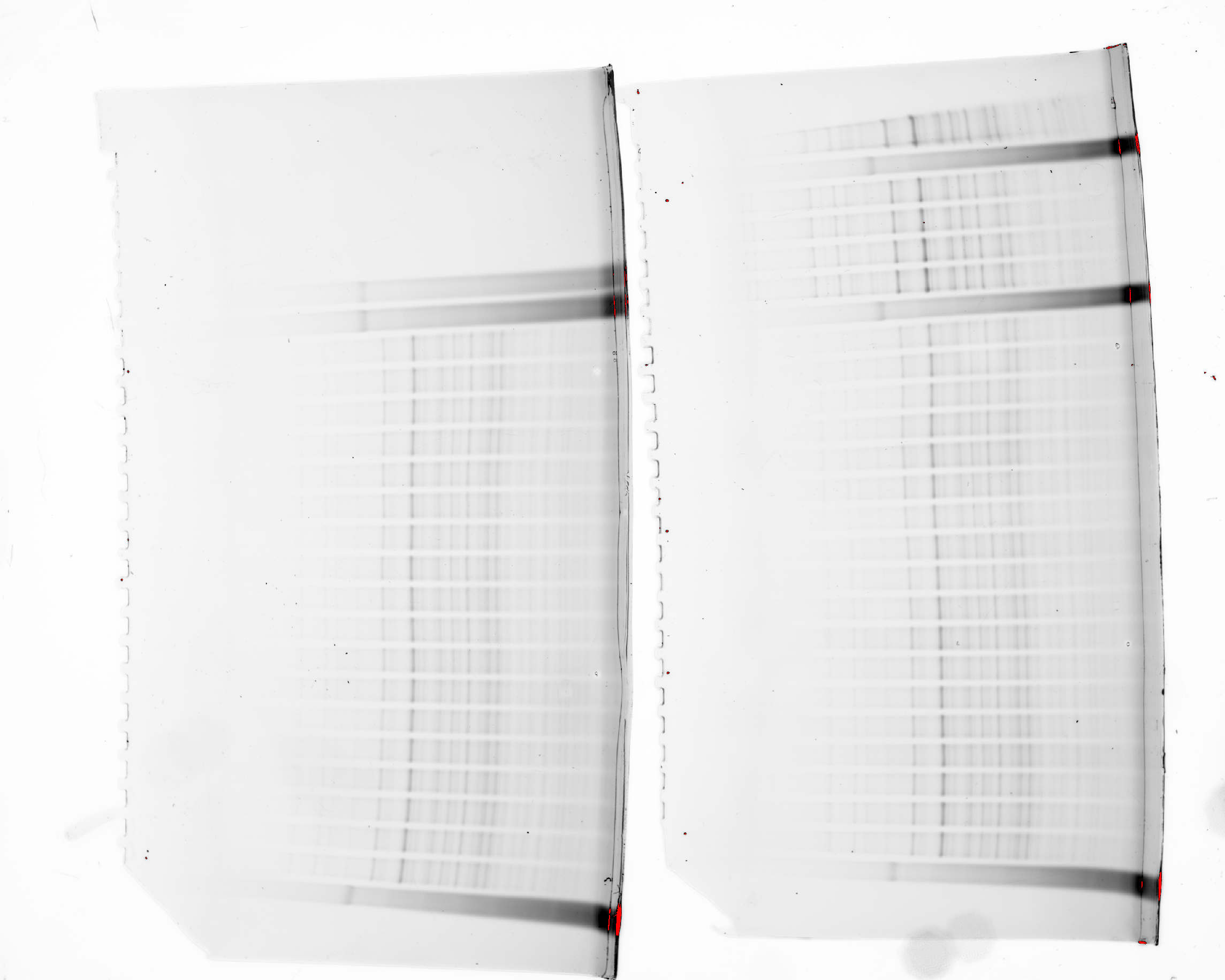

Supplement: Figure 2—source data 2. [file elife-89606-fig2-data2.zip › Figure 2-source data 2/BaldridgeLab 2022-07-20 09h25m26s Stain Free Gel 17.093s(Stain Free Gel).tif]

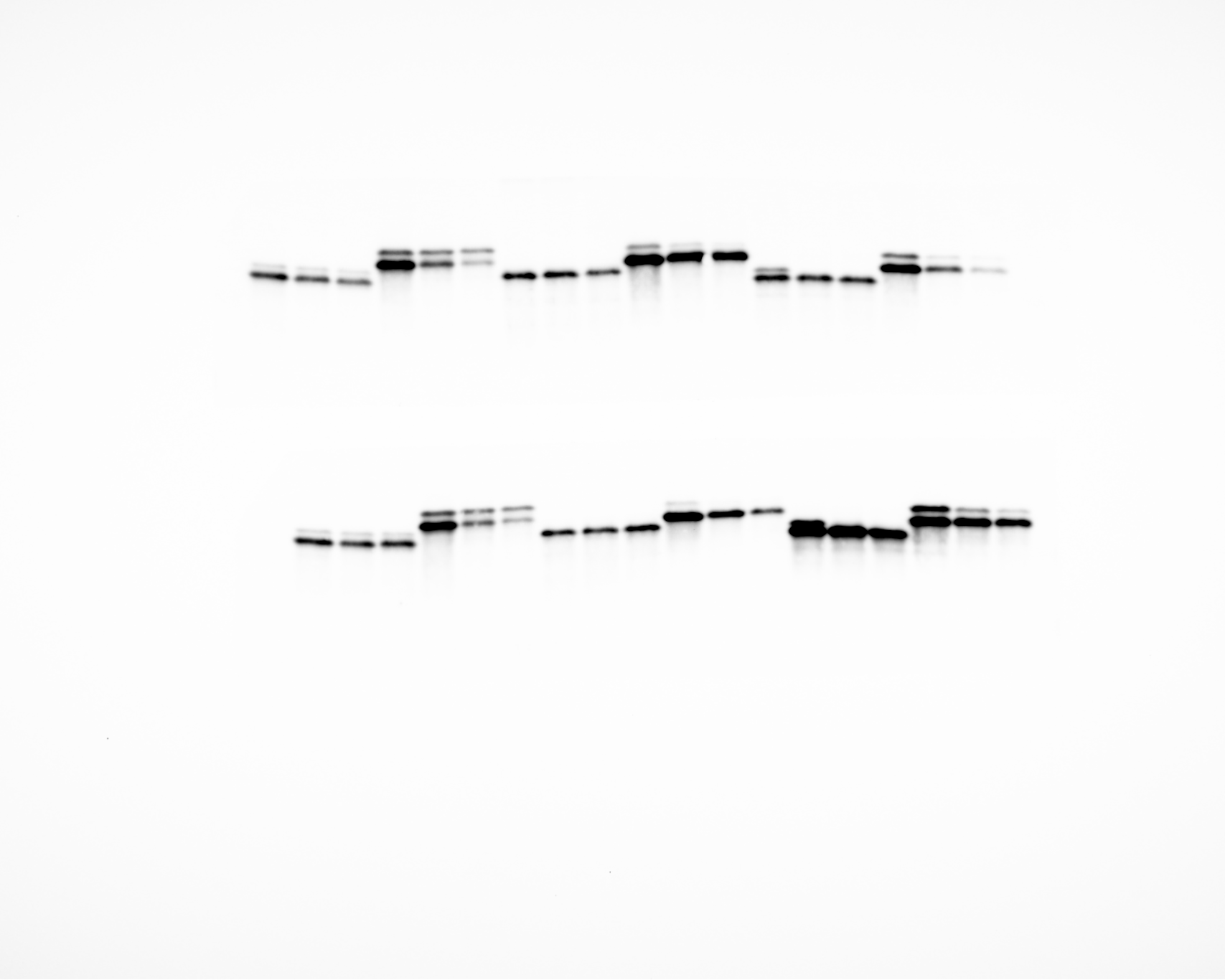

Supplement: Figure 2—source data 2. [file elife-89606-fig2-data2.zip › Figure 2-source data 2/BaldridgeLab 2022-07-21 12h26m51s DyLight 800 10.000s(DyLight 800).tif]

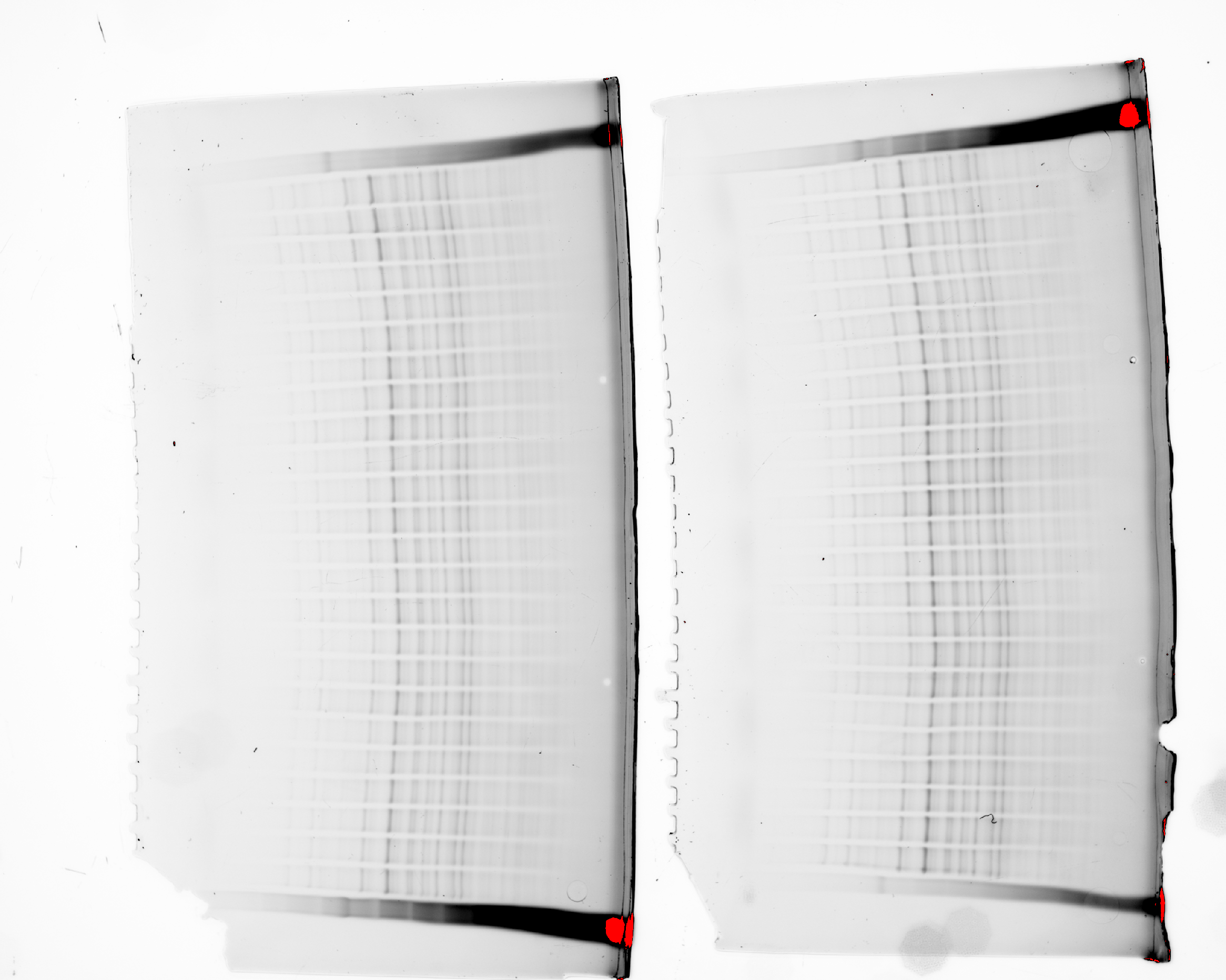

Supplement: Figure 2—source data 2. [file elife-89606-fig2-data2.zip › Figure 2-source data 2/BaldridgeLab 2023-04-26 10h09m07s Stain Free Gel 13.887s(Stain Free Gel).tif]

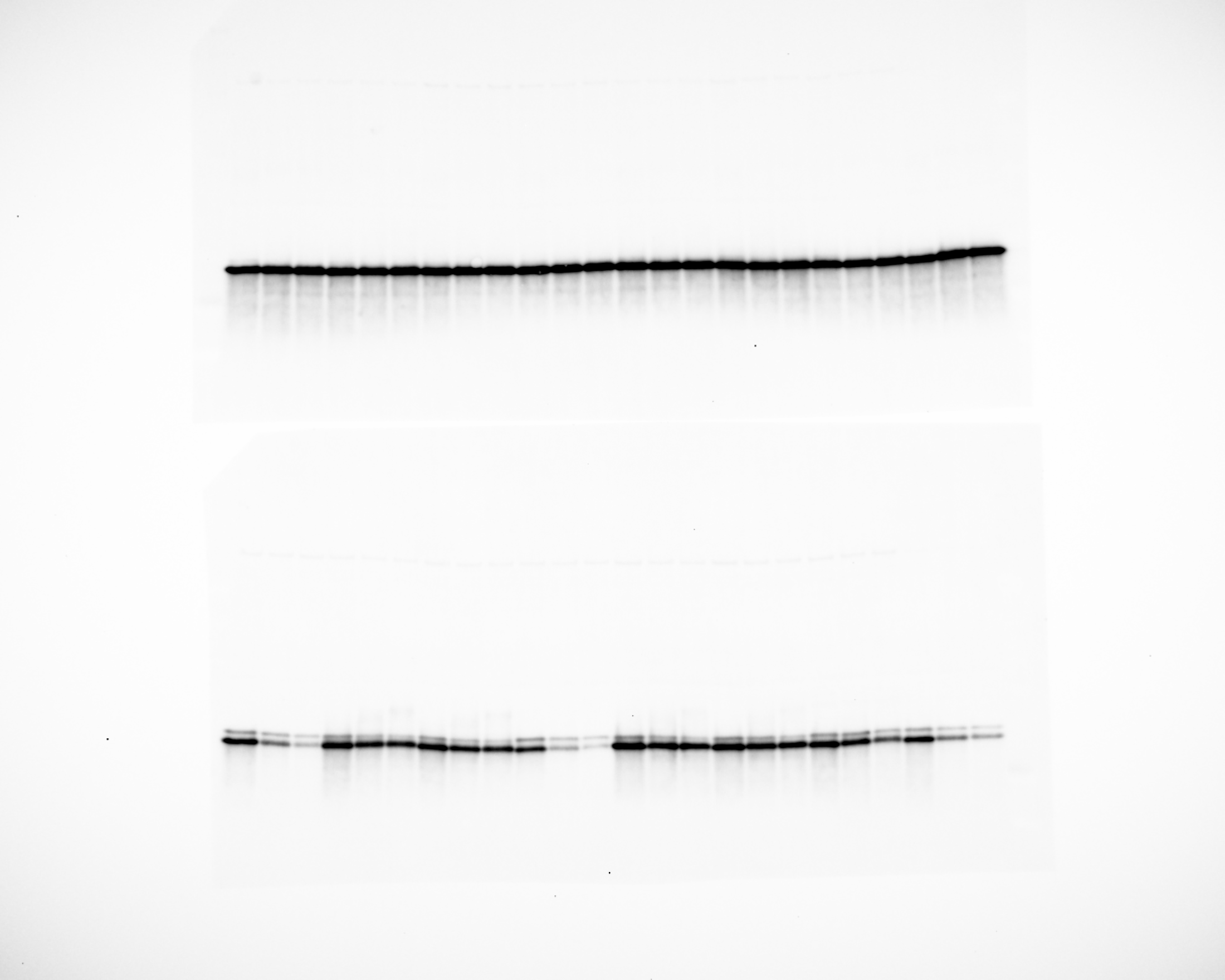

Supplement: Figure 2—source data 2. [file elife-89606-fig2-data2.zip › Figure 2-source data 2/BaldridgeLab 2023-04-26 13h59m16s DyLight 800 30.000s(DyLight 800).tif]

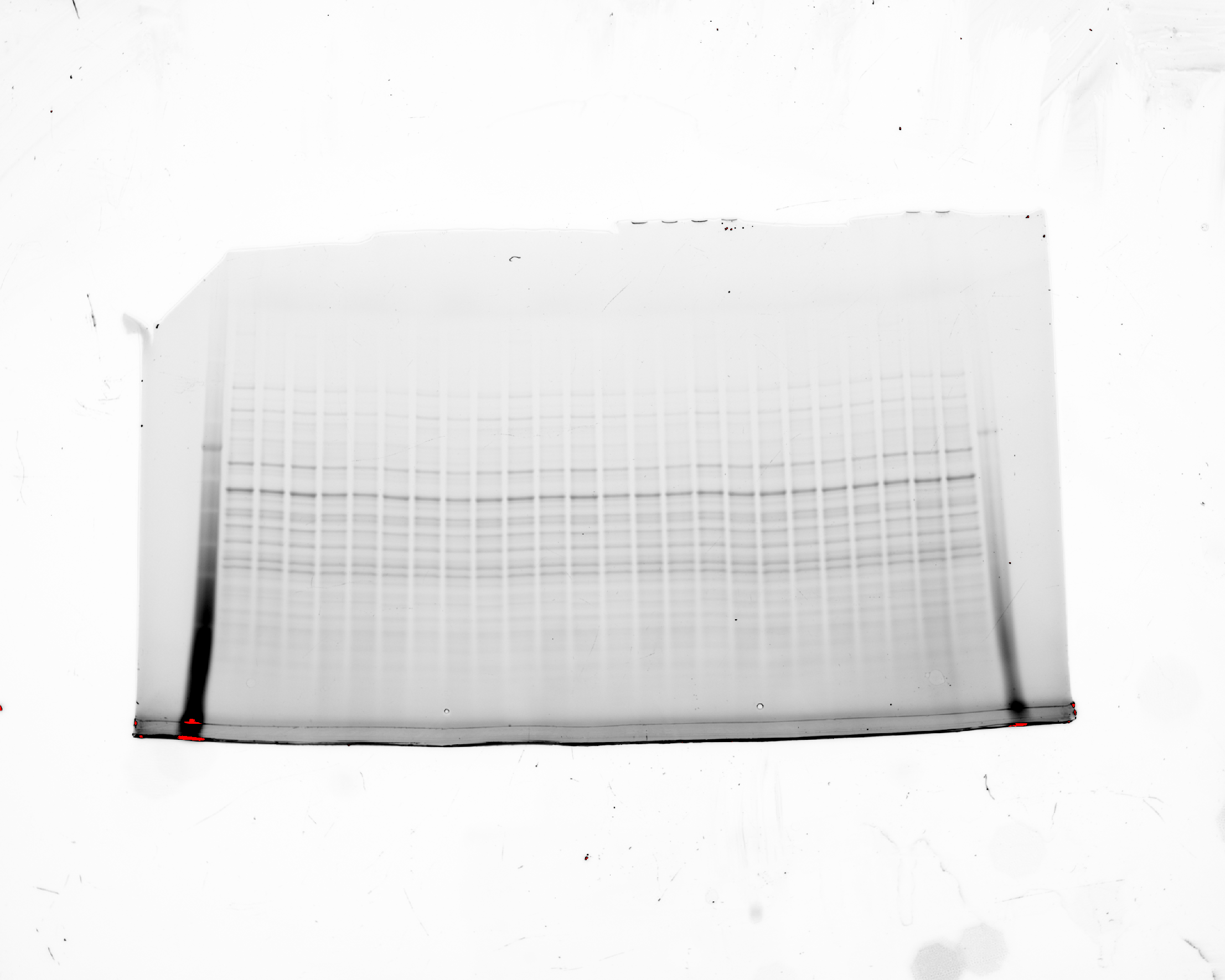

Supplement: Figure 2—source data 2. [file elife-89606-fig2-data2.zip › Figure 2-source data 2/BaldridgeLab 2023-04-27 09h06m32s Stain Free Gel 14.553s(Stain Free Gel).tif]

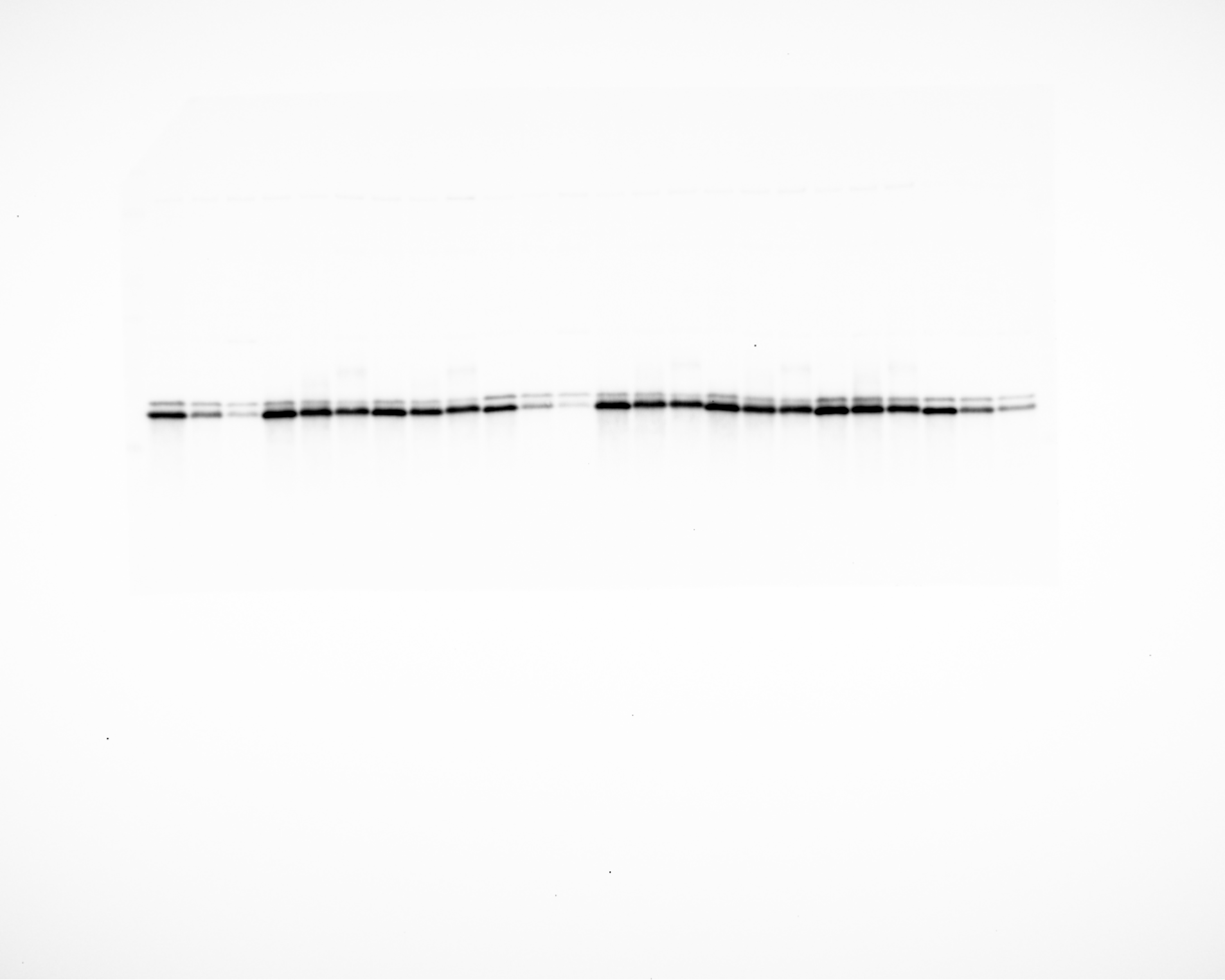

Supplement: Figure 2—source data 2. [file elife-89606-fig2-data2.zip › Figure 2-source data 2/BaldridgeLab 2023-04-27 13h53m42s DyLight 800 30.000s(DyLight 800).tif]

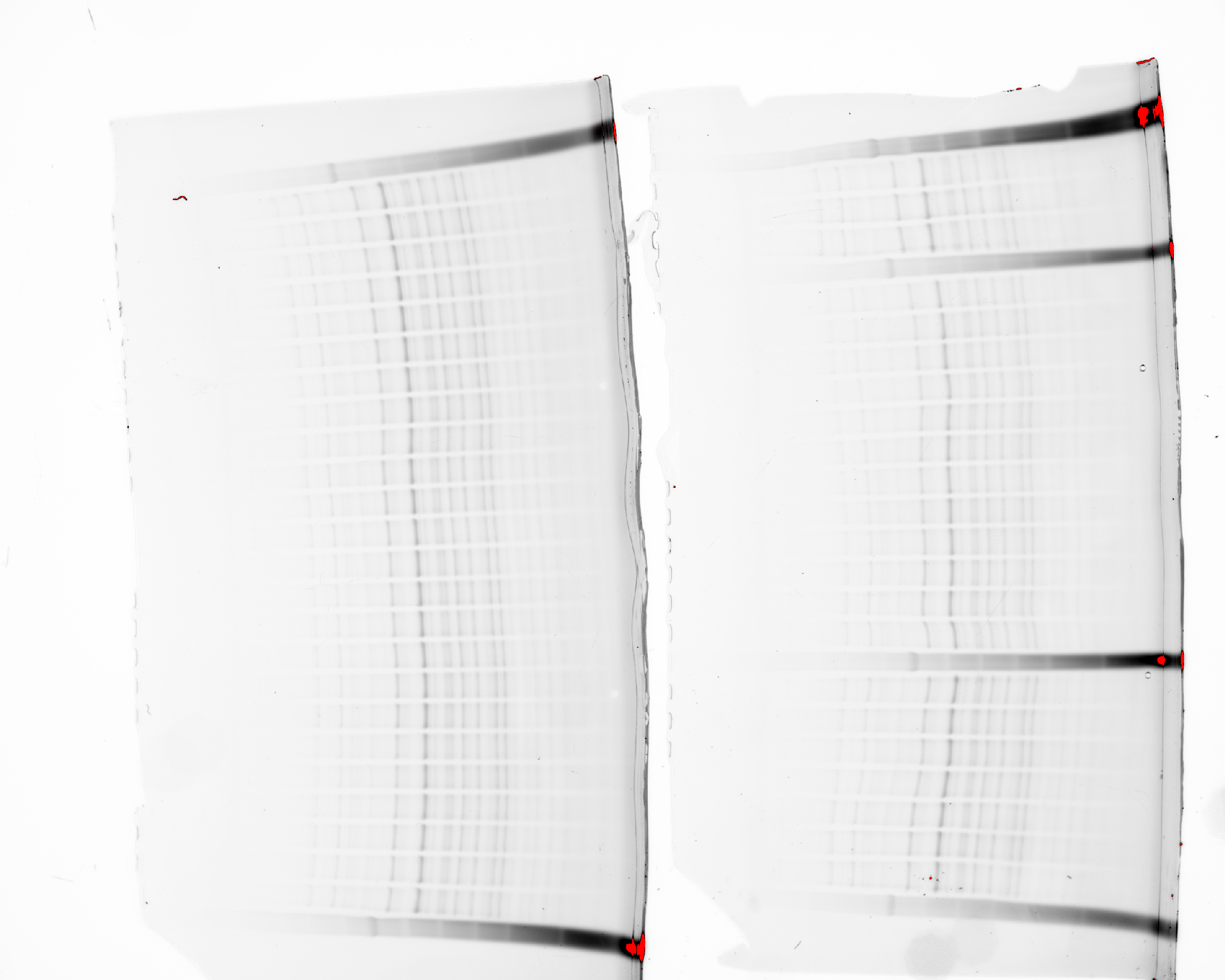

Supplement: Figure 2—source data 2. [file elife-89606-fig2-data2.zip › Figure 2-source data 2/BaldridgeLab 2023-05-01 09h16m57s Stain Free Gel 12.203s(Stain Free Gel).tif]

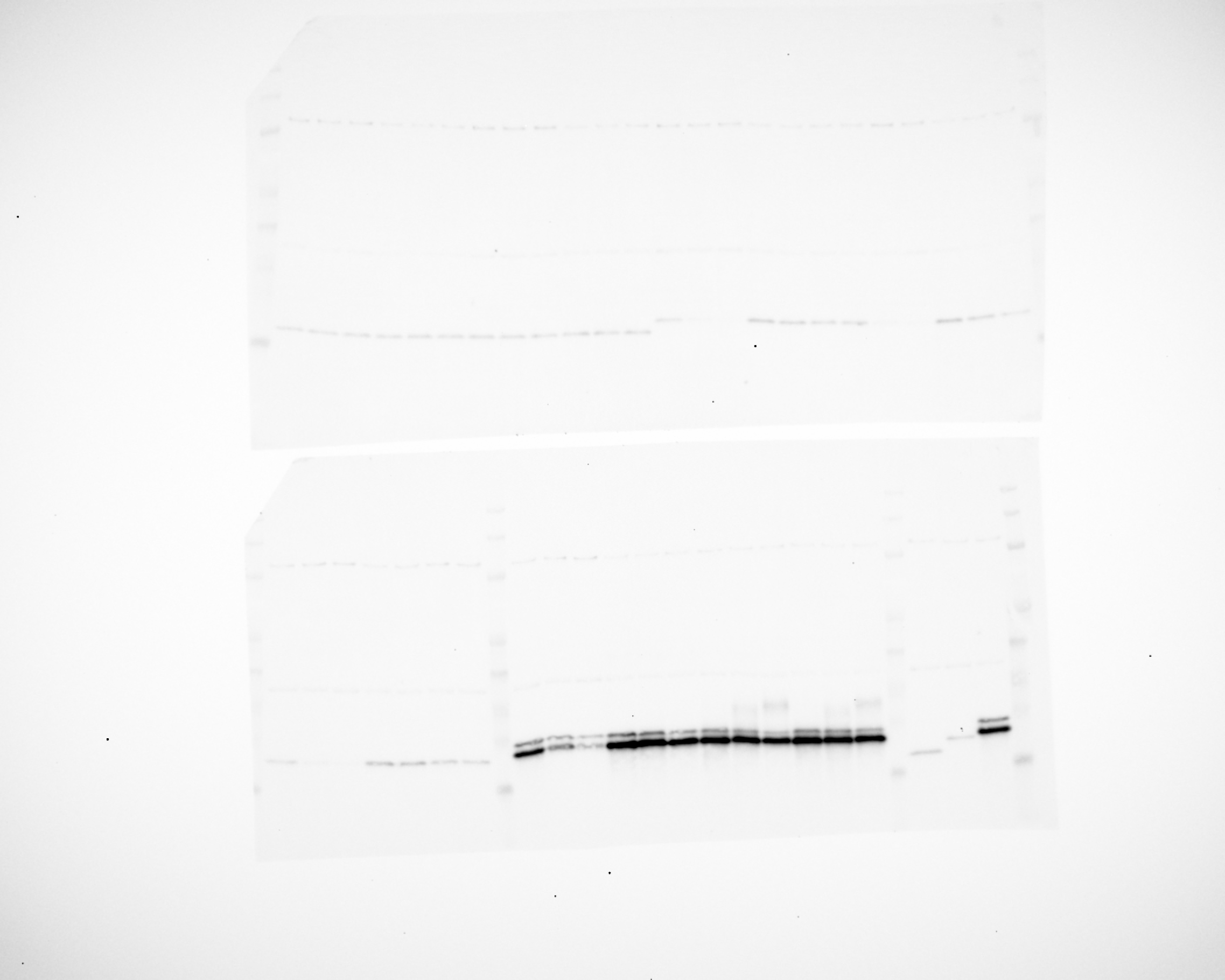

Supplement: Figure 2—source data 2. [file elife-89606-fig2-data2.zip › Figure 2-source data 2/BaldridgeLab 2023-05-01 13h25m15s DyLight 800 60.000s(DyLight 800).tif]

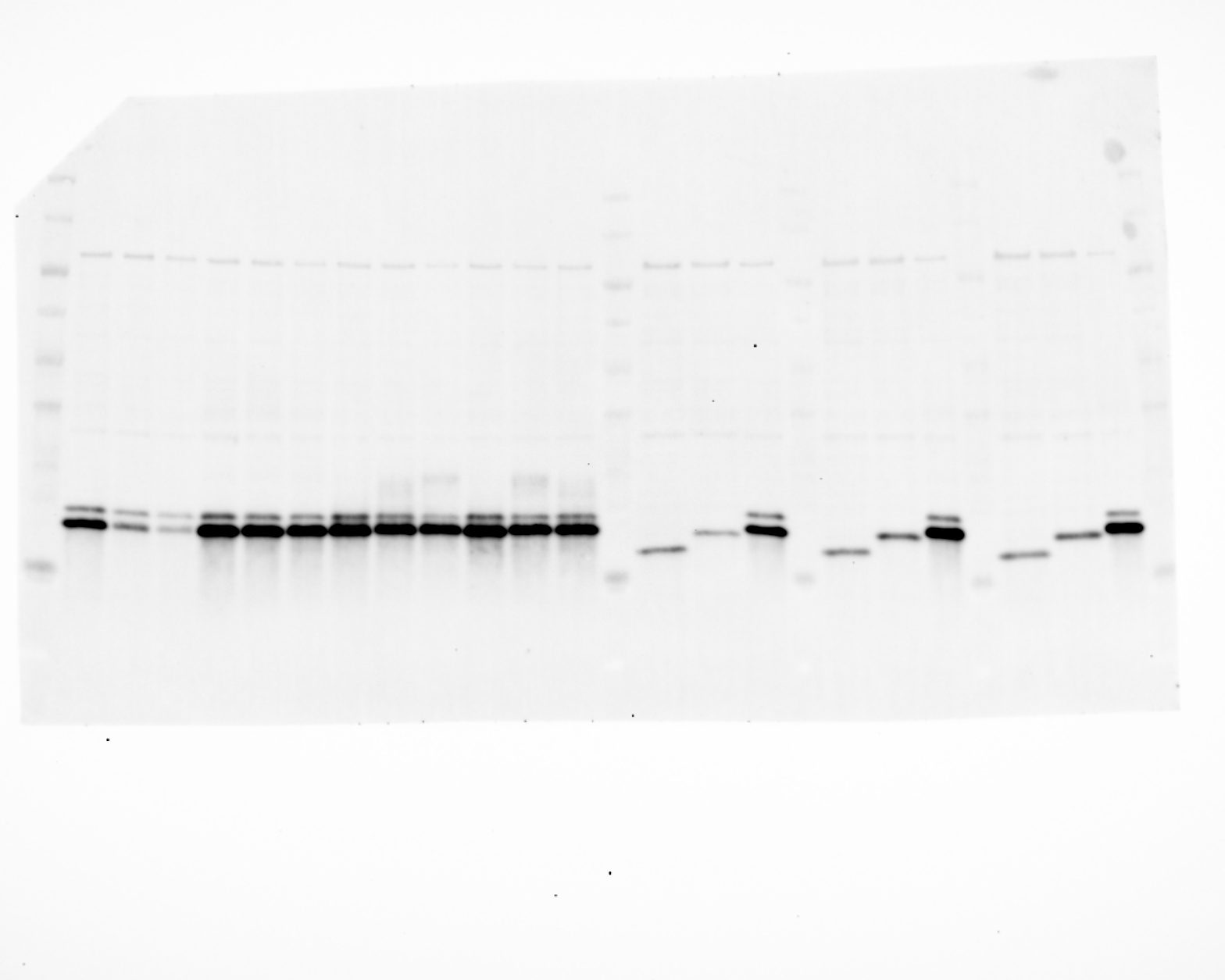

Supplement: Figure 2—source data 2. [file elife-89606-fig2-data2.zip › Figure 2-source data 2/BaldridgeLab 2023-05-02 13h45m41s DyLight 800 50.000s(DyLight 800).tif]

Figure 2-figure supplement 1

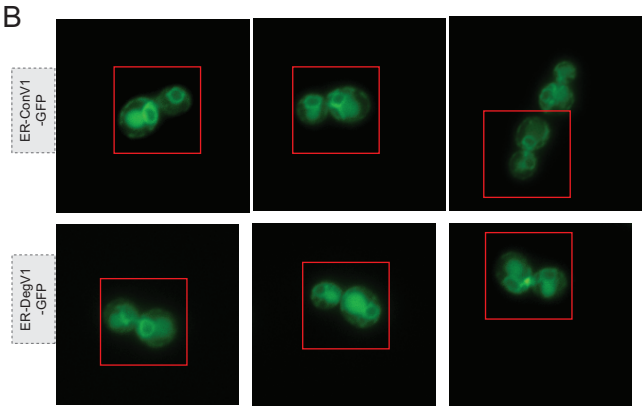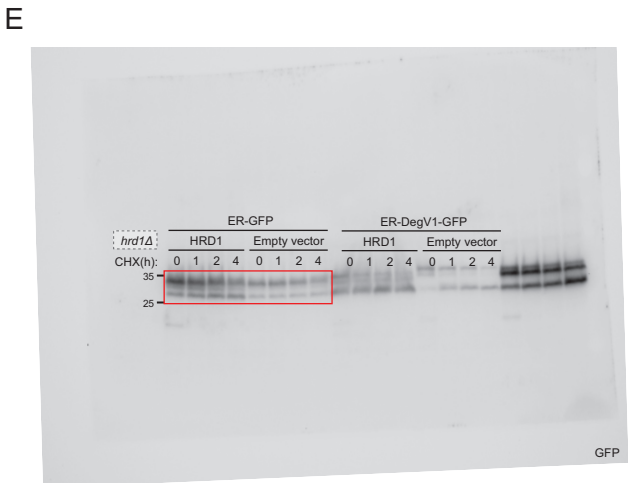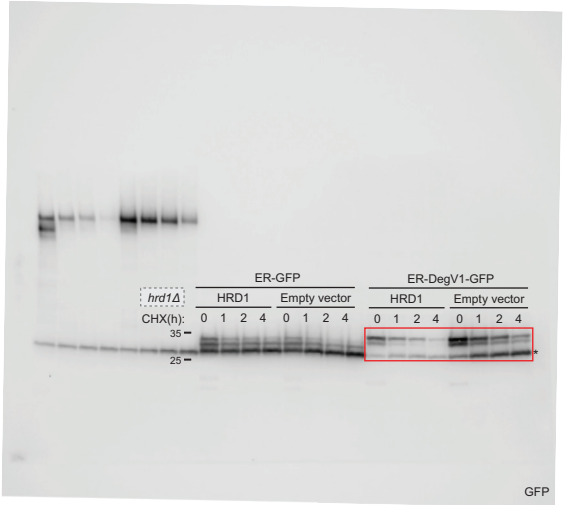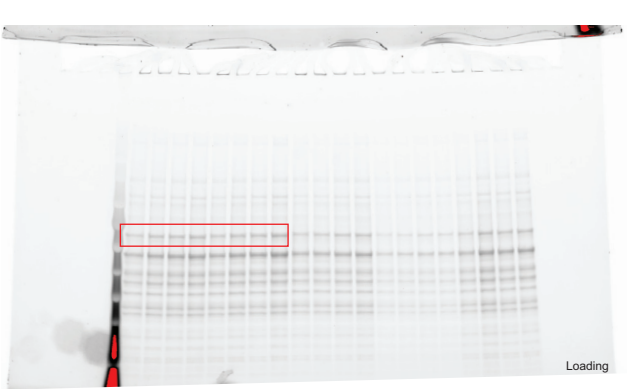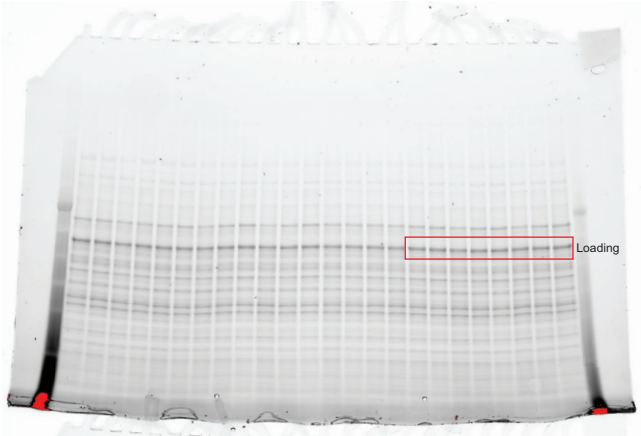

Figure 2-figure supplement 1

F

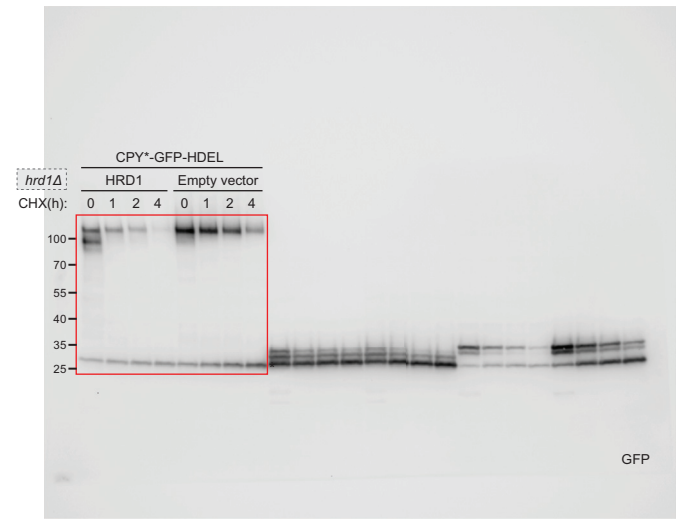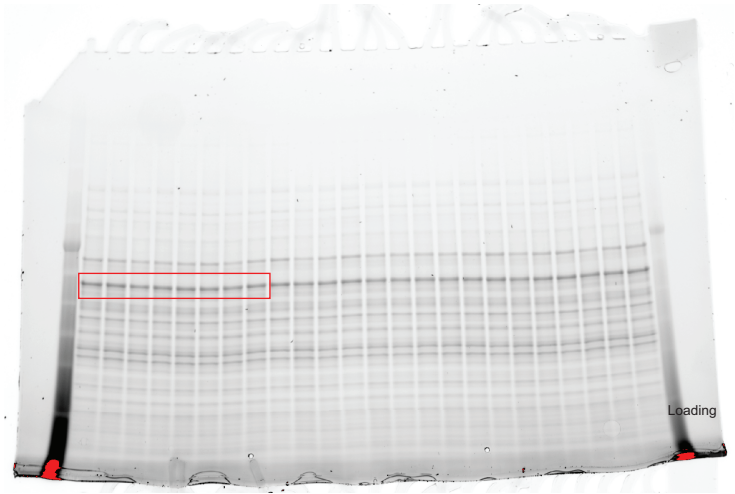

Figure 2-figure supplement 1

G

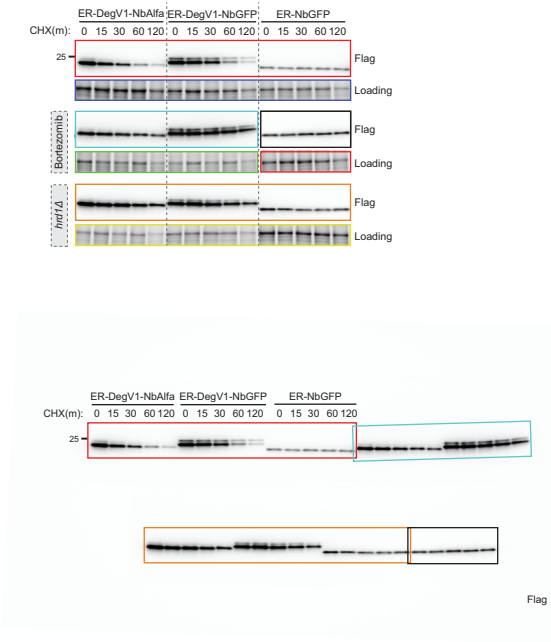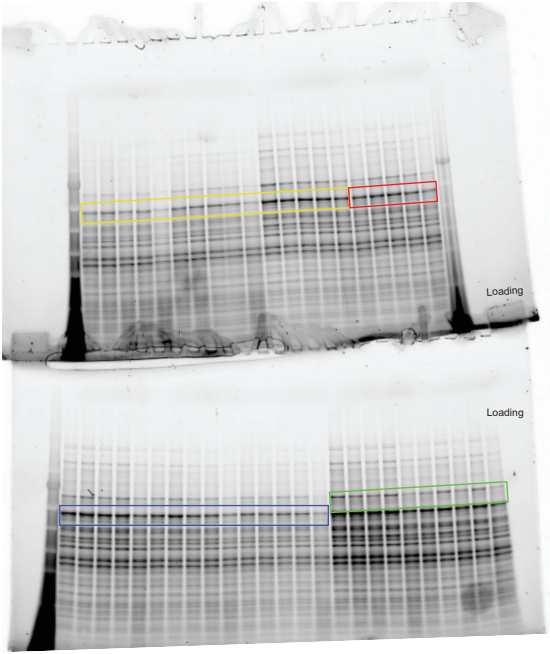

Supplement: Figure 2—figure supplement 1—source data 1. [file elife-89606-fig2-figsupp1-data1.zip › Figure 2-figure supplement 1-source data 1.pdf]

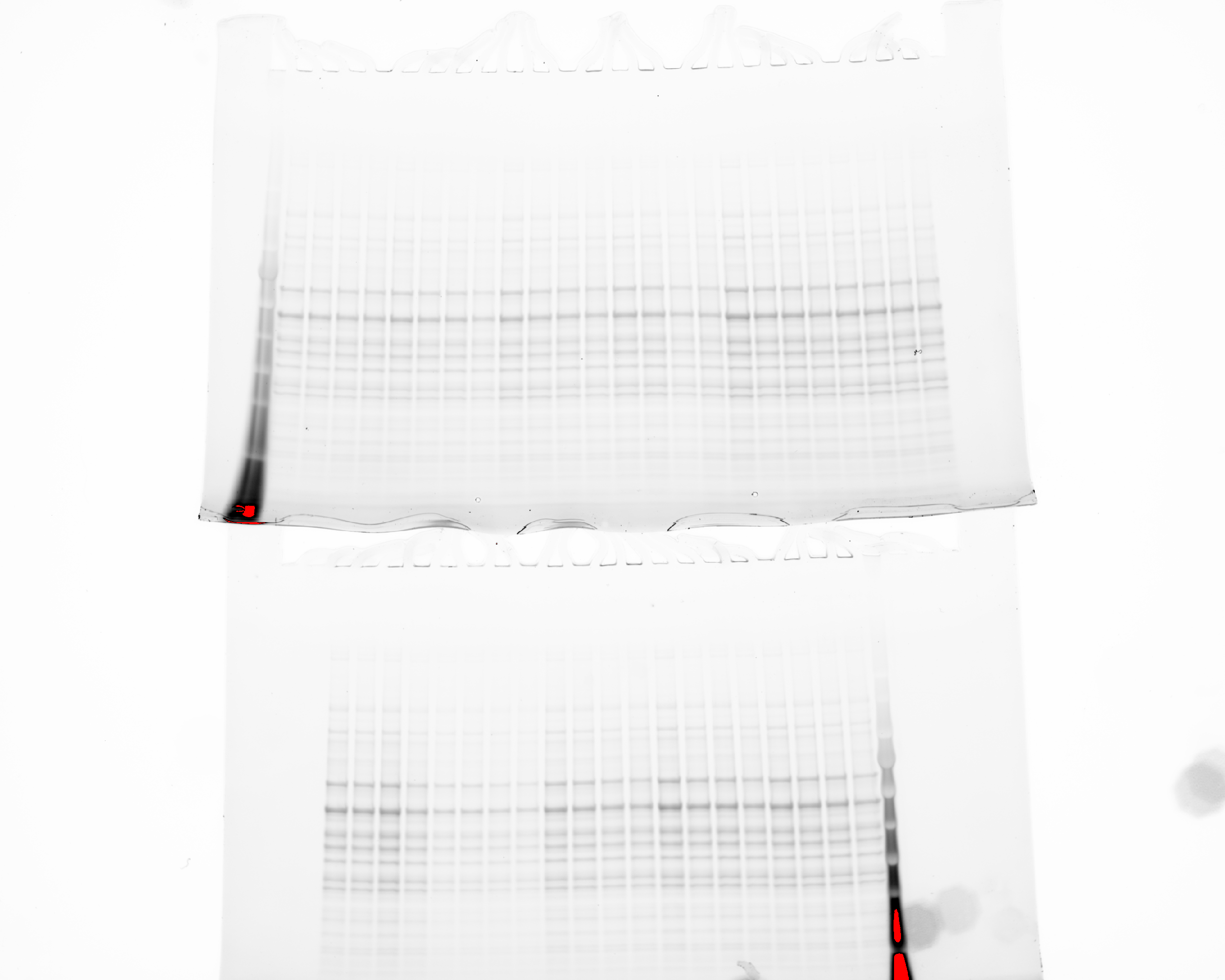

Supplement: Figure 2—figure supplement 1—source data 2. [file elife-89606-fig2-figsupp1-data2.zip › Figure 2-figure supplement 1-source data 2/BaldridgeLab 2020-12-04 11h08m25s Stain Free Gel 9.179s(Stain Free Gel).tif]

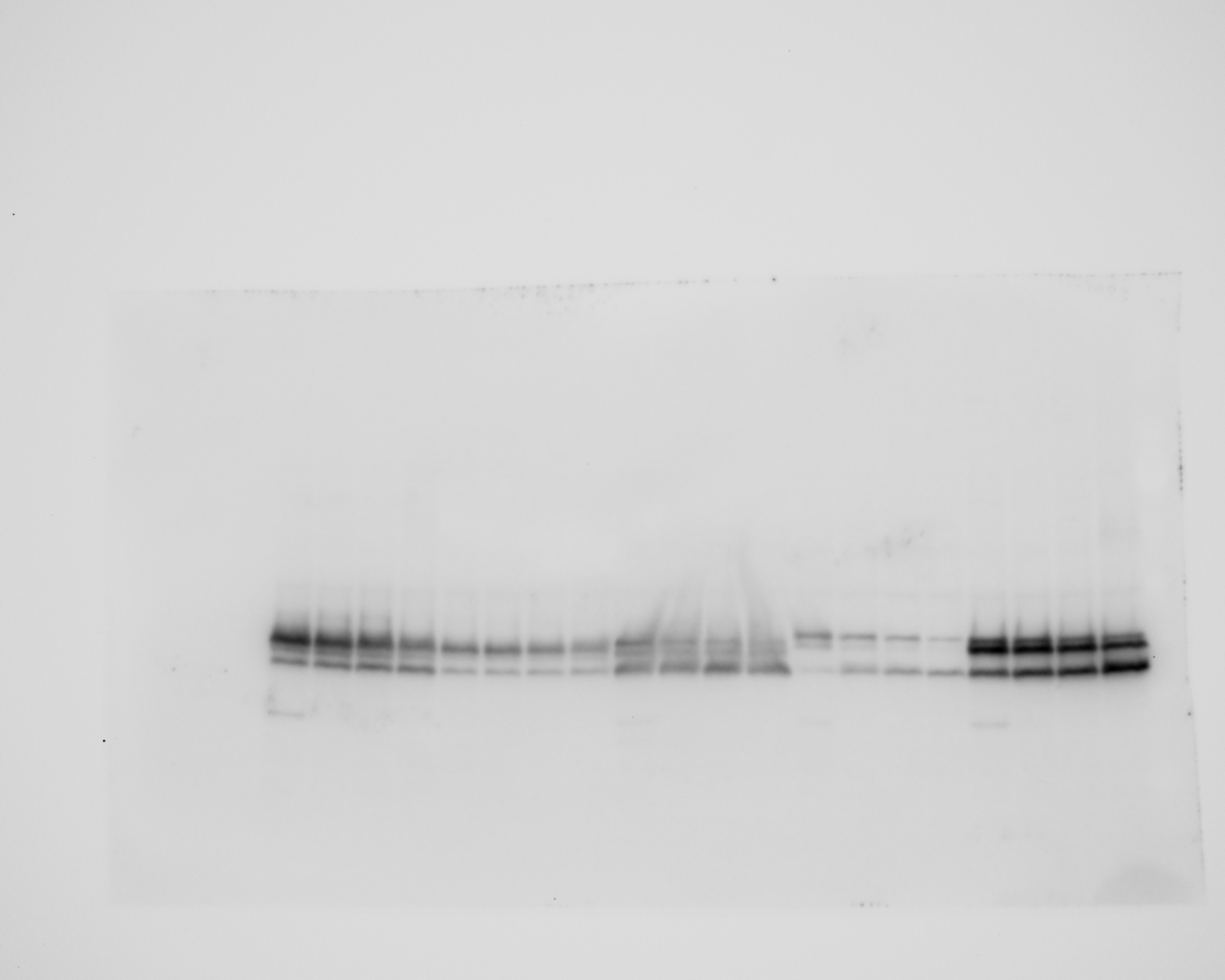

Supplement: Figure 2—figure supplement 1—source data 2. [file elife-89606-fig2-figsupp1-data2.zip › Figure 2-figure supplement 1-source data 2/BaldridgeLab 2020-12-04 17h25m27s Chemiluminescence 60.000s(Chemiluminescence).tif]

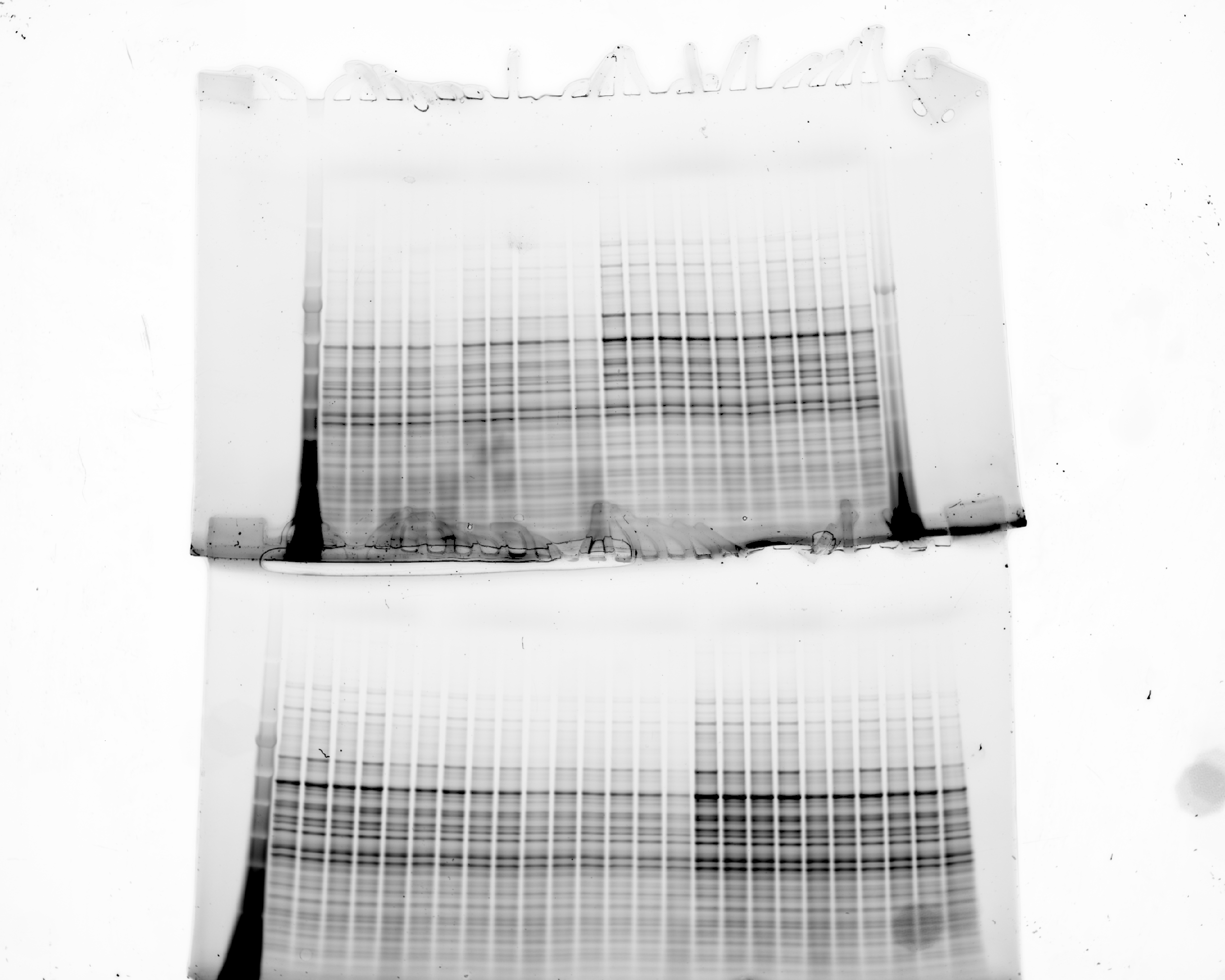

Supplement: Figure 2—figure supplement 1—source data 2. [file elife-89606-fig2-figsupp1-data2.zip › Figure 2-figure supplement 1-source data 2/BaldridgeLab 2021-10-13 11h43m42s Stain Free Gel 25.000s(Stain Free Gel).tif]

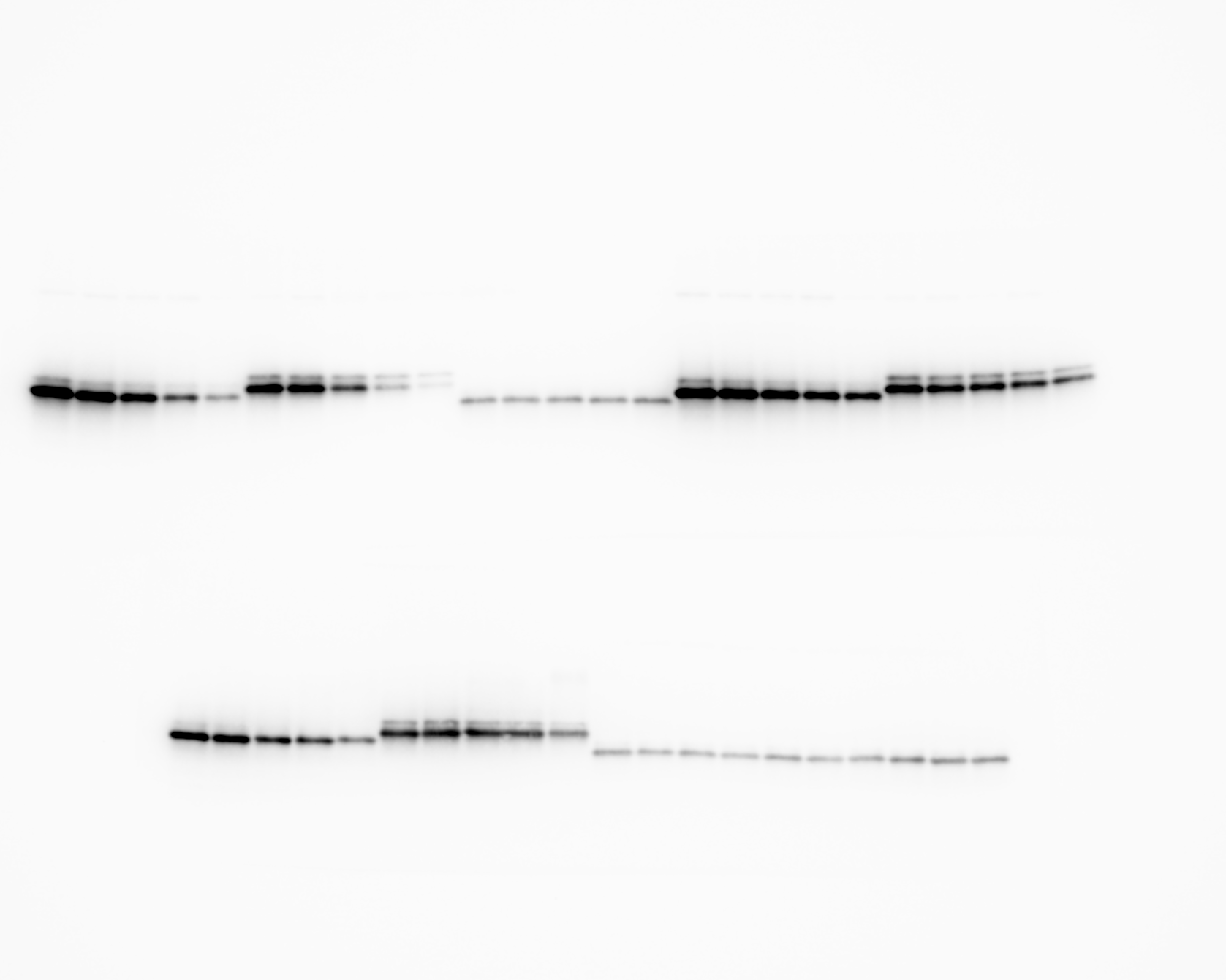

Supplement: Figure 2—figure supplement 1—source data 2. [file elife-89606-fig2-figsupp1-data2.zip › Figure 2-figure supplement 1-source data 2/BaldridgeLab 2021-10-13 18h01m38s Chemiluminescence 2.000s(Chemiluminescence).tif]

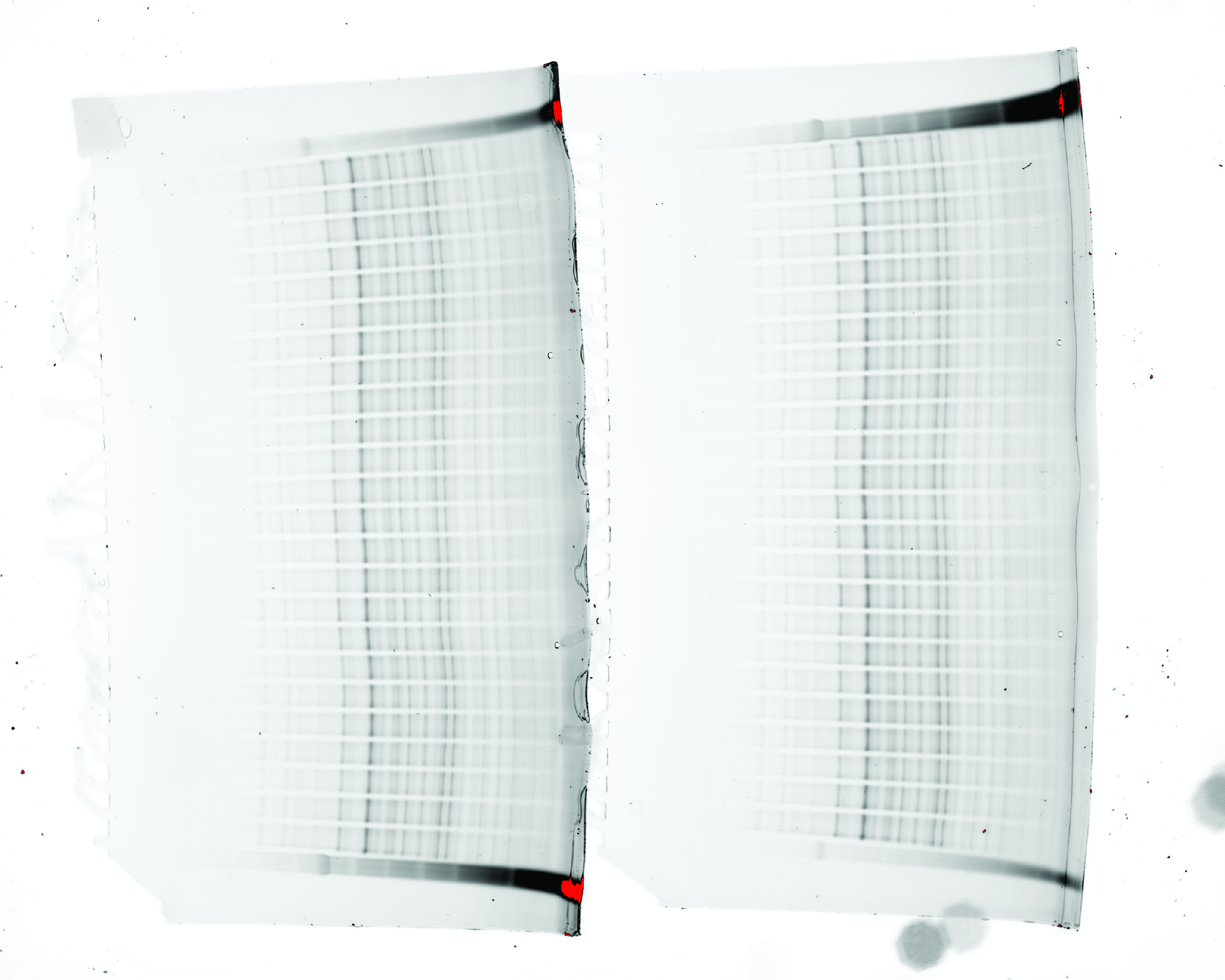

Supplement: Figure 2—figure supplement 1—source data 2. [file elife-89606-fig2-figsupp1-data2.zip › Figure 2-figure supplement 1-source data 2/C_20.12.04a_StainFree_GNRWG_bottom.tif]

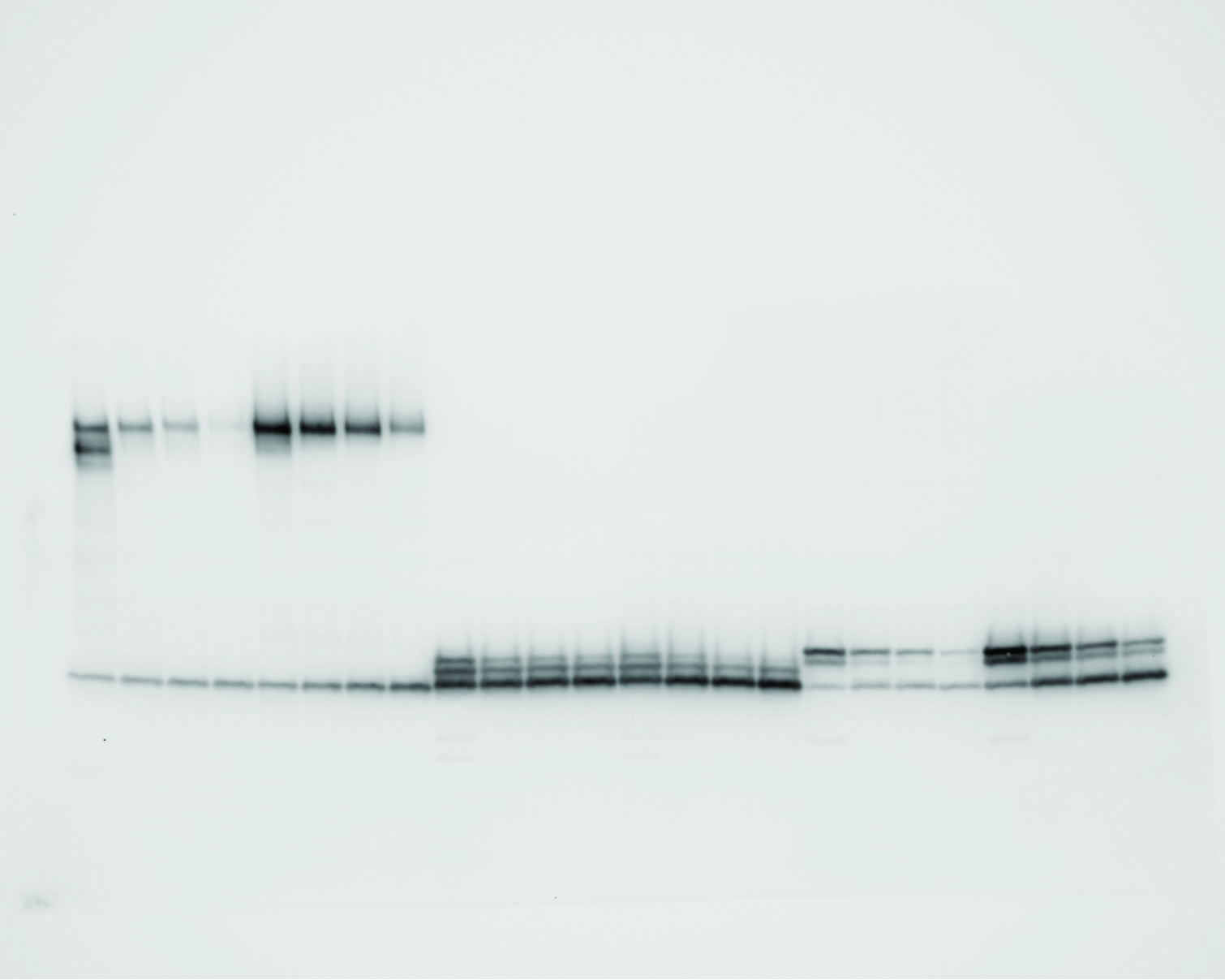

Supplement: Figure 2—figure supplement 1—source data 2. [file elife-89606-fig2-figsupp1-data2.zip › Figure 2-figure supplement 1-source data 2/C_20.12.04_antiGFP_GFPdegron_26s.tif]

Figure 2-figure supplement 2

A

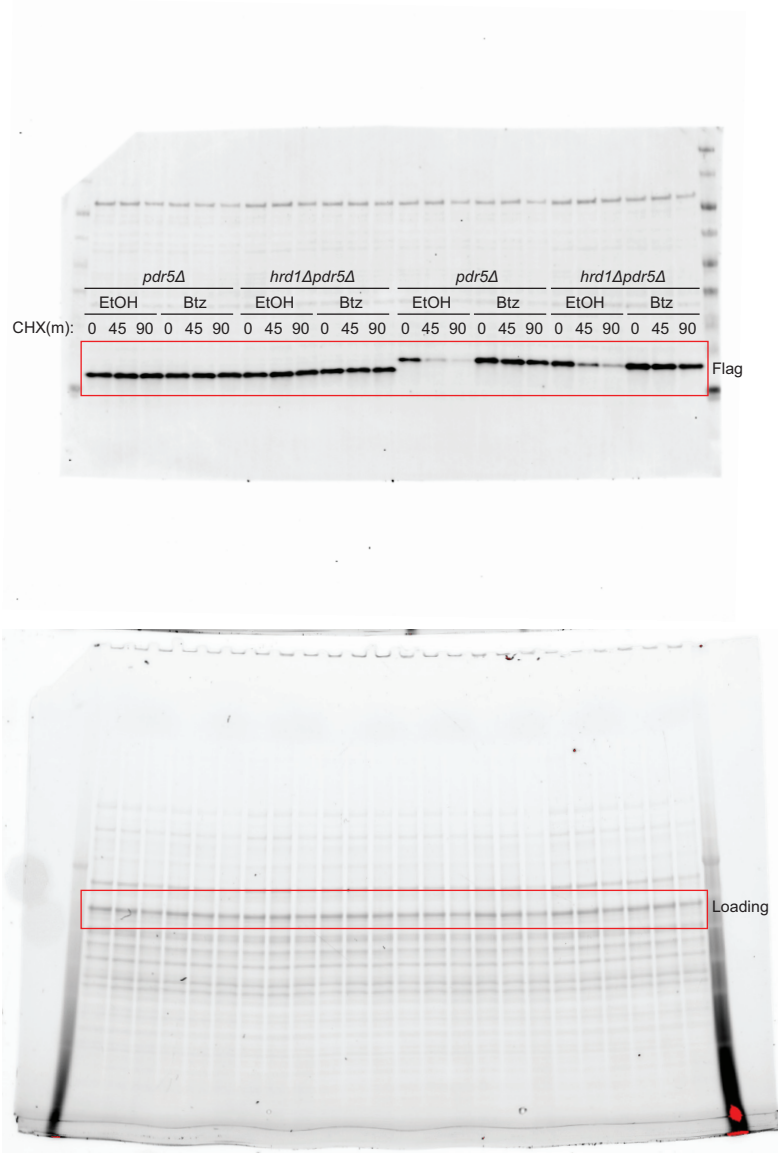

Supplement: Figure 2—figure supplement 2—source data 1. [file elife-89606-fig2-figsupp2-data1.zip › Figure 2-figure supplement 2-source data 1.pdf]

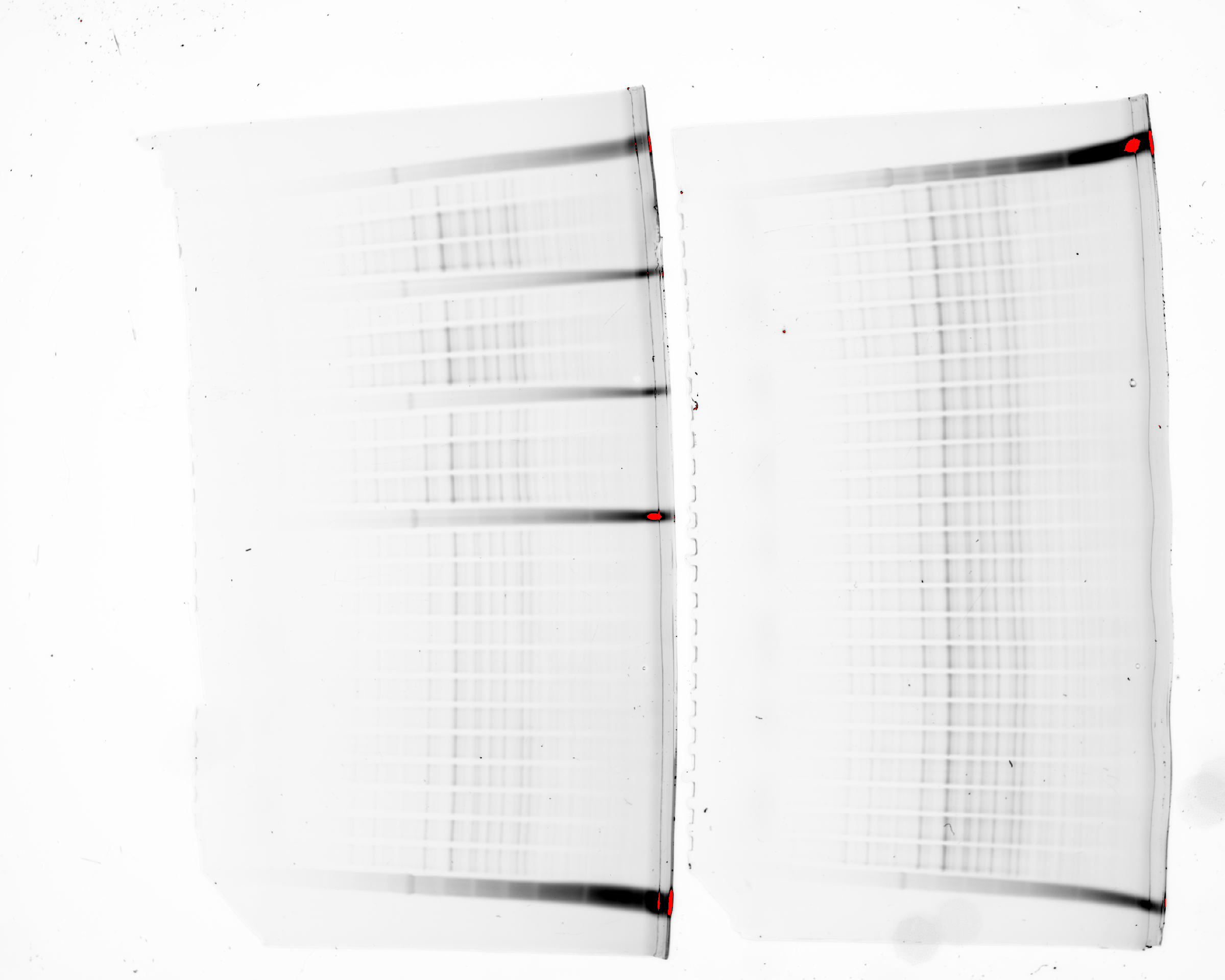

Supplement: Figure 2—figure supplement 2—source data 2. [file elife-89606-fig2-figsupp2-data2.zip › Figure 2-figure supplement 2-source data 2/BaldridgeLab 2023-05-02 10h47m47s Stain Free Gel 10.598s(Stain Free Gel).tif]

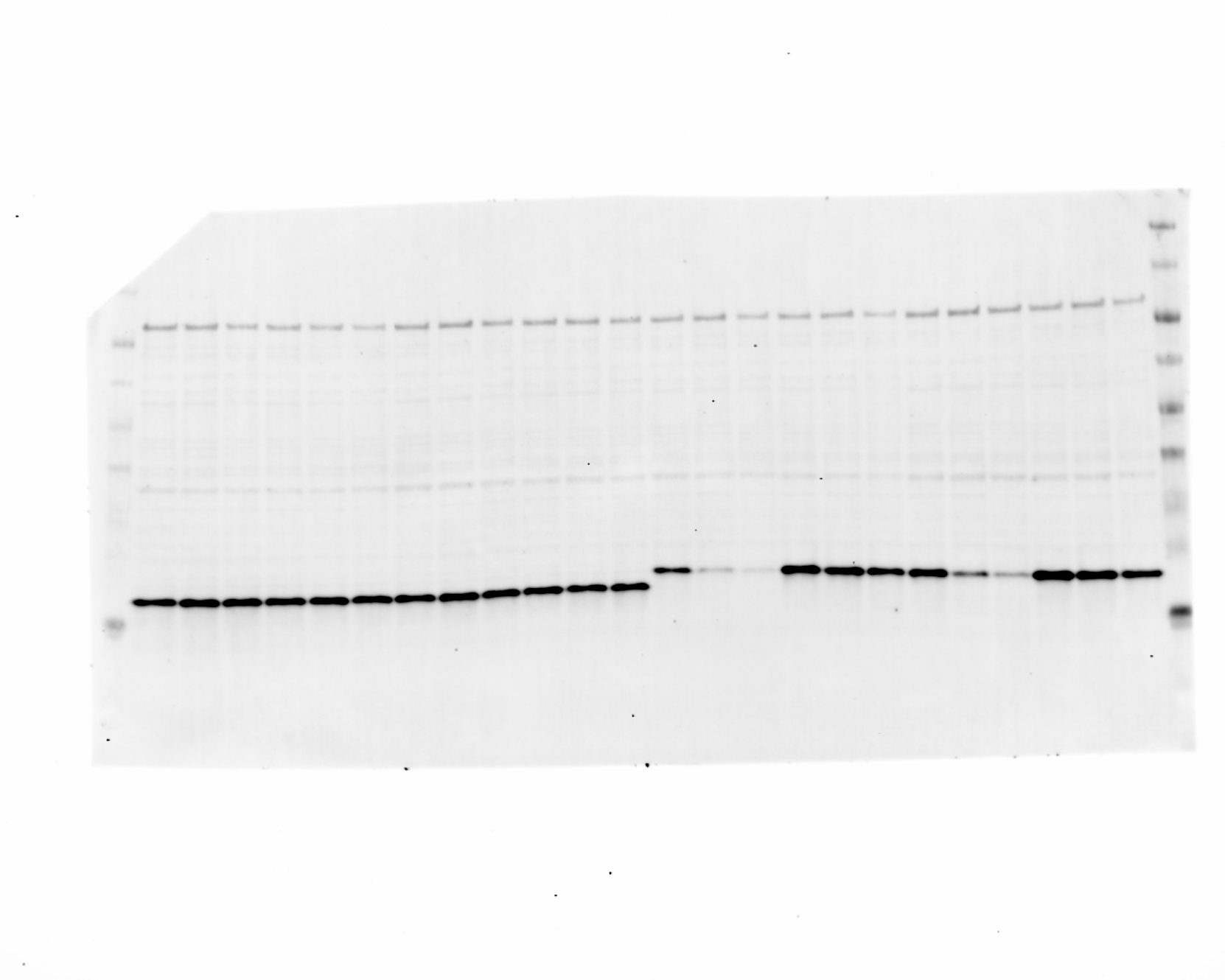

Supplement: Figure 2—figure supplement 2—source data 2. [file elife-89606-fig2-figsupp2-data2.zip › Figure 2-figure supplement 2-source data 2/BaldridgeLab 2023-05-02 13h54m14s DyLight 800 300.000s(DyLight 800).tif]

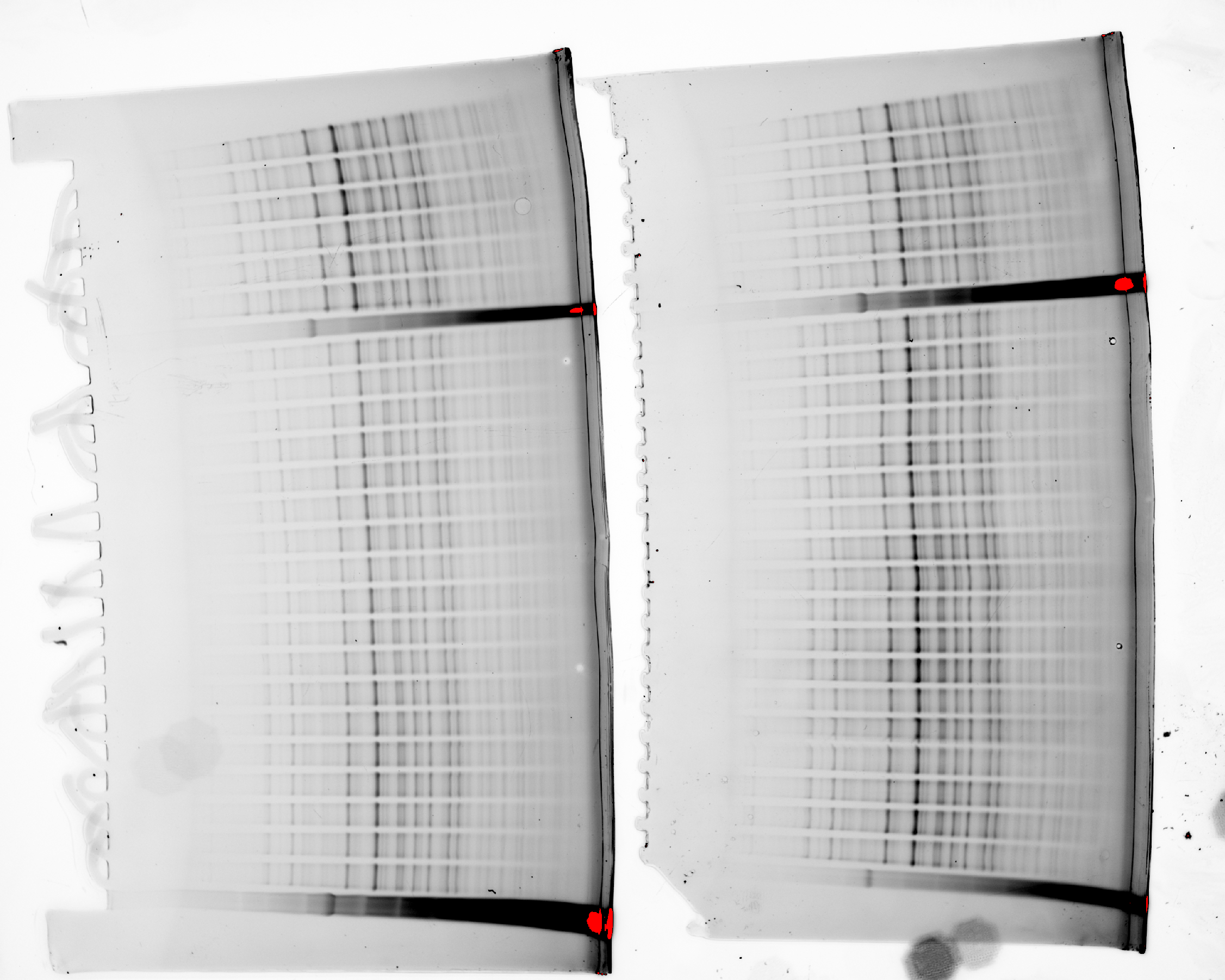

Supplement: Figure 3—source data 2. [file elife-89606-fig3-data2.zip › Figure 3-source data 2/BaldridgeLab 2022-01-16 10h33m45s Stain Free Gel 13.748s(Stain Free Gel).tif]

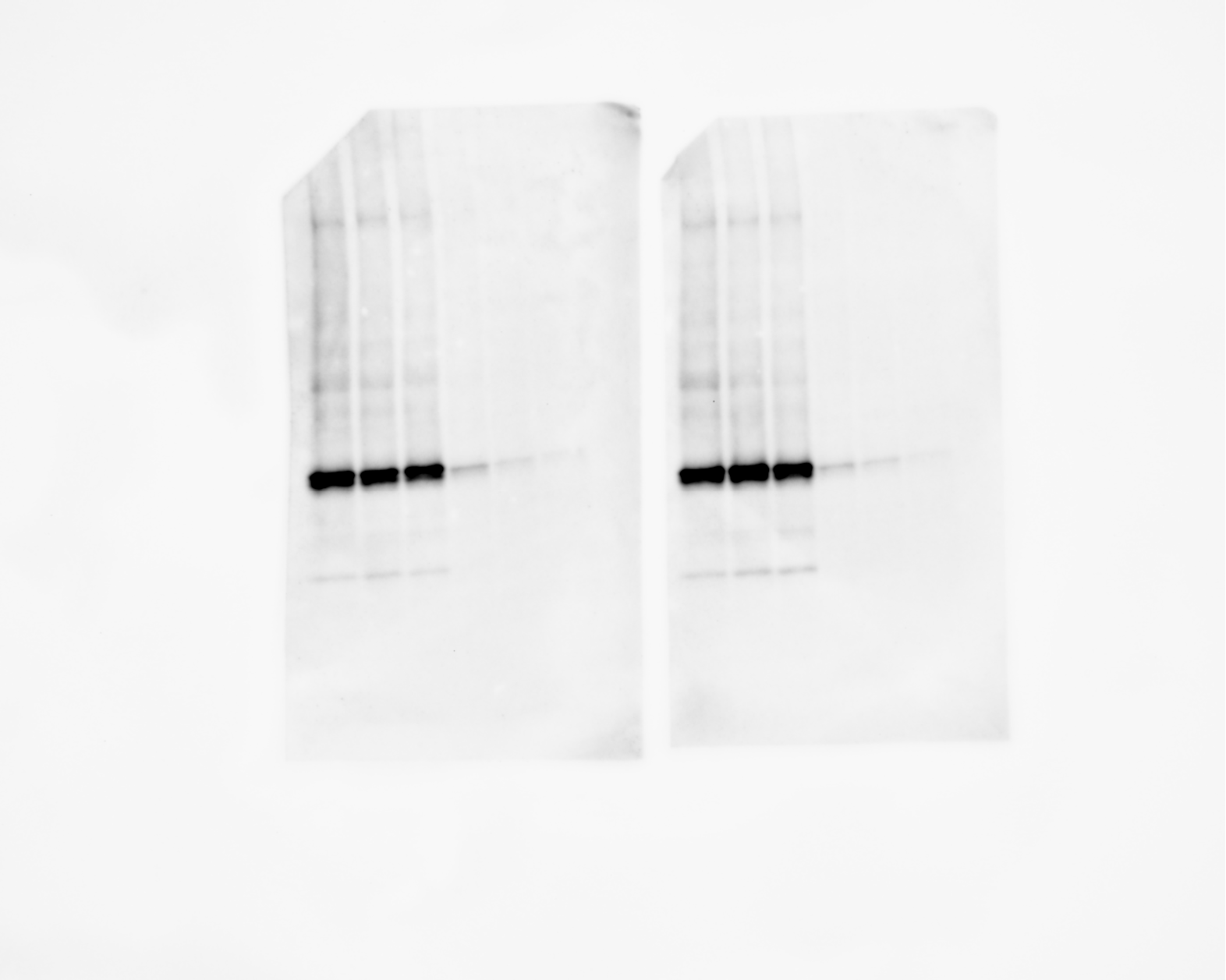

Supplement: Figure 3—source data 2. [file elife-89606-fig3-data2.zip › Figure 3-source data 2/BaldridgeLab 2022-01-17 11h05m17s Chemiluminescence 90.000s(Chemiluminescence).tif]

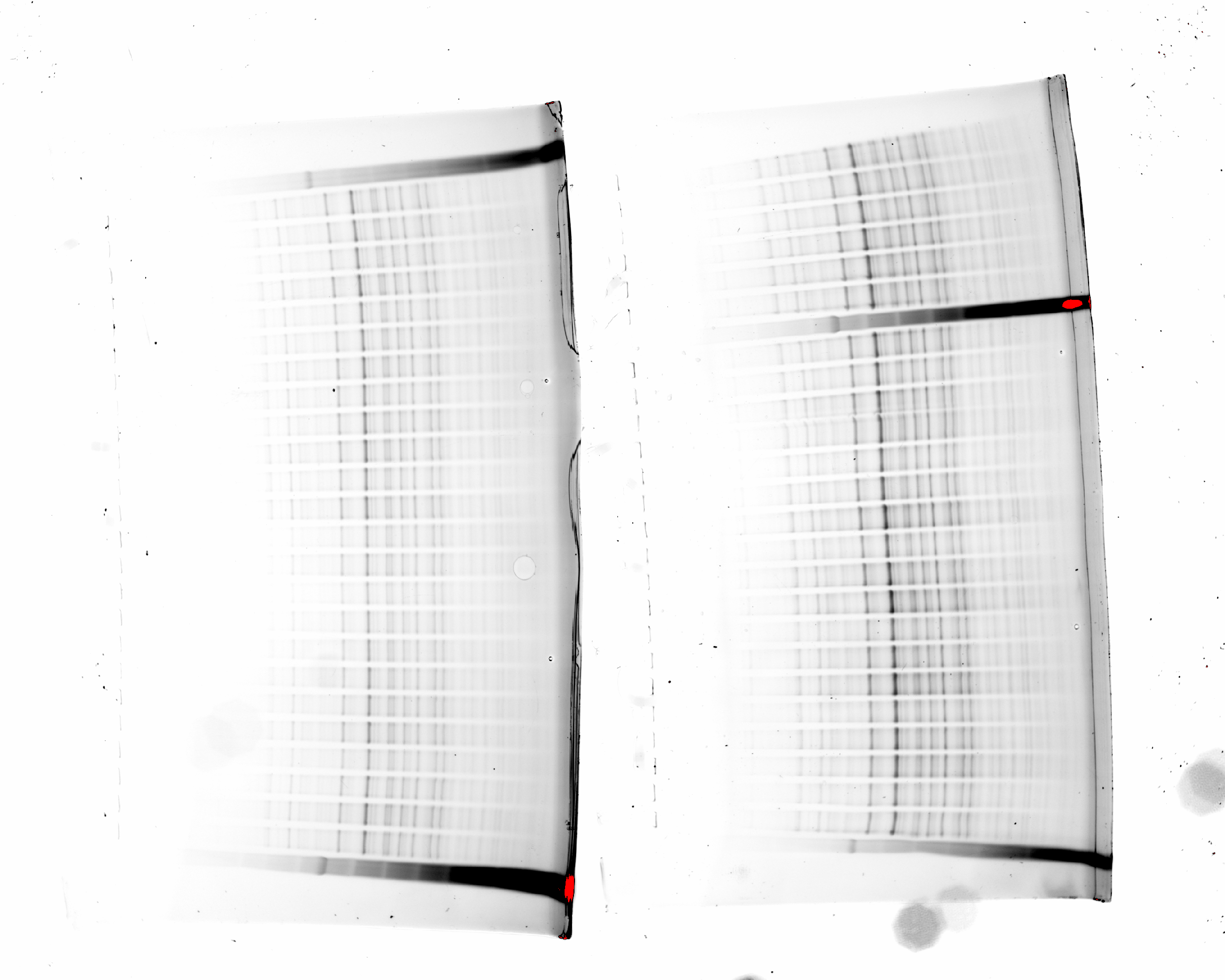

Supplement: Figure 3—source data 2. [file elife-89606-fig3-data2.zip › Figure 3-source data 2/BaldridgeLab 2022-01-19 10h03m25s Stain Free Gel 12.085s(Stain Free Gel).tif]

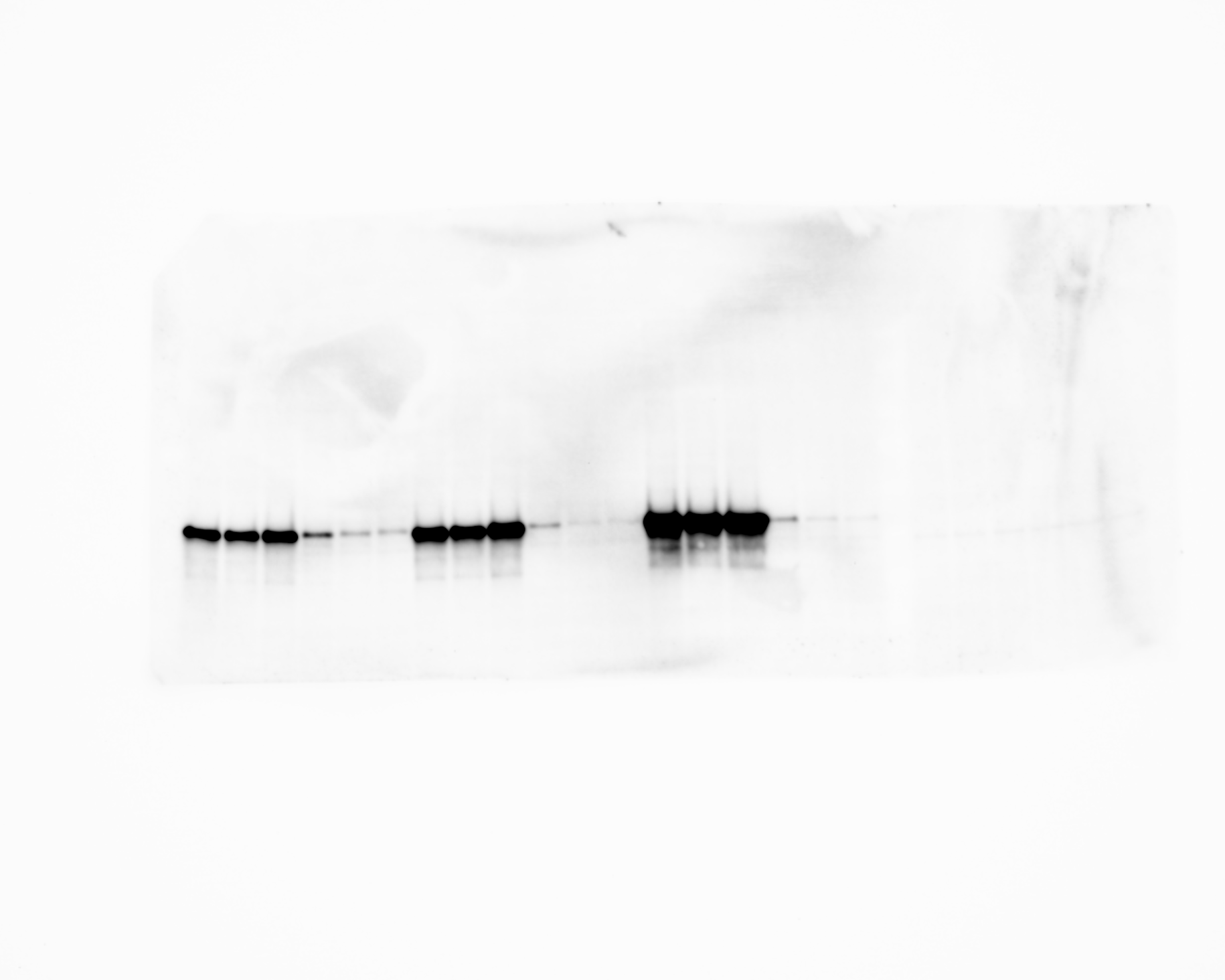

Supplement: Figure 3—source data 2. [file elife-89606-fig3-data2.zip › Figure 3-source data 2/BaldridgeLab 2022-01-19 13h34m04s Chemiluminescence 38.000s(Chemiluminescence).tif]

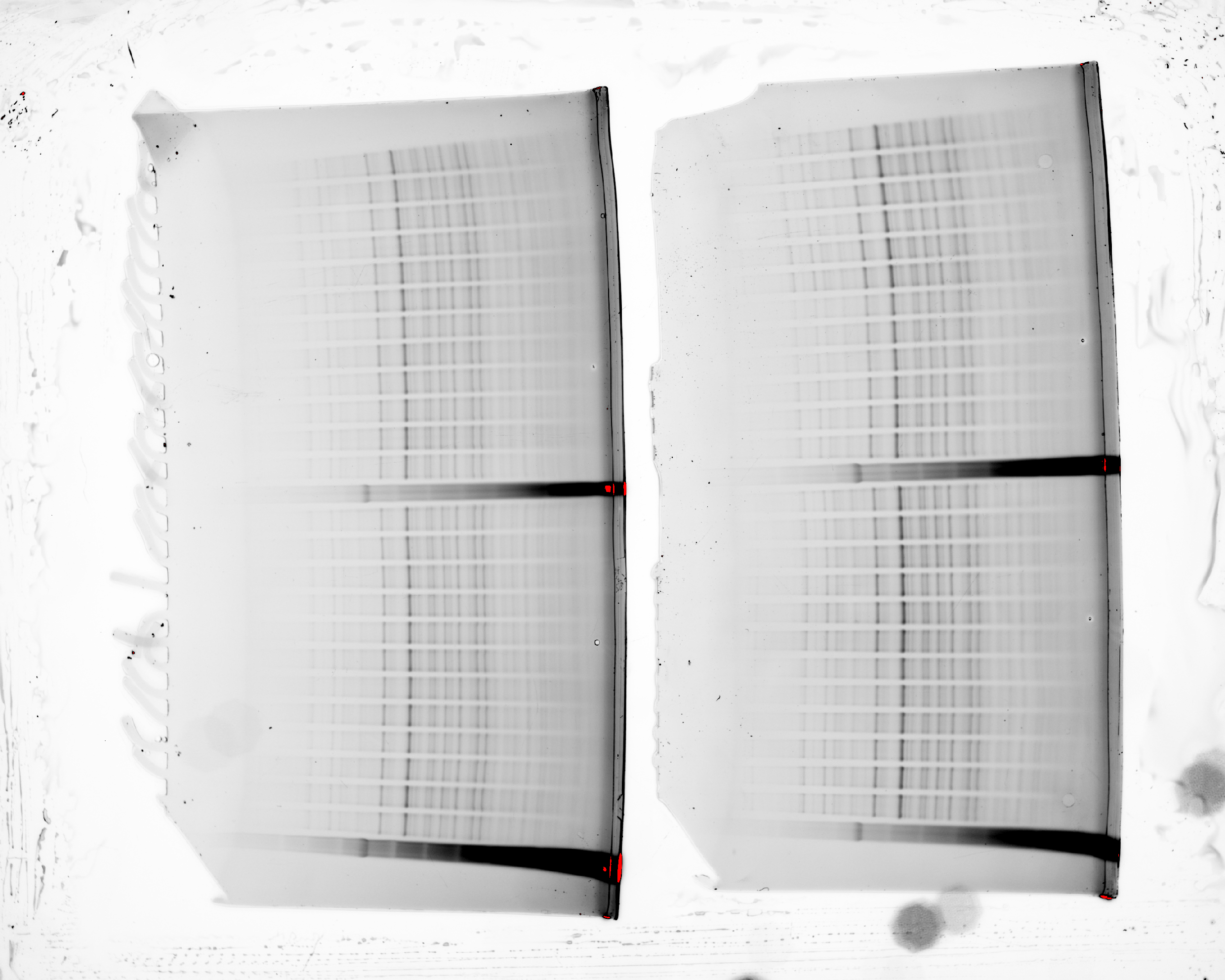

Supplement: Figure 3—source data 2. [file elife-89606-fig3-data2.zip › Figure 3-source data 2/BaldridgeLab 2022-01-30 10h35m09s Stain Free Gel 12.955s(Stain Free Gel).tif]

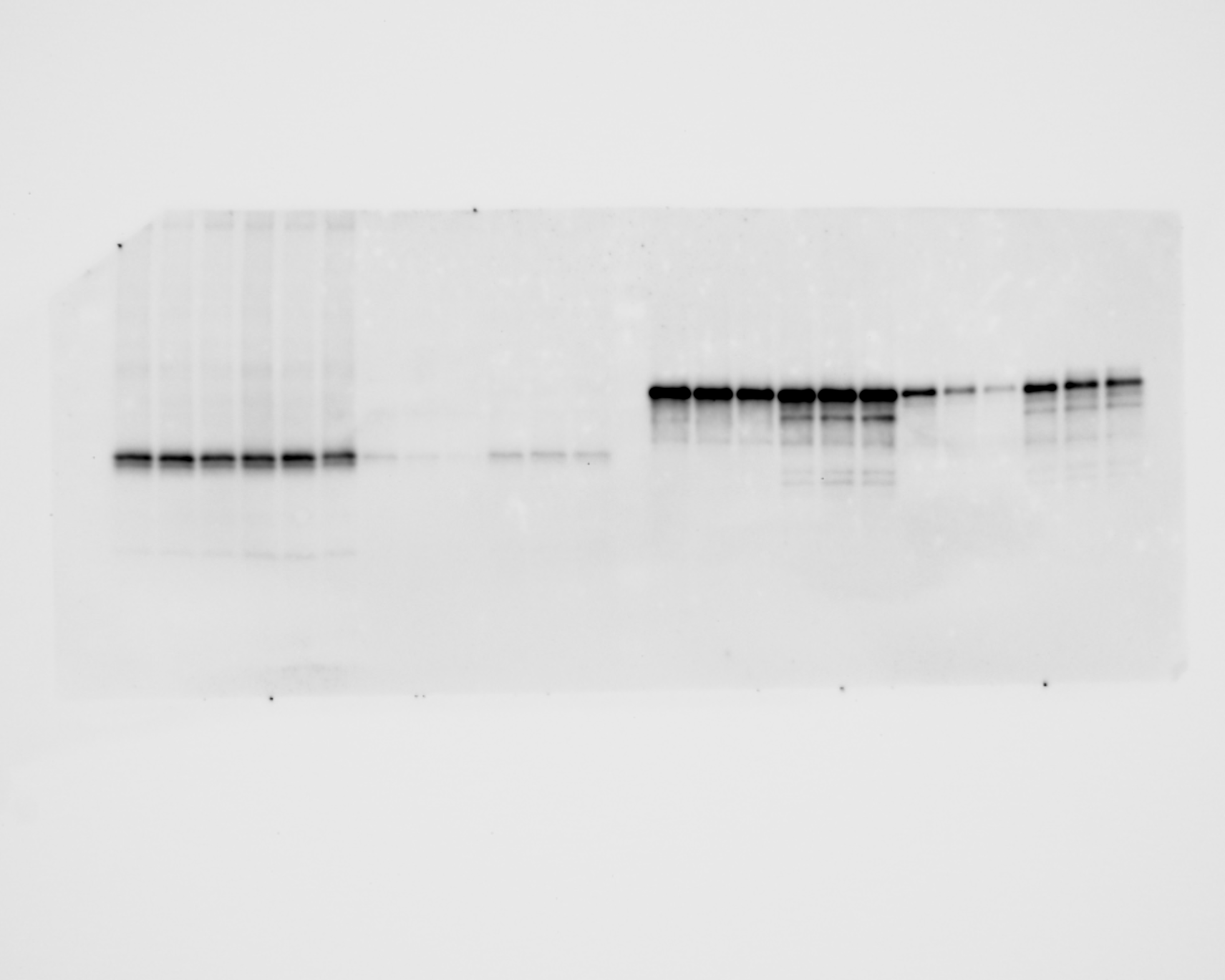

Supplement: Figure 3—source data 2. [file elife-89606-fig3-data2.zip › Figure 3-source data 2/BaldridgeLab 2022-01-31 11h16m59s Chemiluminescence 60.000s(Chemiluminescence) (2).tif]

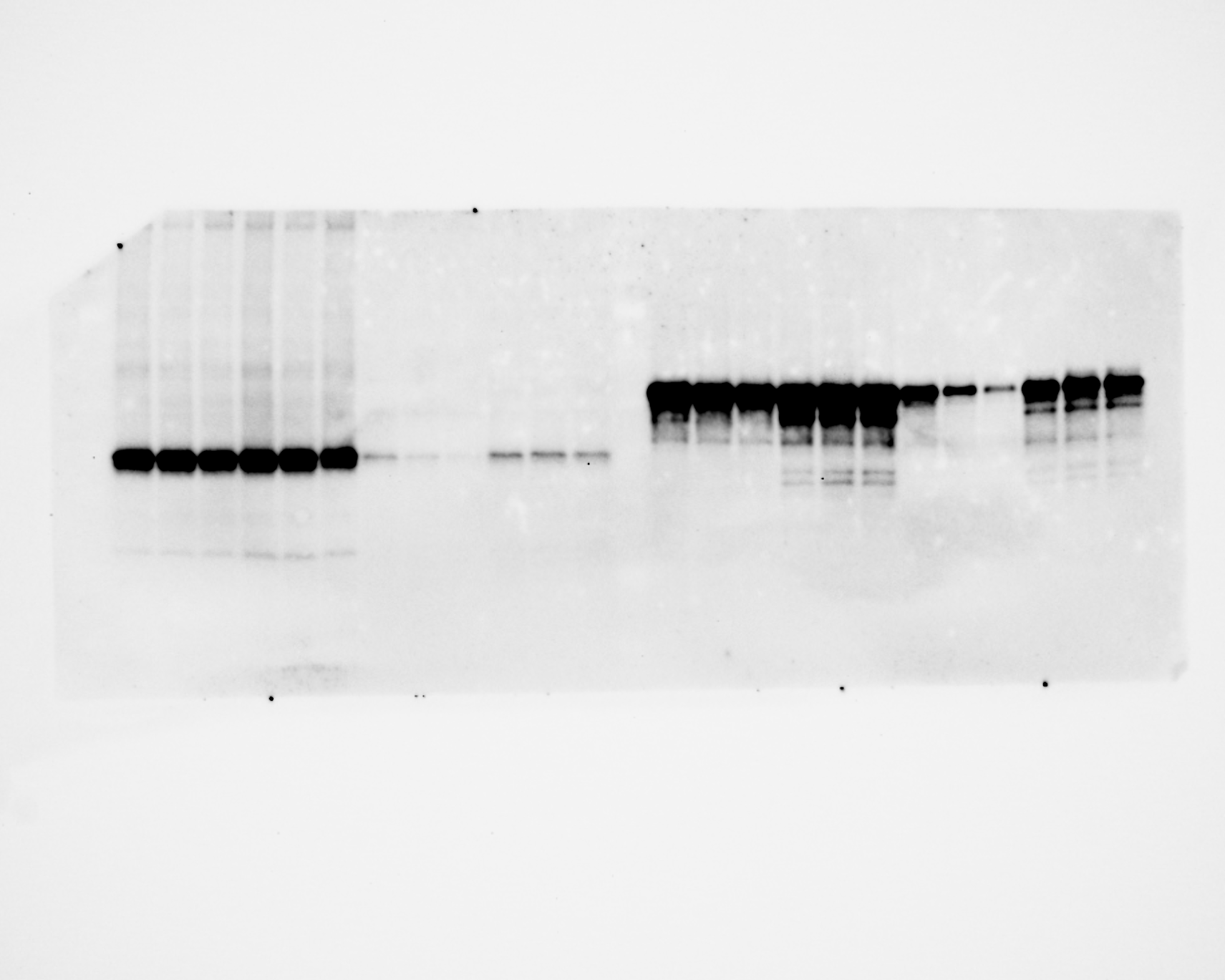

Supplement: Figure 3—source data 2. [file elife-89606-fig3-data2.zip › Figure 3-source data 2/BaldridgeLab 2022-01-31 11h16m59s Chemiluminescence 60.000s(Chemiluminescence).tif]

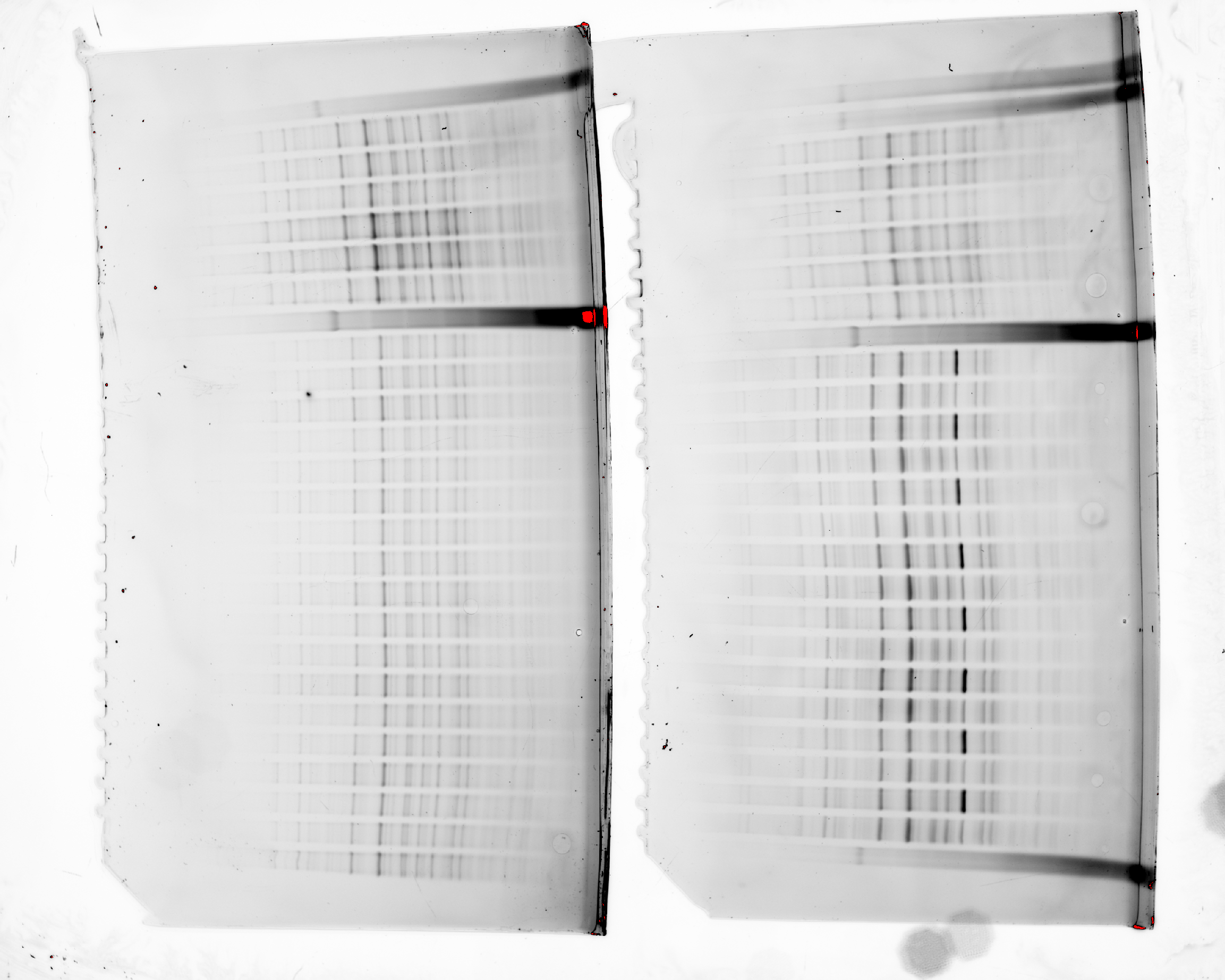

Supplement: Figure 3—source data 2. [file elife-89606-fig3-data2.zip › Figure 3-source data 2/BaldridgeLab 2022-06-16 10h43m24s Stain Free Gel 15.341s(Stain Free Gel).tif]

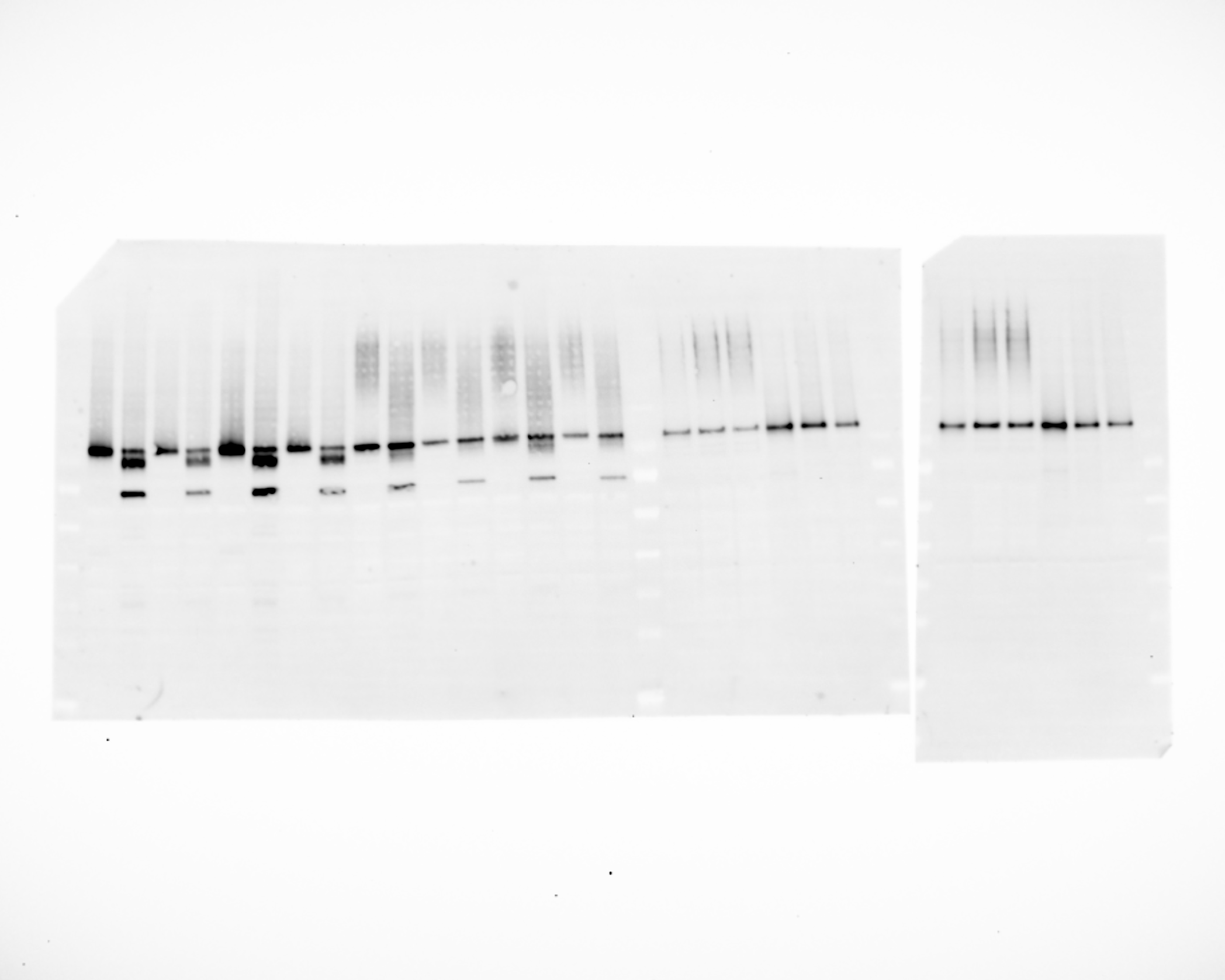

Supplement: Figure 3—source data 2. [file elife-89606-fig3-data2.zip › Figure 3-source data 2/BaldridgeLab 2022-06-16 14h04m35s DyLight 800 50.000s(DyLight 800).tif]

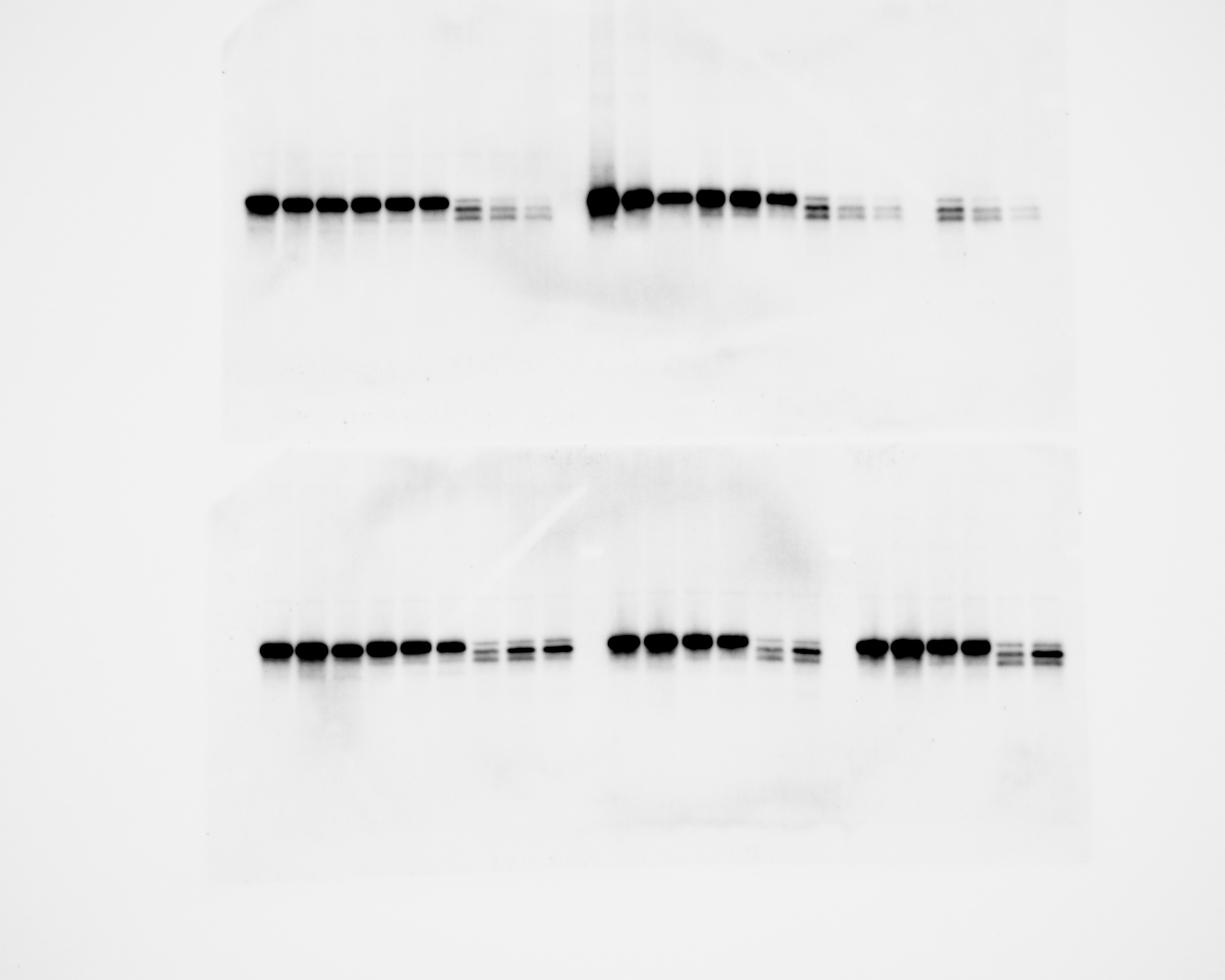

Supplement: Figure 4—source data 2. [file elife-89606-fig4-data2.zip › Figure 4-source data 2/BaldridgeLab 2023-01-06 14h07m50s Chemiluminescence 25.000s(Chemiluminescence).tif]

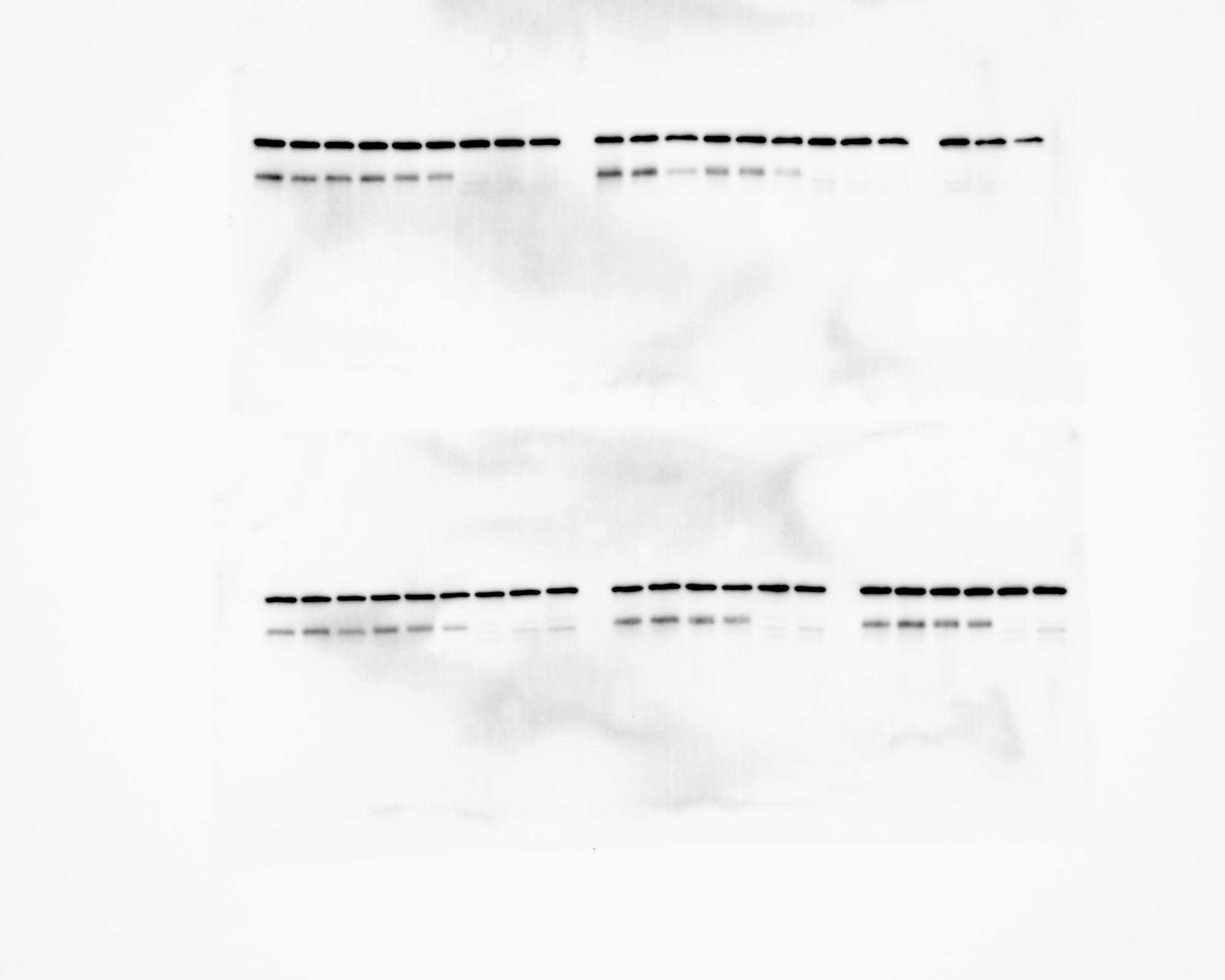

Supplement: Figure 4—source data 2. [file elife-89606-fig4-data2.zip › Figure 4-source data 2/BaldridgeLab 2023-01-06 16h13m20s Chemiluminescence 20.000s(Chemiluminescence).tif]

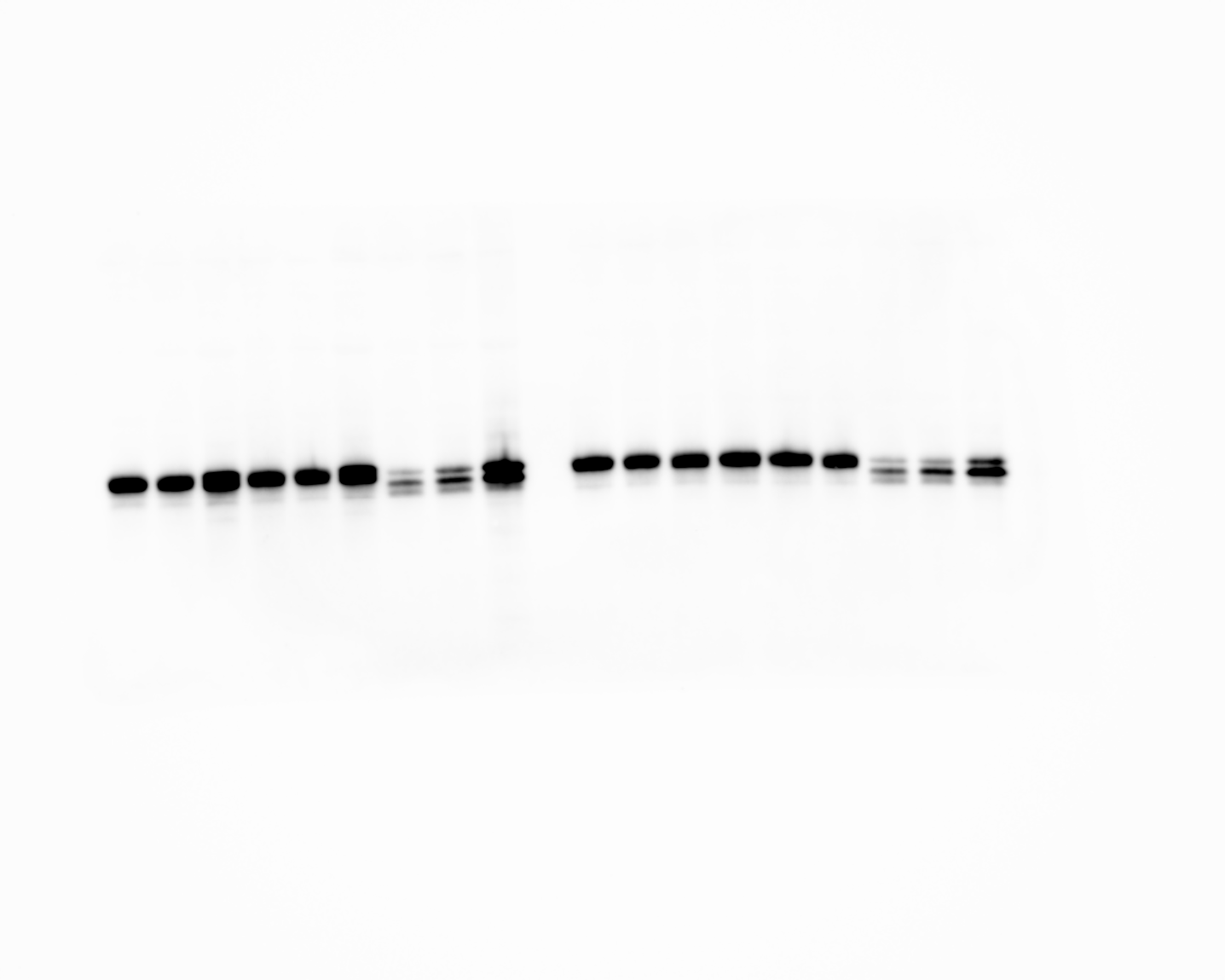

Supplement: Figure 4—source data 2. [file elife-89606-fig4-data2.zip › Figure 4-source data 2/BaldridgeLab 2023-02-03 14h06m08s Chemiluminescence 12.000s(Chemiluminescence).tif]

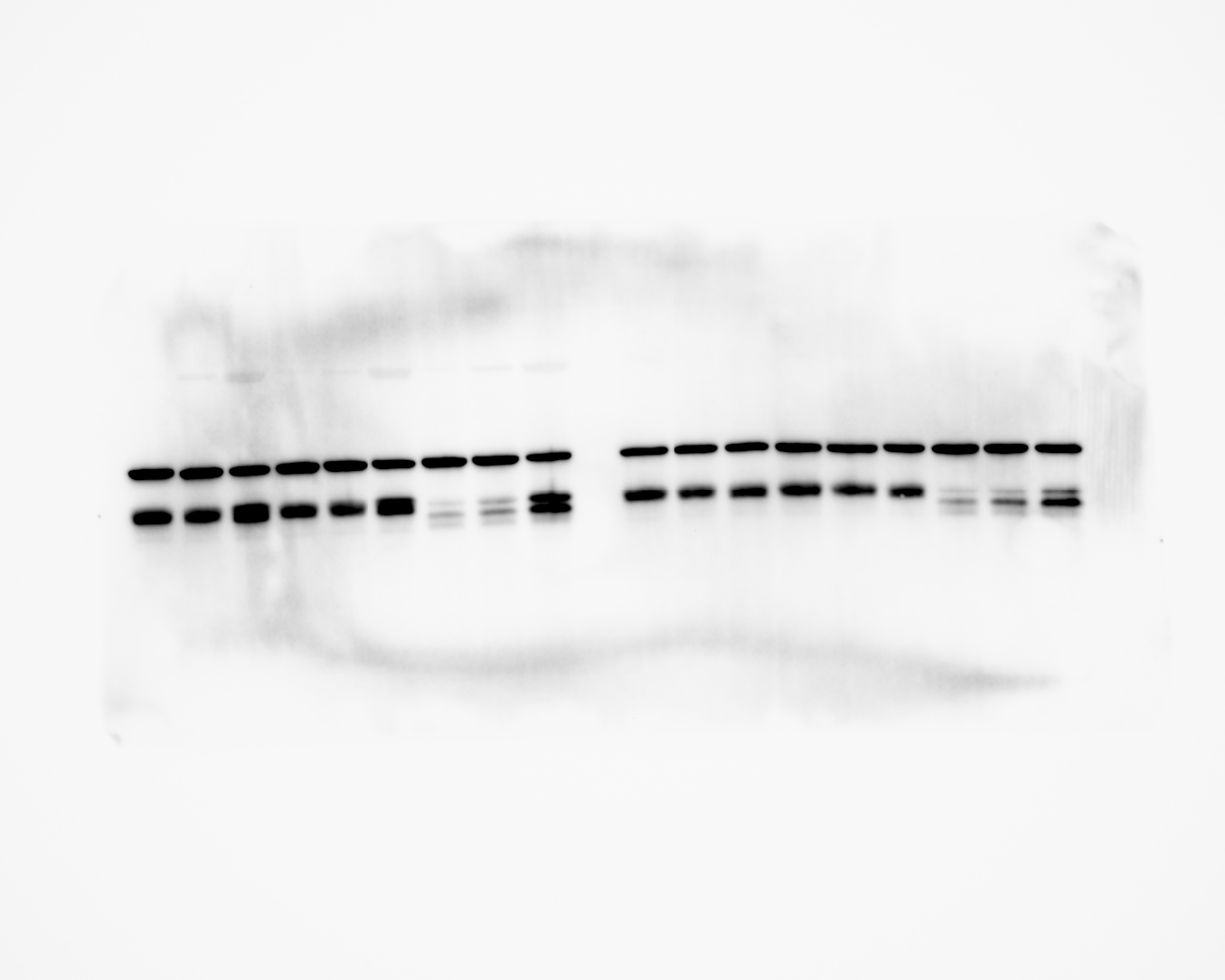

Supplement: Figure 4—source data 2. [file elife-89606-fig4-data2.zip › Figure 4-source data 2/BaldridgeLab 2023-02-03 15h56m02s Chemiluminescence 12.000s(Chemiluminescence).tif]

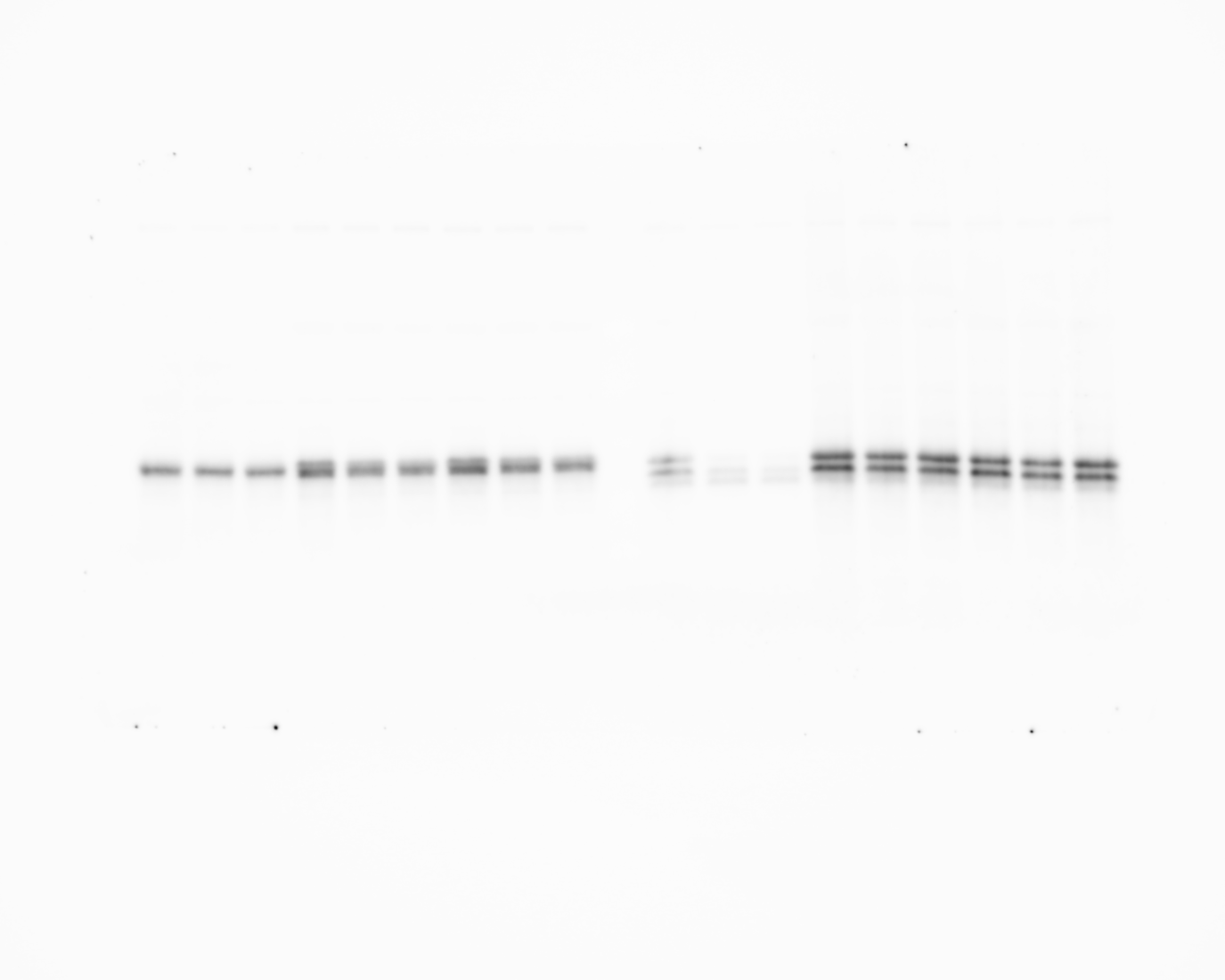

Supplement: Figure 4—source data 2. [file elife-89606-fig4-data2.zip › Figure 4-source data 2/BaldridgeLab 2023-02-05 10h48m33s Chemiluminescence 25.000s(Chemiluminescence).raw16.tif]

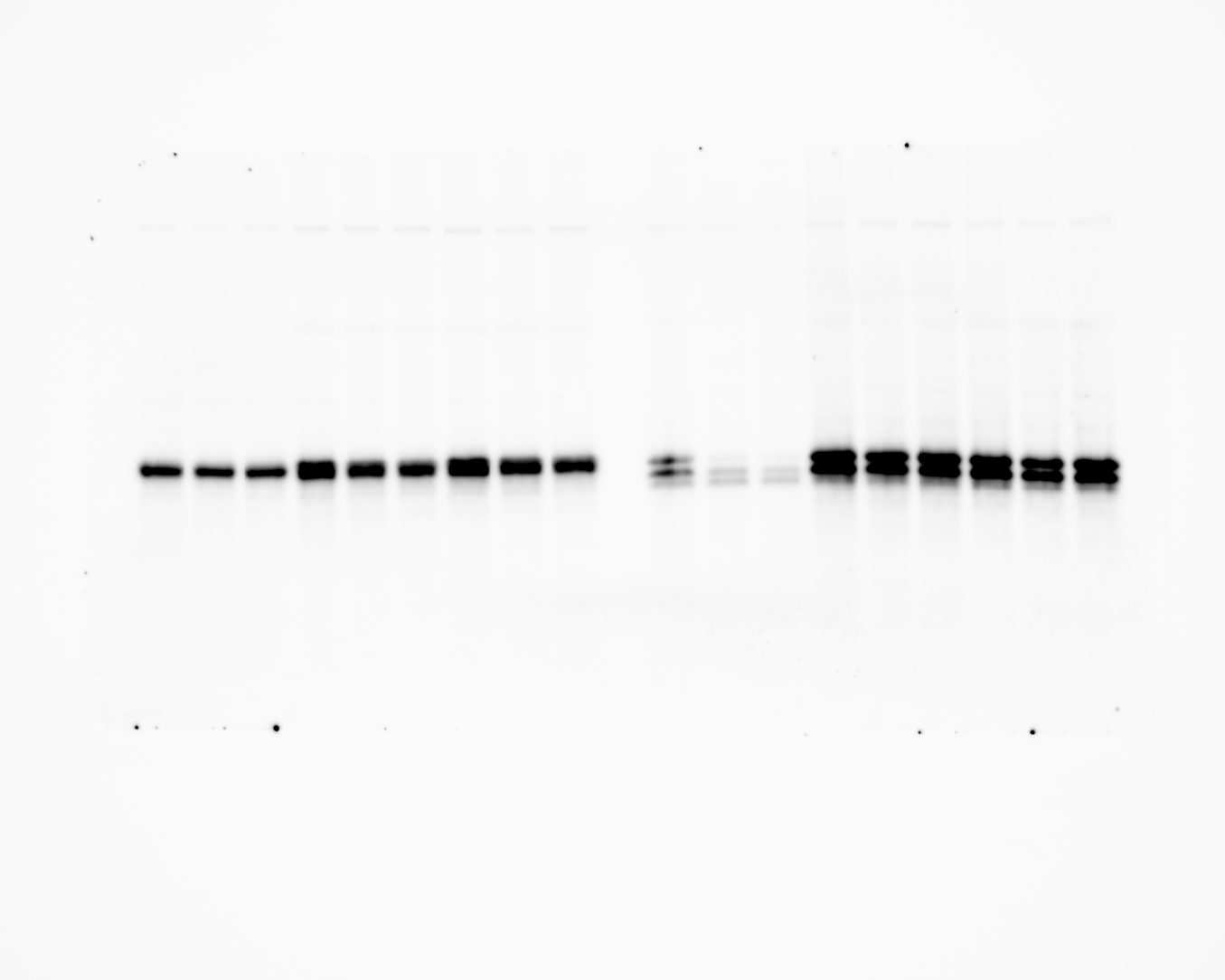

Supplement: Figure 4—source data 2. [file elife-89606-fig4-data2.zip › Figure 4-source data 2/BaldridgeLab 2023-02-05 10h48m33s Chemiluminescence 25.000s(Chemiluminescence).tif]

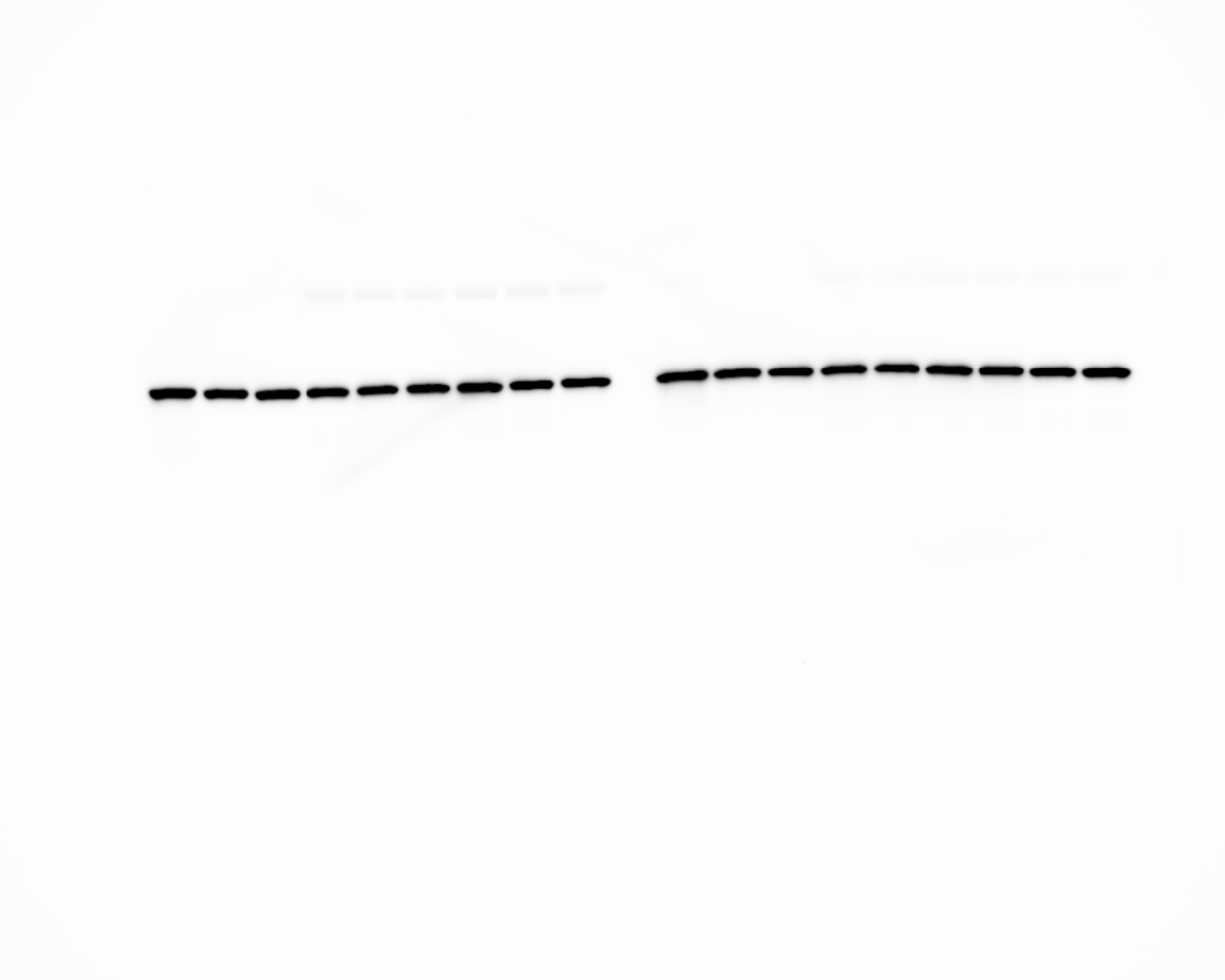

Supplement: Figure 4—source data 2. [file elife-89606-fig4-data2.zip › Figure 4-source data 2/BaldridgeLab 2023-02-06 10h59m06s Chemiluminescence 10.000s(Chemiluminescence).tif]

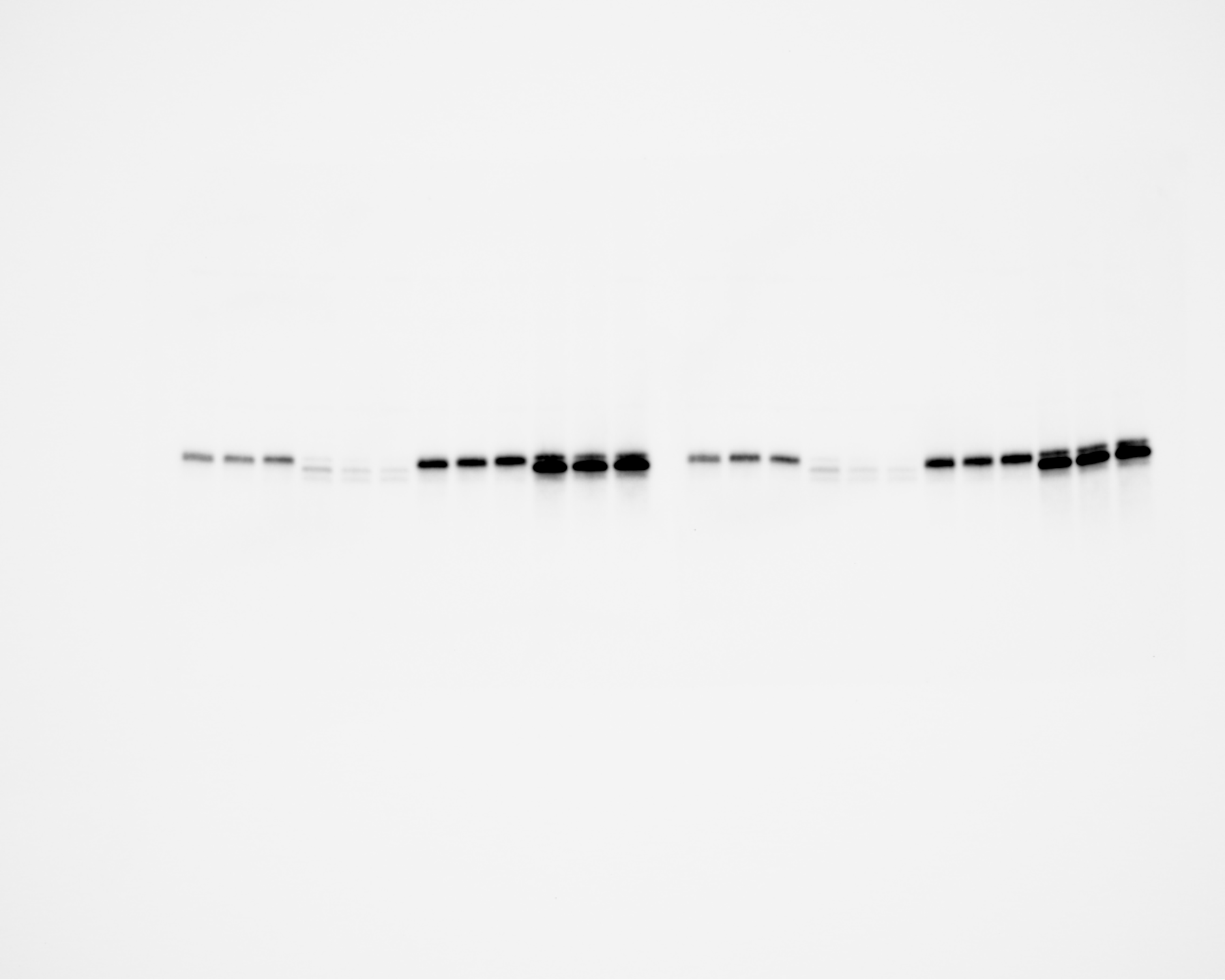

Supplement: Figure 4—source data 2. [file elife-89606-fig4-data2.zip › Figure 4-source data 2/BaldridgeLab 2023-05-15 13h25m52s Chemiluminescence 15.000s(Chemiluminescence).tif]

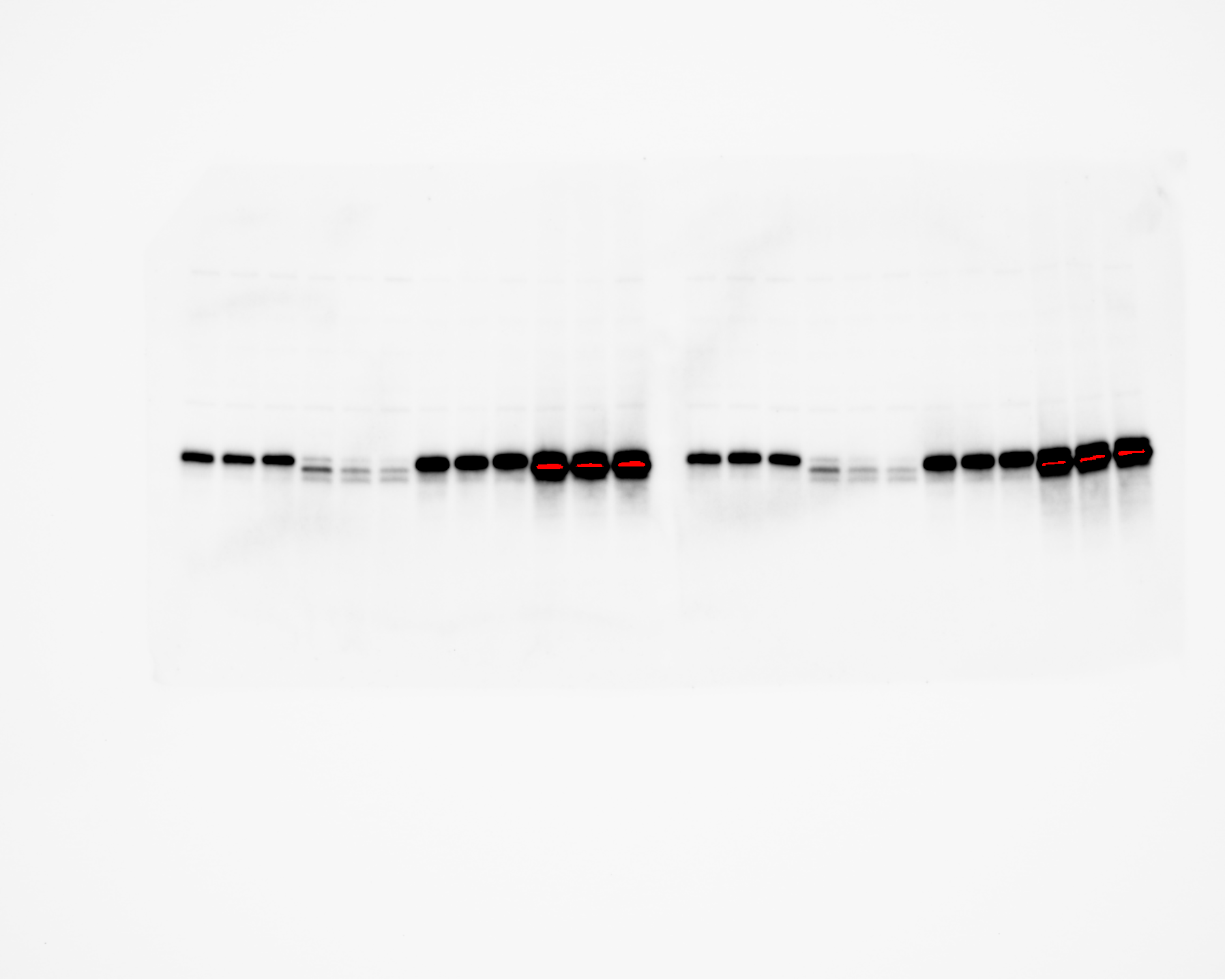

Supplement: Figure 4—source data 2. [file elife-89606-fig4-data2.zip › Figure 4-source data 2/BaldridgeLab 2023-05-15 13h27m08s Chemiluminescence 60.000s(Chemiluminescence).tif]

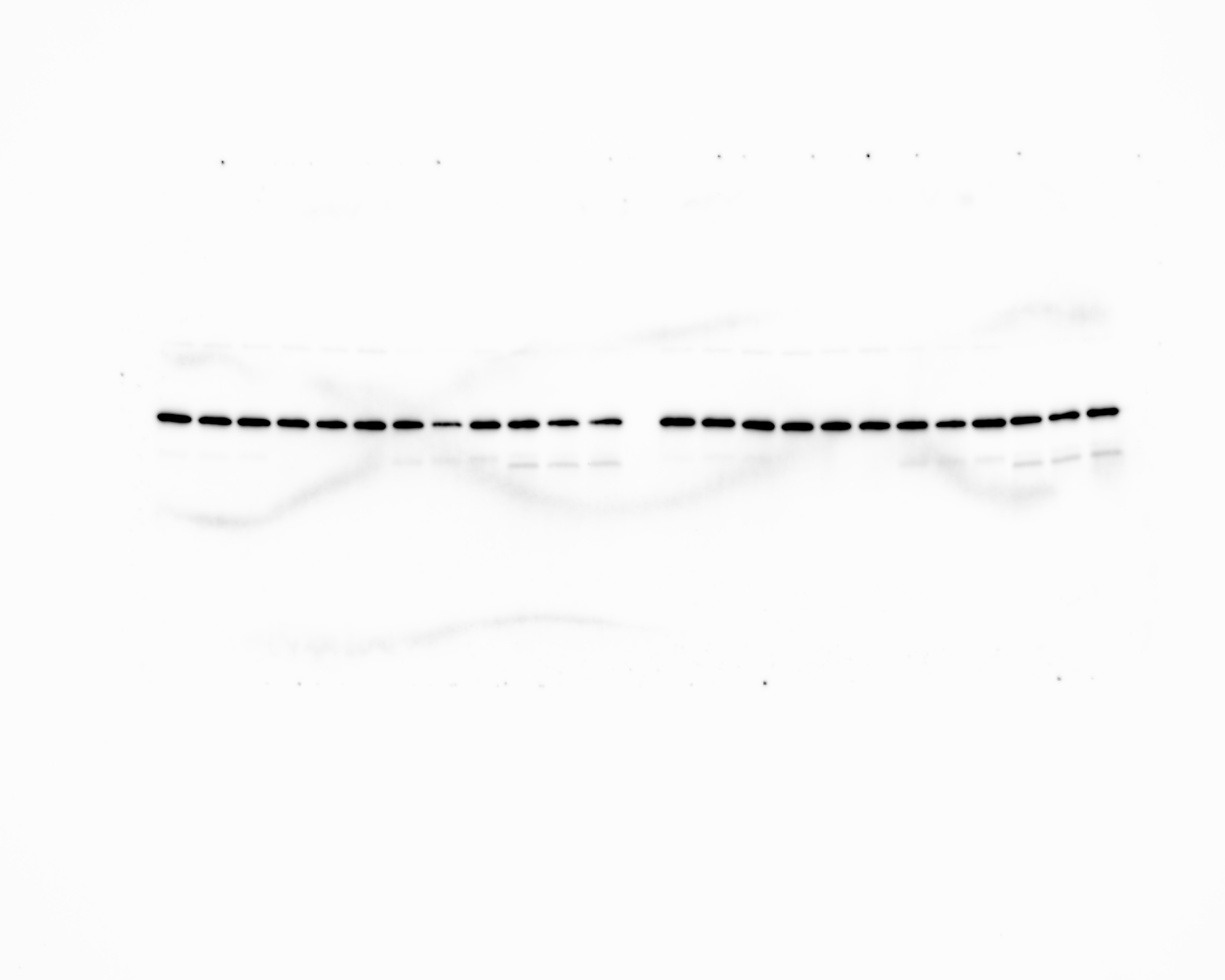

Supplement: Figure 4—source data 2. [file elife-89606-fig4-data2.zip › Figure 4-source data 2/BaldridgeLab 2023-05-16 10h48m00s Chemiluminescence 45.000s(Chemiluminescence).tif]
