# Supplementary material for: Identification of ERAD-dependent degrons for the endoplasmic reticulum lumen
Source: eLife. 2024 Nov 12;12:RP89606. doi: 10.7554/eLife.89606 (PMC11556787; doi:10.7554/eLife.89606)

Figure 2. DegV1 is an ERAD-dependent degron degraded by the cytosolic proteasome.

A

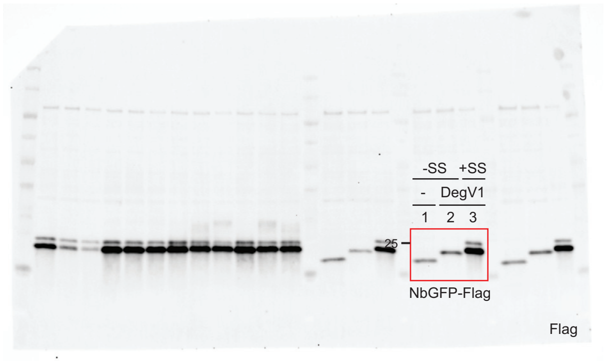

B

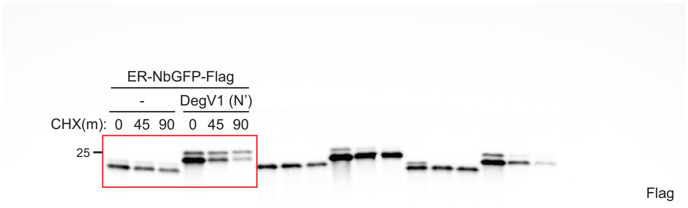

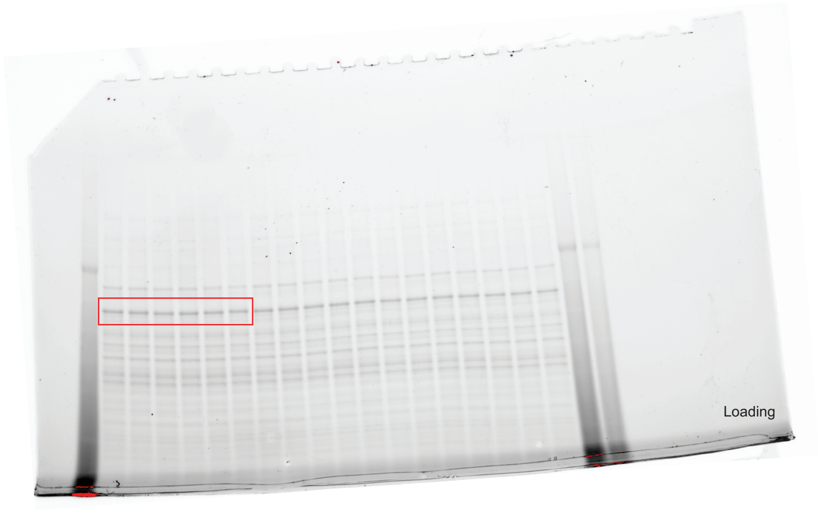

Figure 2. DegV1 is an ERAD-dependent degron degraded by the cytosolic proteasome.

C

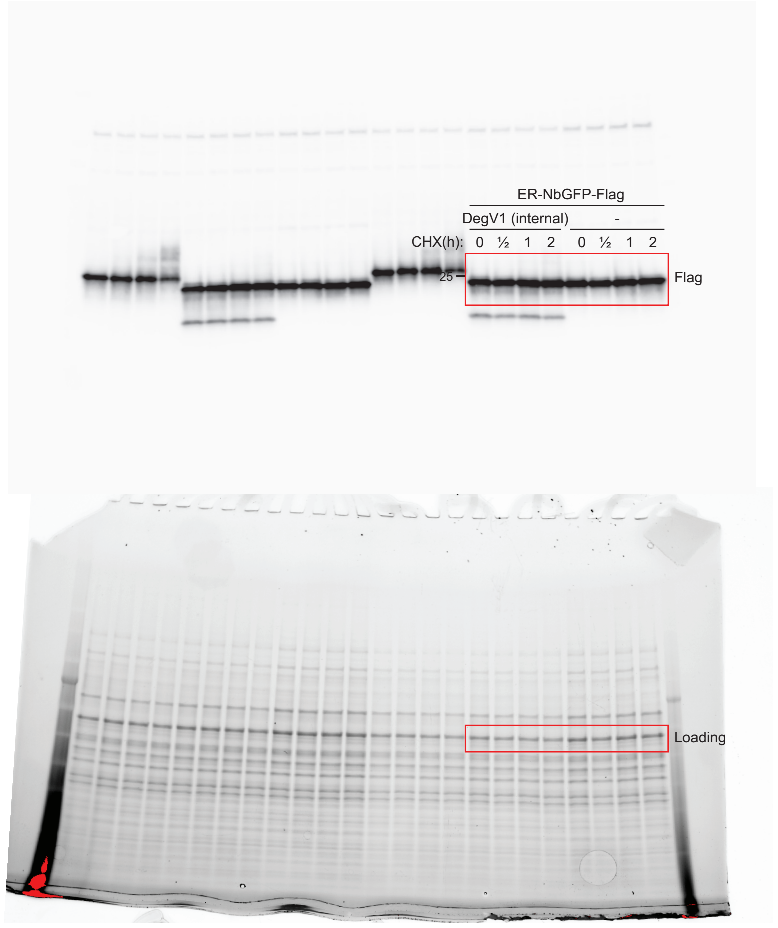

D

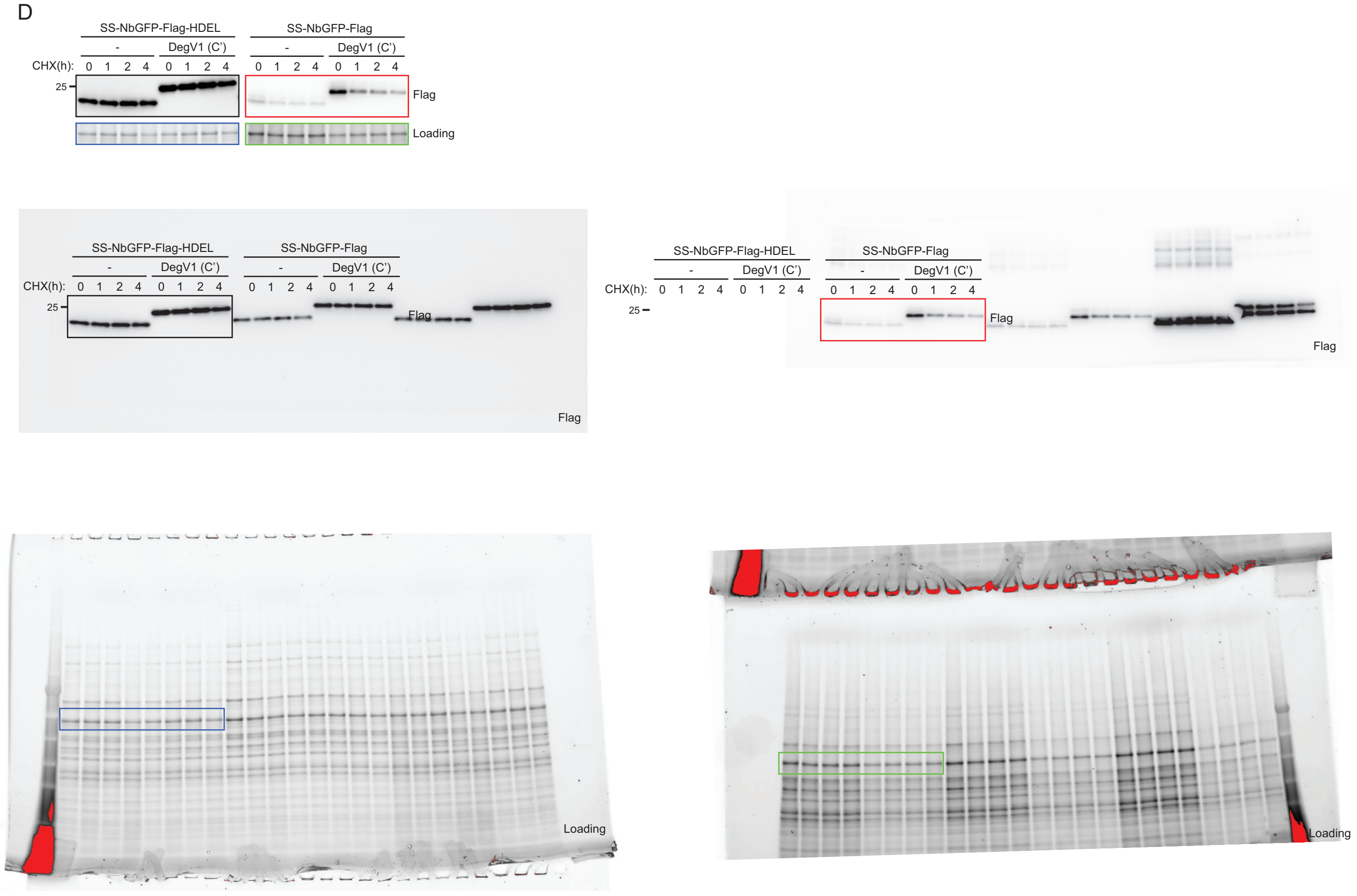

Figure 2. DegV1 is an ERAD-dependent degron degraded by the cytosolic proteasome.

G

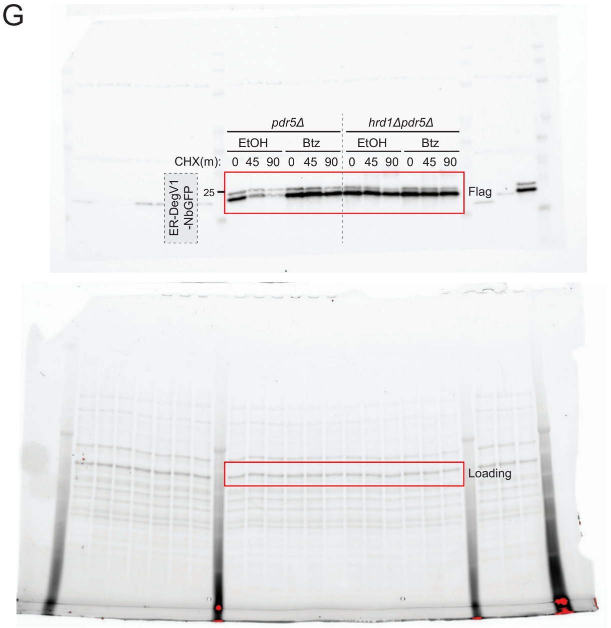

I

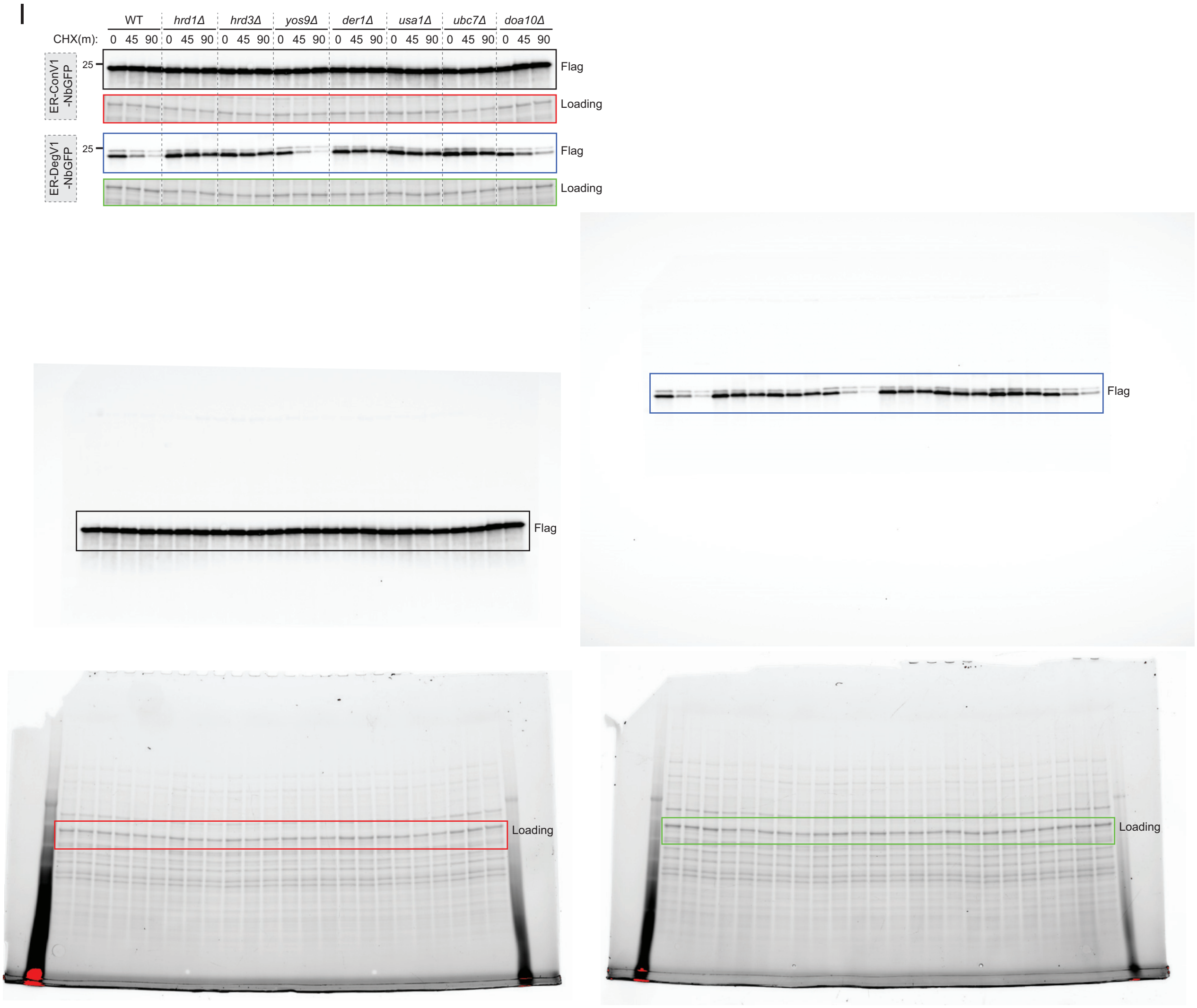

Supplement: Figure 2—source data 1. [file elife-89606-fig2-data1.zip › Figure 2-source data 1.pdf]
