# Supplementary material for: Identification of ERAD-dependent degrons for the endoplasmic reticulum lumen
Source: eLife. 2024 Nov 12;12:RP89606. doi: 10.7554/eLife.89606 (PMC11556787; doi:10.7554/eLife.89606)

Figure 3. DegV targets endogenous ER proteins for degradation

A

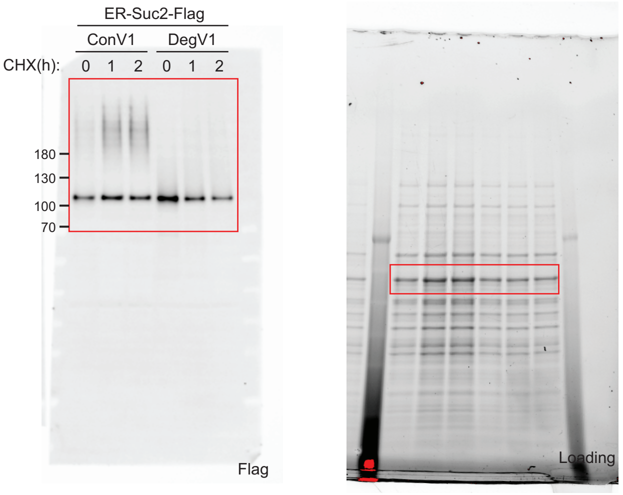

B

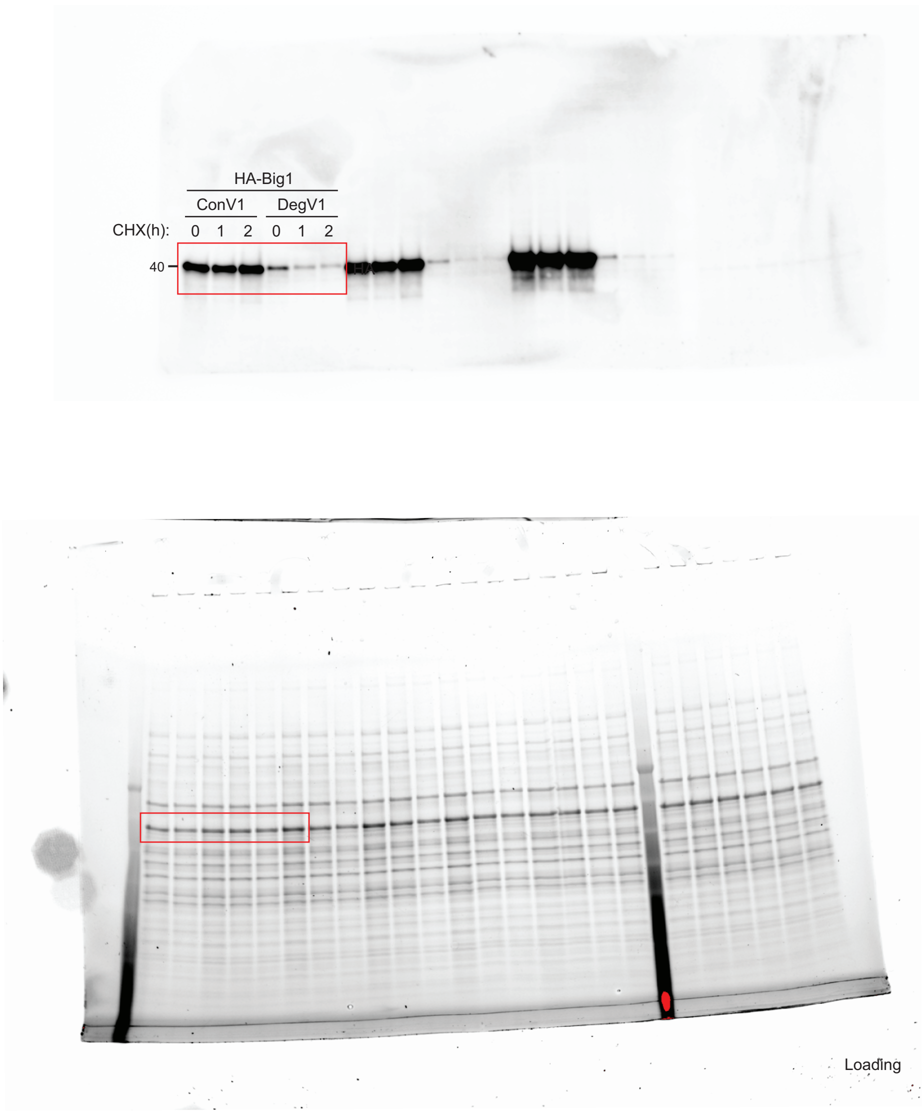

Figure 3. DegV targets endogenous ER proteins for degradation

C

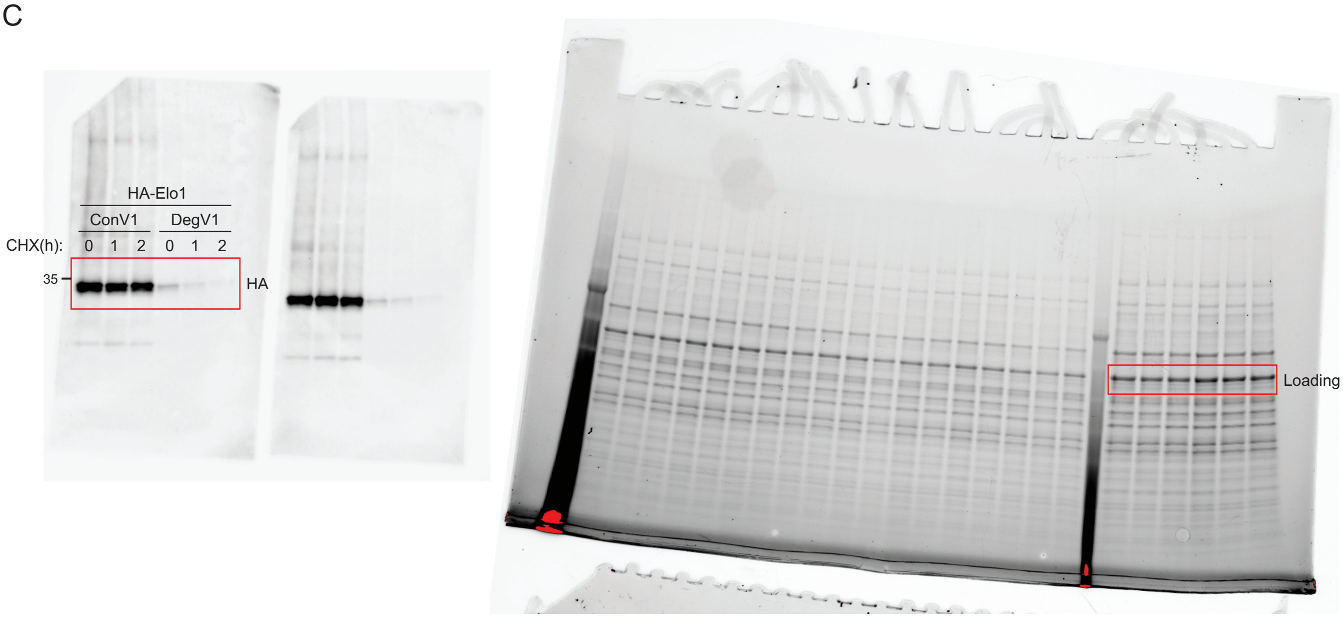

D

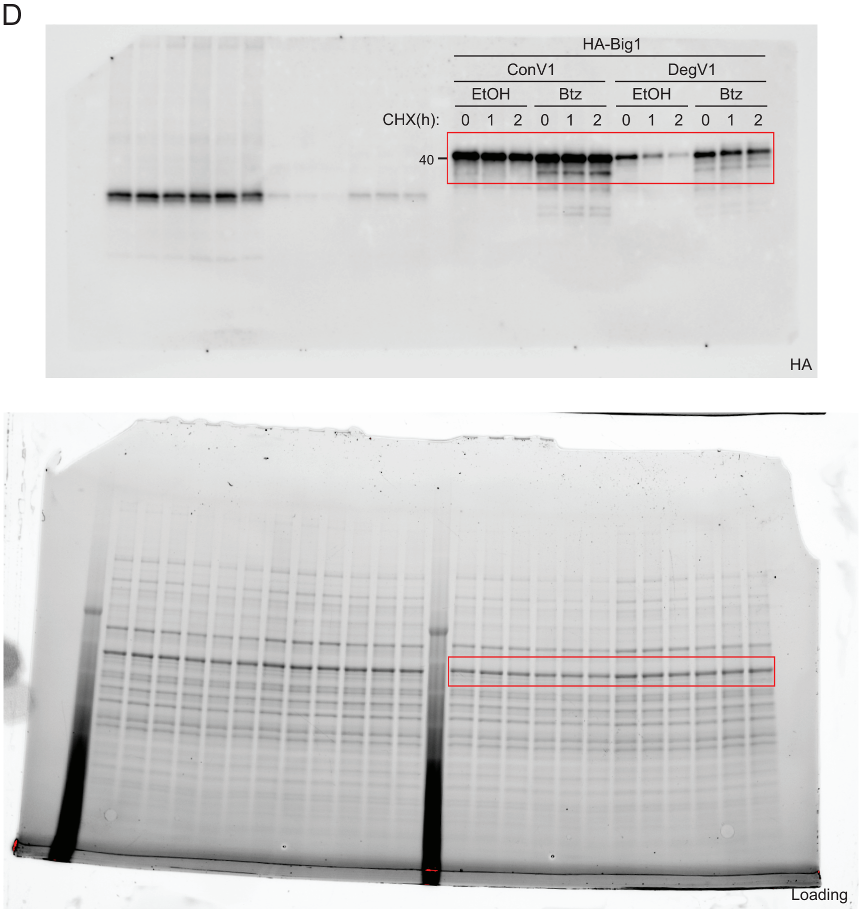

Figure 3. DegV targets endogenous ER proteins for degradation

E

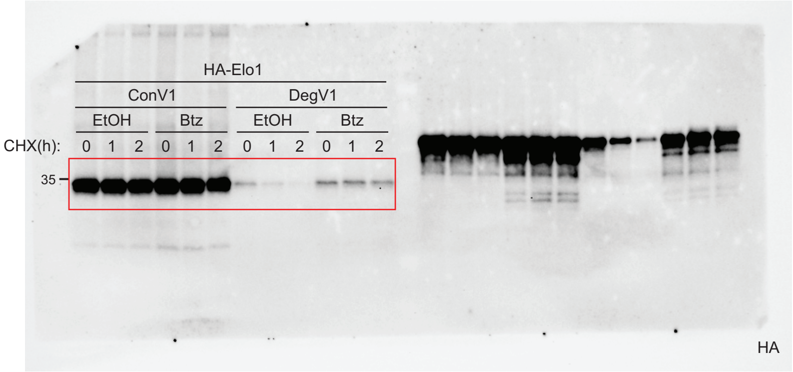

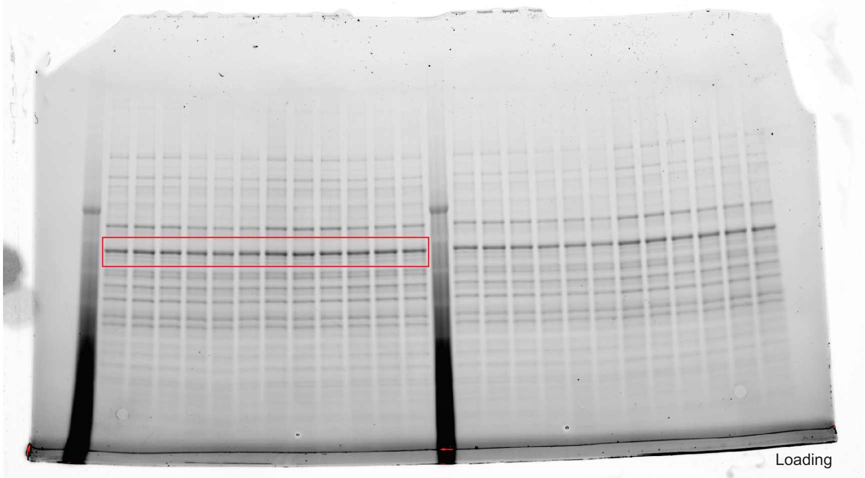

Supplement: Figure 3—source data 1. [file elife-89606-fig3-data1.zip › Figure 3-source data 1.pdf]
