# Supplementary material for: Identification of ERAD-dependent degrons for the endoplasmic reticulum lumen
Source: eLife. 2024 Nov 12;12:RP89606. doi: 10.7554/eLife.89606 (PMC11556787; doi:10.7554/eLife.89606)

Figure 4. DegV1 functions as an ER degron in mammalian cells.

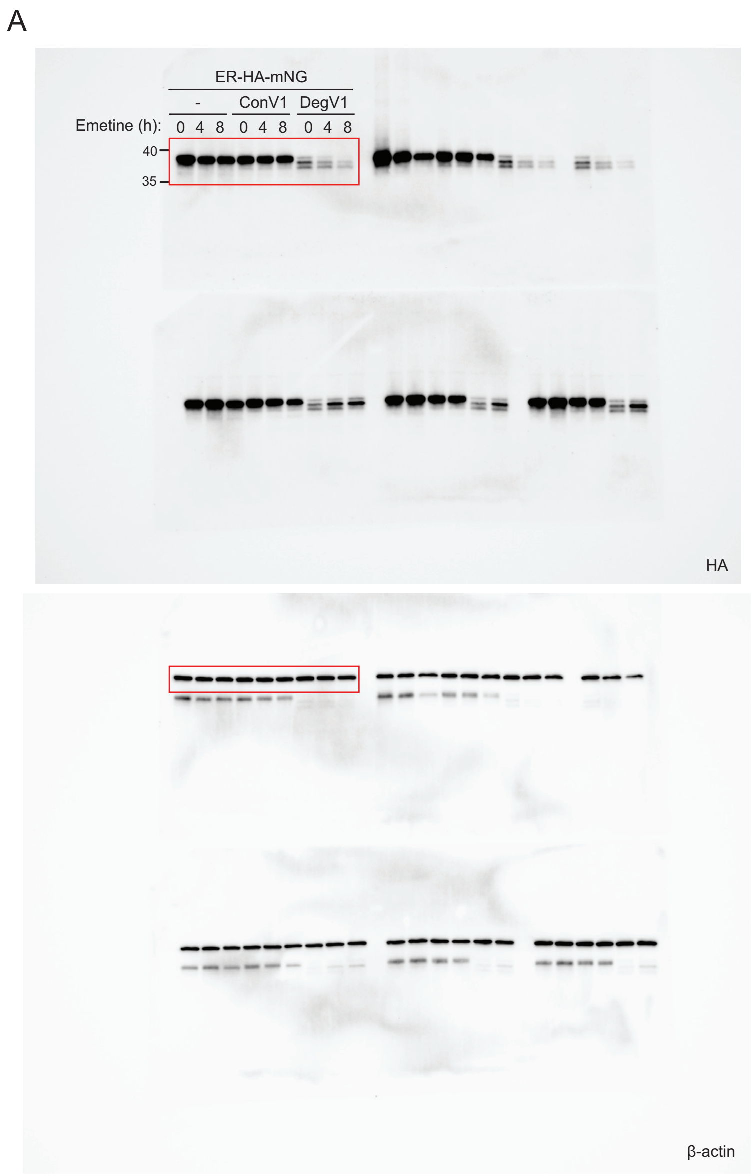

Figure 4. DegV1 functions as an ER degron in mammalian cells.

C

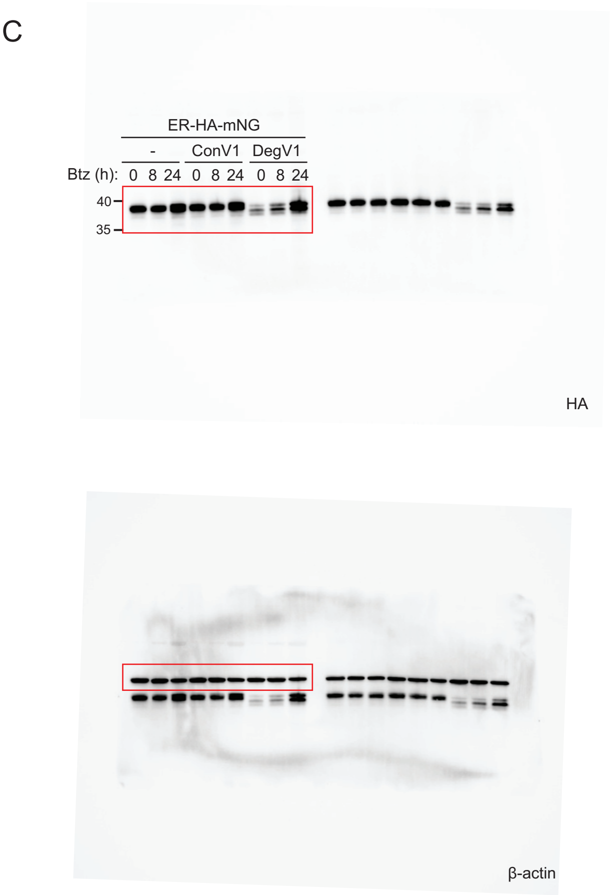

Figure 4. DegV1 functions as an ER degron in mammalian cells.

E

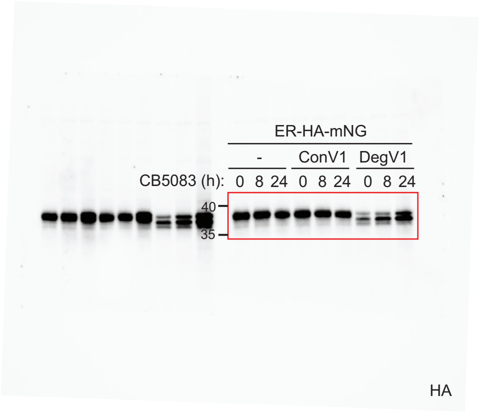

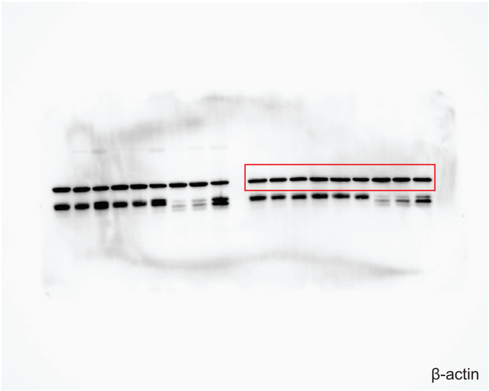

Figure 4. DegV1 functions as an ER degron in mammalian cells.

G

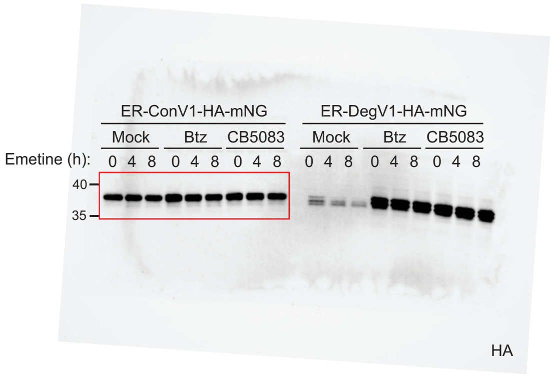

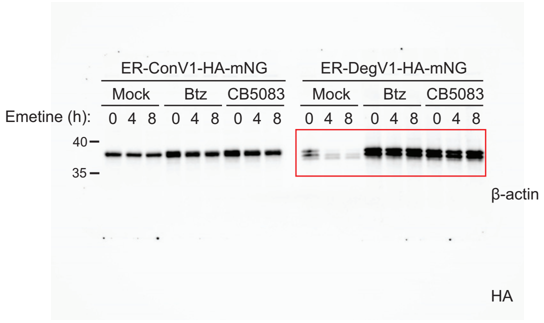

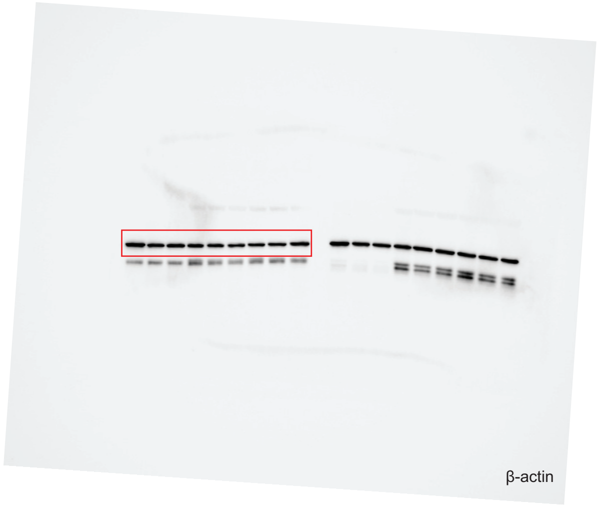

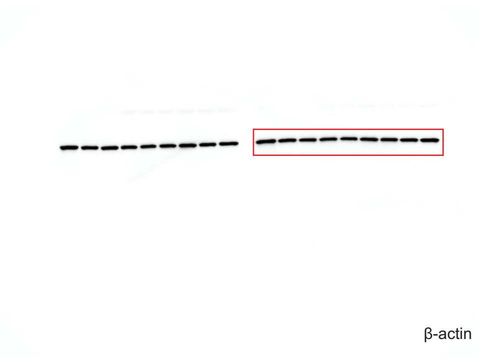

Figure 4. DegV1 functions as an ER degron in mammalian cells.

H

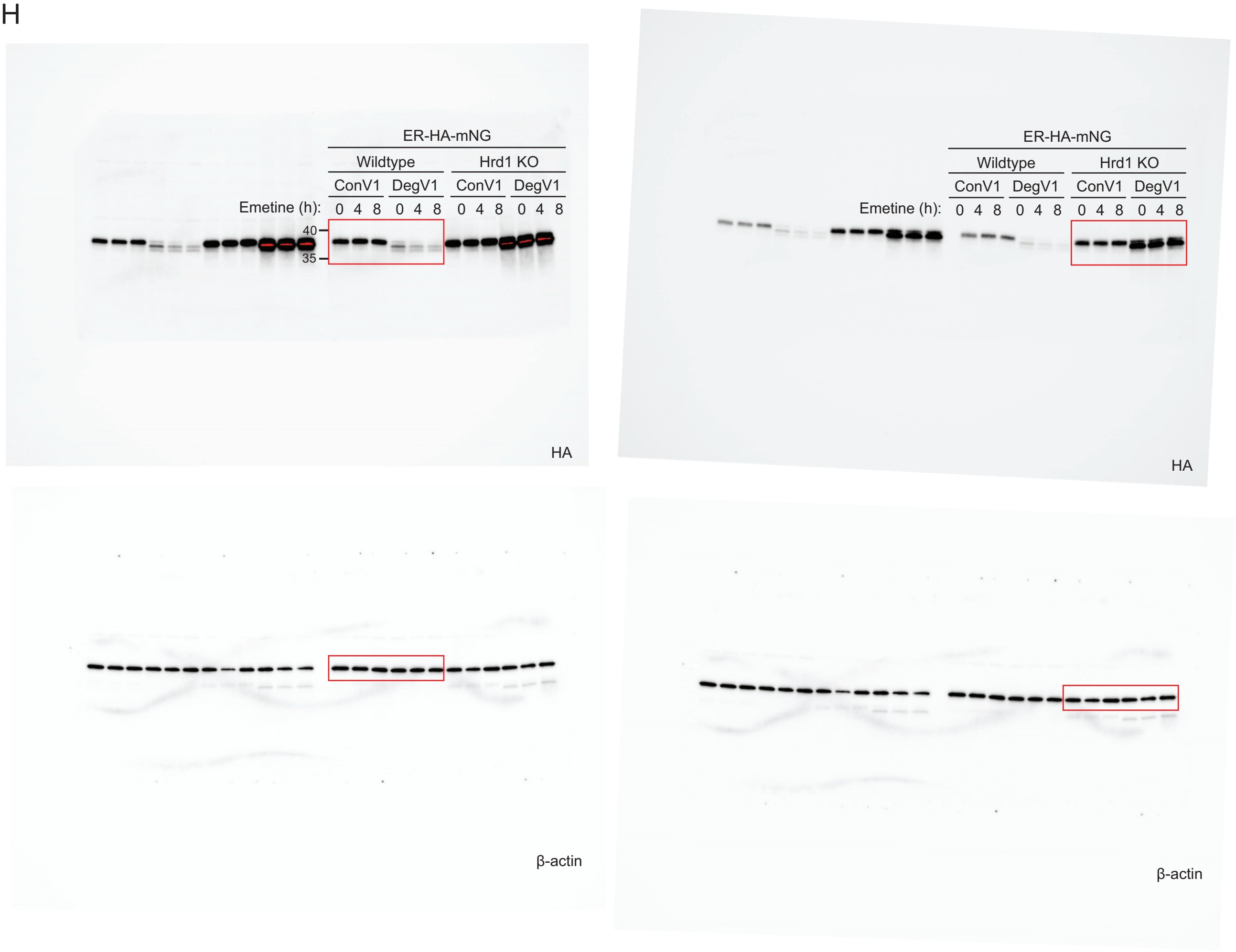

Supplement: Figure 4—source data 1. [file elife-89606-fig4-data1.zip › Figure 4-source data 1.pdf]
